# Supplementary material for: 1-(Arylsulfonyl-isoindol-2-yl)piperazines as 5-HT6R Antagonists: Mechanochemical Synthesis, In Vitro Pharmacological Properties and Glioprotective Activity
Source: Biomolecules. 2022 Dec 21;13(1):12. doi: 10.3390/biom13010012 (PMC9855333; doi:10.3390/biom13010012)
Supplement: Supplementary file 1 [file biomolecules-13-00012-s001.zip › biomolecules-2094241-supplementary.pdf]

1-(Arylsulfonyl-isoindol-2-yl)piperazines as 5-HT$_6$R antagonists: mechanochemical synthesis, *in vitro* pharmacological properties and glioprotective activity

Vittorio Canale [1,*], Wojciech Trybała [1], Séverine Chaumont-Dubel [2], Paulina Koczurkiewicz-Adamczyk [3], Grzegorz Satała [4], Ophélie Bento [2,5], Klaudia Blicharz-Futera [1], Xavier Bantreil [5,6], Elżbieta Pękala [3], Andrzej J. Bojarski [4], Frédéric Lamaty[5], Philippe Marin [2] and Paweł Zajdel [1]

[1] Department of Organic Chemistry, Faculty of Pharmacy, Jagiellonian University Medical College, 9 Medyczna Street, 30-688 Krakow, Poland
[2] Institut de Génomique Fonctionelle, Université de Montpellier, CNRS, INSERM, 34094 Montpellier, France
[3] Department of Pharmaceutical Biochemisty, Faculty of Pharmacy, Jagiellonian University Medical College, 9 Medyczna Street, 30-688 Krakow, Poland
[4] Department of Medicinal Chemistry, Maj Institute of Pharmacology, Polish Academy of Sciences, 12 Smętna Street, 31-343 Krakow, Poland
[5] IBMM, Université de Montpellier, CNRS, ENSCM, 34095 Montpellier, France
[6] Institut Universitaire de France (IUF), 75005 Paris, France
* Correspondence: vittorio.canale@uj.edu.pl

Table of contents

Characterization Data

1. Characterization data for intermediate compounds **2a**–**l** 3
2. UPLC/MS, ^{1}H NMR and ^{13}C NMR spectra of intermediates **2a**–**l** 7
3. UPLC/MS, ^{1}H NMR and ^{13}C NMR spectra of final compounds **3a**–**l** 31

Supporting Materials

Table S1–SI. Optimization of milling conditions for sulfonylation of isoindolines **1a**–**d** 55
Table S2–SI. Optimization of milling conditions for S$_N$Ar of intermediates **2a**–**l** 56
Figure S1–SI. The effect of compound **3g** on cellular viability 57

Procedures

1. Procedure for radioligand binding assays 58
2. Procedure for determination of functional activity at Gs signaling 59
3. Procedure for evaluation of the effect of tested compounds on Cdk5-dependent neurite growth 61

References 62

1. Characterization data for intermediate compounds 2a–l

1.1. 2-[(4-Fluorophenyl)sulfonyl]isoindoline **2a**

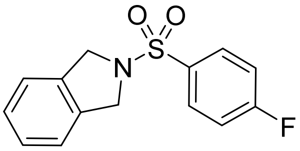

White solid, 194 mg .(isolated yield 98%); UPLC/MS purity 100%, t_R = 7.29; $C_{14}H_{12}FNO_2S$, MW 277.31, Monoisotopic Mass 277.06, $[M+H]^+$ 278.2. ^{1}H NMR (500 MHz, $CDCl_3$) δ ppm 4.62 (s, 4H), 7.15–7.21 (m, 4H), 7.22–7.26 (m, 2H), 7.87–7.93 (m, 2H). ^{13}C NMR (175 MHz, $CDCl_3$) δ ppm 53.8, 116.6 (d, $J_{C\text{-}F}$ = 22.3 Hz), 122.8, 127.9, 130.3 (d, $J_{C\text{-}F}$ = 9.1 Hz), 133.1 (d, $J_{C\text{-}F}$ = 3.0 Hz), 135.9, 165.3(d, $J_{C\text{-}F}$ = 254.7 Hz).

1.2. 4-Chloro-2-[(4-fluorophenyl)sulfonyl]isoindoline **2b**

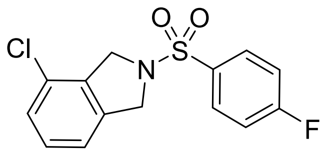

White solid, 168 mg (isolated yield 81%); UPLC/MS purity 100%, t_R = 8.07; $C_{14}H_{11}ClFNO_2S$, MW 311.76, Monoisotopic Mass 311.02, $[M+H]^+$ 312.1. ^{1}H NMR (500 MHz, $CDCl_3$) δ ppm 4.60–4.64 (m, 2H), 4.65–4.69 (s, 2H), 7.02–7.09 (m, 1H), 7.16–7.27 (m, 4H), 7.88–7.93 (m, 2H). ^{13}C NMR (126 MHz, $CDCl_3$) δ ppm 53.4, 54.5, 116.7 (d, $J_{C\text{-}F}$ = 2.3 Hz), 121.0, 128.0, 129.2, 129.7, 130.3 (d, $J_{C\text{-}F}$ = 9.7 Hz), 133.0 (d, $J_{C\text{-}F}$ = 3.0 Hz), 134.8, 137.8, 165.4 (d, $J_{C\text{-}F}$ = 255.3 Hz).

1.3. 4-Bromo-2-[(4-fluorophenyl)sulfonyl]isoindoline **2c**

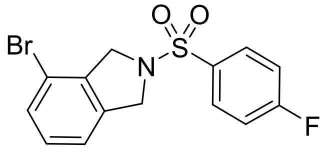

White solid, 177 mg (isolated yield 81%); UPLC/MS purity 100%, t_R = 8.19; $C_{14}H_{11}BrFNO_2S$, MW 356.21, Monoisotopic Mass 354.97, $[M+H]^+$ 356.0/358.0. ^{1}H NMR (500 MHz, $CDCl_3$) δ ppm 4.58 (t, J = 2.0 Hz, 2H), 4.70 (s, 2H), 7.08–7.17 (m, 2H), 7.18–7.24 (m, 2H), 7.33–7.39 (m, 1H), 7.88–7.94 (m, 2H). ^{13}C NMR (175 MHz, $CDCl_3$) δ ppm 54.7, 55.1, 116.7 ($J_{C\text{-}F}$ = 22.9 Hz), 117.5, 121.5, 129.8, 130.3 ($J_{C\text{-}F}$ = 9.7 Hz), 131.0, 133.0 ($J_{C\text{-}F}$ = 3.6 Hz), 136.9, 137.6, 165.4 ($J_{C\text{-}F}$ = 255.3 Hz).

1.4. 5-Bromo-2-[(4-fluorophenyl)sulfonyl]isoindoline **2d**

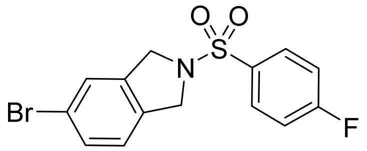

White solid, 189 mg (isolated yield 86%); UPLC/MS purity 98%, t_R = 8.05; $C_{14}H_{11}BrFNO_2S$, MW 356.21, Monoisotopic Mass 354.97, $[M+H]^+$ 356.0/358.1. ^1H NMR (500 MHz, $CDCl_3$) δ ppm 4.55 (s, 2H), 4.59 (s, 2H), 7.04 (d, J = 8.3 Hz, 1H), 7.16–7.25 (m, 2H), 7.31 (s, 1H), 7.33–7.38 (m, 1H), 7.85–7.91 (m, 2H). ^{13}C NMR (126 MHz, $CDCl_3$) δ ppm 53.3, 53.4, 116.7 ($J_{C\text{-}F}$ = 22.9 Hz), 121.7, 124.3, 126.0, 130.2 ($J_{C\text{-}F}$ = 9.1 Hz), 131.1, 132.9 ($J_{C\text{-}F}$ = 3.0 Hz), 134.9, 138.2, 165.4 ($J_{C\text{-}F}$ = 255.3 Hz).

1.5. 2-[(3-Fluorophenyl)sulfonyl]isoindoline **2e**

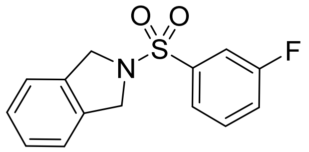

White solid, 190 mg (isolated yield 96%); UPLC/MS purity 100%, t_R = 7.39; $C_{14}H_{12}FNO_2S$, MW 277.31, Monoisotopic Mass 277.06, $[M+H]^+$ 278.2. ^1H NMR (500 MHz, $CDCl_3$) δ ppm 4.64 (s, 4H), 7.16–7.20 (m, 2H), 7.22–7.26 (m, 2H), 7.26–7.29 (m, 1H), 7.51 (td, J = 8.1, 5.3 Hz, 1H), 7.59 (dq, J = 8.3, 1.5 Hz, 1H), 7.67 (dq, J = 7.7, 0.9 Hz, 1H). ^{13}C NMR (175 MHz, $CDCl_3$) δ ppm 53.9, 114.9 ($J_{C\text{-}F}$ = 24.1 Hz), 120.2 ($J_{C\text{-}F}$ = 21.1 Hz), 122.8, 123.3 ($J_{C\text{-}F}$ = 3.0 Hz), 128.0, 131.1 ($J_{C\text{-}F}$ = 7.8 Hz), 135.8, 139.0 ($J_{C\text{-}F}$ = 6.6 Hz), 162.7 ($J_{C\text{-}F}$ = 252.3 Hz).

1.6. 4-Chloro-2-[(3-fluorophenyl)sulfonyl]isoindoline **2f**

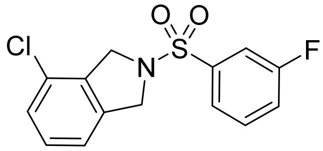

White solid, 156 mg (isolated yield 75%); UPLC/MS purity 100%, t_R = 8.13; $C_{14}H_{11}ClFNO_2S$, MW 311.76, Monoisotopic Mass 311.02, $[M+H]^+$ 312.1. ^1H NMR (500 MHz, $CDCl_3$) δ ppm 4.61–4.66 (m, 2H), 4.67–4.71 (m, 2H), 7.03–7.10 (m, 1H), 7.17–7.22 (m, 2H), 7.26–7.32 (m, 1H), 7.49–7.55 (m, 1H), 7.57–7.61 (m, 1H), 7.66–7.70 (m, 1H). ^{13}C NMR (126 MHz, $CDCl_3$) δ ppm 53.4, 54.5, 114.9 (d, $J_{C\text{-}F}$ = 24.7 Hz), 120.3 (d, $J_{C\text{-}F}$ = 21.1 Hz), 121.0, 123.3 (d, $J_{C\text{-}F}$ = 3.0 Hz), 128.0, 129.2, 129.7, 131.2 (d, $J_{C\text{-}F}$ = 7.2 Hz), 134.7, 137.8, 138.9 (d, $J_{C\text{-}F}$ = 6.6 Hz), 162.7 (d, $J_{C\text{-}F}$ = 251.7 Hz).

1.7. 4-Bromo-2-[(3-fluorophenyl)sulfonyl]isoindoline **2g**

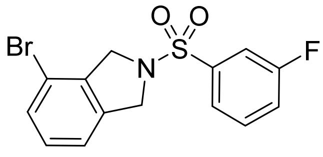

White solid, 173 mg (isolated yield 79%); UPLC/MS purity 100%, t_R = 8.12; $C_{14}H_{11}BrFNO_2S$, MW 356.21, Monoisotopic Mass 354.97, $[M+H]^+$ 356.0/358.0. ^1H NMR (500 MHz, $CDCl_3$) δ ppm 4.60 (t, J = 2.0 Hz, 2H), 4.72 (t, J = 2.0 Hz, 2H), 7.09–7.14 (m, 2H), 7.29 (tdd, J = 8.3, 2.6, 0.9 Hz, 1H), 7.35–7.38 (m, 1H), 7.53 (td, J = 8.0, 5.4 Hz, 1H), 7.60 (ddd, J = 8.0, 2.6, 1.7 Hz, 1H), 7.68 (ddd, J = 7.7, 1.7, 0.9 Hz, 1H). ^{13}C NMR (175 MHz, $CDCl_3$) δ ppm 54.7, 55.1, 114.9 (J_{C-F} = 24.1 Hz), 117.5, 120.3 (J_{C-F} = 21.1 Hz), 121.6, 123.3 (J_{C-F} = 3.6 Hz), 129.8, 131.0, 131.3 (J_{C-F} = 7.2 Hz), 136.8, 137.6, 138.9 (J_{C-F} = 6.6 Hz), 162.7 (J_{C-F} = 251.7 Hz).

1.8. 5-Bromo-2-[(3-fluorophenyl)sulfonyl]isoindoline **2h**

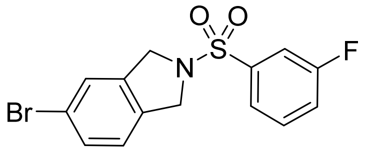

White solid, 186 mg (isolated yield 85%); UPLC/MS purity 100%, t_R = 8.12; $C_{14}H_{11}BrFNO_2S$, MW 356.21, Monoisotopic Mass 354.97, $[M+H]^+$ 356.0/358.0. ^1H NMR (500 MHz, $CDCl_3$) δ ppm 4.58 (d, J = 1.4 Hz, 2H), 4.61 (s, 2H), 7.05 (d, J = 8.3 Hz, 1H), 7.28 (tdd, J = 8.6, 8.6, 2.6, 0.9 Hz, 1H), 7.31–7.33 (m, 1H), 7.34–7.38 (m, 1H), 7.48–7.55 (m, 1H), 7.57 (dt, J = 8.0, 1.7 Hz, 1H), 7.66 (dt, J = 7.8, 1.3 Hz, 1H). ^{13}C NMR (126 MHz, $CDCl_3$) δ ppm 53.4, 53.5, 114.9 (J_{C-F} = 24.1 Hz), 120.3 (J_{C-F} = 21.7 Hz), 121.8, 123.3 (J_{C-F} = 3.0 Hz), 124.3, 126.0, 131.2, 131.3, 134.9, 138.1, 138.8 (J_{C-F} = 6.6 Hz), 162.7 (J_{C-F} = 252.3 Hz).

1.9. 2-[(2-Fluorophenyl)sulfonyl]isoindoline **2i**

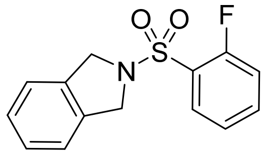

White solid, 184 mg (isolated yield 93%); UPLC/MS purity 100%, t_R = 7.26; $C_{14}H_{12}FNO_2S$, MW 277.31, Monoisotopic Mass 277.06, $[M+H]^+$ 278.2. ^1H NMR (500 MHz, $CDCl_3$) δ ppm 4.75 (s, 4H), 7.14–7.17 (m, 1H), 7.18–7.22 (m, 2H), 7.23–7.29 (m, 3H), 7.52–7.57 (m, 1H), 7.93–7.97 (m, 1H). ^{13}C NMR (175 MHz, $CDCl_3$) δ ppm 53.5, 117.5 (J_{C-F} = 21.7 Hz), 122.7, 124.48 (J_{C-F} = 3.62 Hz), 126.19 (J_{C-F} = 15.1 Hz), 127.9, 131.4, 135.1 (J_{C-F} = 8.45 Hz), 136.0, 159.0 (J_{C-F} = 245.0 Hz).

1.10. 4-Chloro-2-[(2-fluorophenyl)sulfonyl]isoindoline **2j**

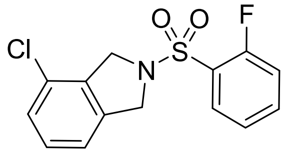

White solid, 164 mg (isolated yield 79%); UPLC/MS purity 100%, t_R = 8.02; $C_{14}H_{11}ClFNO_2S$, MW 311.76, Monoisotopic Mass 311.02, $[M+H]^+$ 312.1. ^1H NMR (500 MHz, $CDCl_3$) δ ppm 4.72–4.75 (m, 2H), 4.80–4.83 (m, 2H), 7.05–7.11 (m, 1H), 7.16–7.23 (m, 3H), 7.29 (td, J = 7.7, 1.1 Hz, 1H), 7.53–7.59 (m, 1H), 7.96 (ddd, J = 7.8, 6.9, 1.9 Hz, 1H). ^{13}C NMR (126 MHz, $CDCl_3$) δ ppm 53.1, 54.2, 117.5 (d, $J_{C\text{-}F}$ = 21.7 Hz), 121.0, 124.6 (d, $J_{C\text{-}F}$ = 3.6 Hz), 126.1 (d, $J_{C\text{-}F}$ = 15.1 Hz), 127.9, 129.2, 129.6, 131.4, 134.9, 135.2 (d, $J_{C\text{-}F}$ = 8.5 Hz), 138.0, 159.0 (d, $J_{C\text{-}F}$ = 255.3 Hz).

1.11. 4-Bromo-2-[(2-fluorophenyl)sulfonyl]isoindoline **2k**

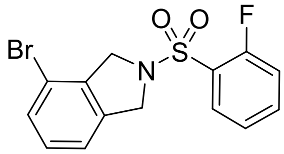

White solid, 180 mg (isolated yield 82%); UPLC/MS purity 100%, t_R = 8.15; $C_{14}H_{11}BrFNO_2S$, MW 356.21, Monoisotopic Mass 354.97, $[M+H]^+$ 356.0/358.0. ^1H NMR (500 MHz, $CDCl_3$) δ ppm 4.70 (s, 2H), 4.85 (s, 2H), 7.12–7.16 (m, 2H), 7.16–7.21 (m, 1H), 7.29 (td, J = 8.0, 1.1 Hz, 1H), 7.35–7.41 (m, 1H), 7.53–7.59 (m, 1H), 7.96 (ddd, J = 7.9, 7.0, 1.7 Hz, 1H). ^{13}C NMR (175 MHz, $CDCl_3$) δ ppm 54.5, 54.8, 117.5 ($J_{C\text{-}F}$ = 23.5 Hz), 117.8, 121.5, 124.6 ($J_{C\text{-}F}$ = 3.6 Hz), 126.1 ($J_{C\text{-}F}$ = 15.1 Hz), 129.7, 130.9, 131.4, 135.2 ($J_{C\text{-}F}$ = 8.5 Hz), 136.9, 137.8, 159.0 ($J_{C\text{-}F}$ = 255.3 Hz).

1.12. 5-Bromo-2-[(2-fluorophenyl)sulfonyl]isoindoline **2l**

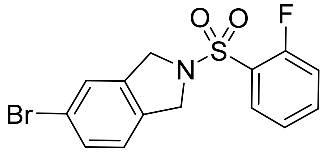

White solid, 189 mg (isolated yield 86%); UPLC/MS purity 100%, t_R = 8.04; $C_{14}H_{11}BrFNO_2S$, MW 356.21, Monoisotopic Mass 354.97, $[M+H]^+$ 356.0/358.1. ^1H NMR (500 MHz, $CDCl_3$) δ ppm 4.68 (s, 2H), 4.72 (s, 2H), 7.06 (d, J = 8.3 Hz, 1H), 7.14–7.21 (m, 1H), 7.28 (td, J = 7.7, 0.9 Hz, 1H), 7.33 (s, 1H), 7.35–7.39 (m, 1H), 7.52–7.58 (m, 1H), 7.92–7.96 (m, 1H). ^{13}C NMR (126 MHz, $CDCl_3$) δ ppm 53.1 (dd, $J_{C\text{-}F}$ = 6.0, 3.0 Hz), 117.5 ($J_{C\text{-}F}$ = 21.7 Hz), 121.7, 124.3, 124.6 ($J_{C\text{-}F}$ = 3.6 Hz), 126.0 ($J_{C\text{-}F}$ = 15.1 Hz), 126.1, 131.1, 131.4, 135.1, 135.2 ($J_{C\text{-}F}$ = 8.5 Hz), 138.3, 159.0 ($J_{C\text{-}F}$ = 255.3 Hz).

2. UPLC/MS, ^{1}H NMR and ^{13}C NMR spectra of all intermediates and final compounds

2.1. 2-[(4-Fluorophenyl)sulfonyl]isoindoline **2a**

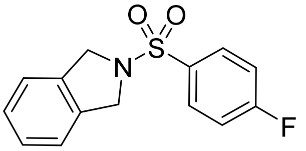

UPLC/MS

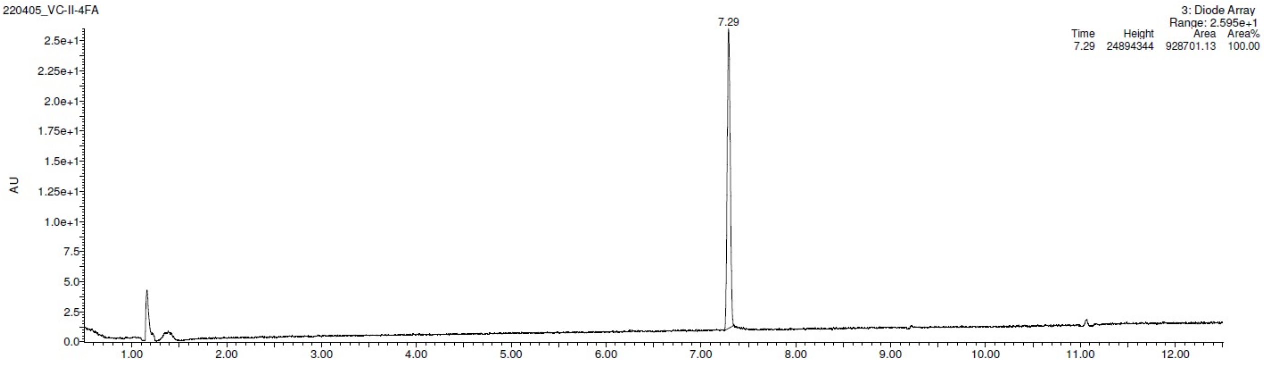

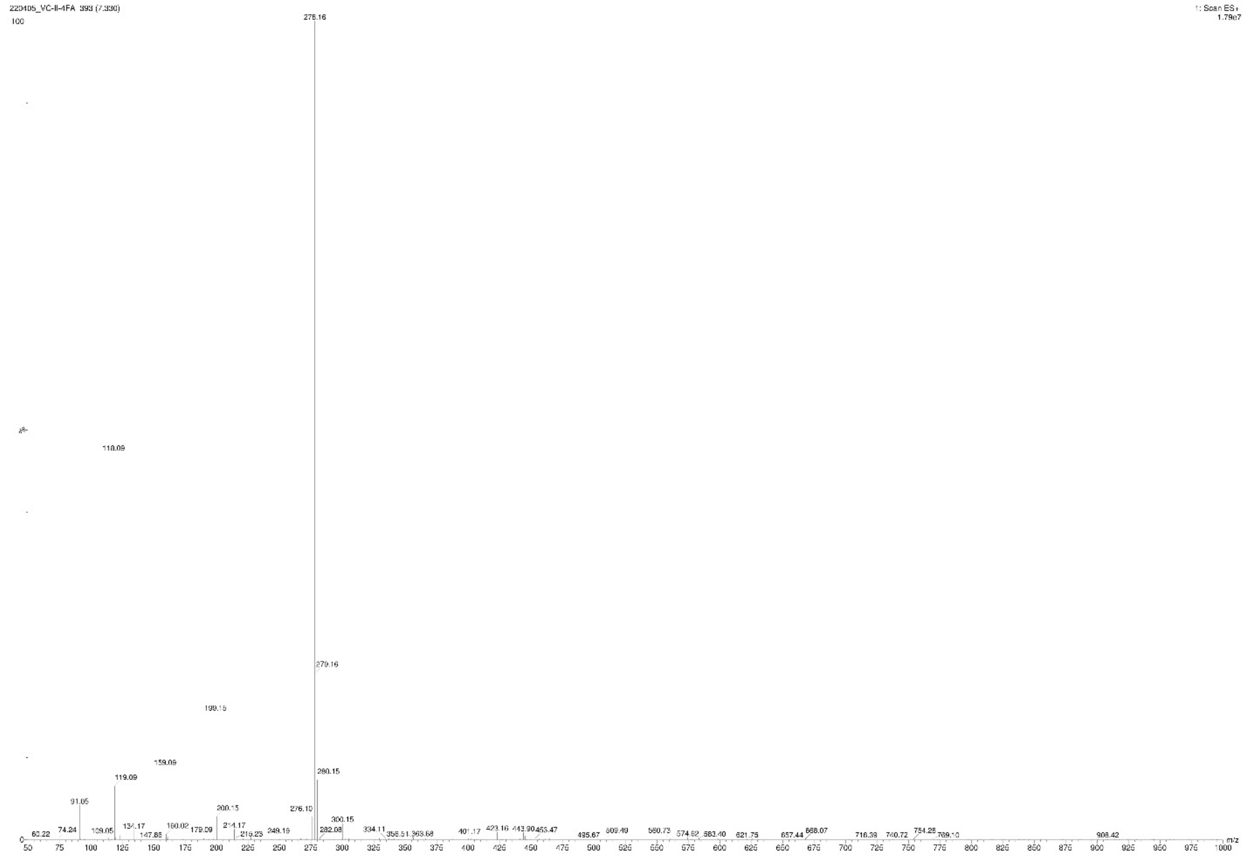

^{1}H-NMR (500 MHz, CDCl$_3$)

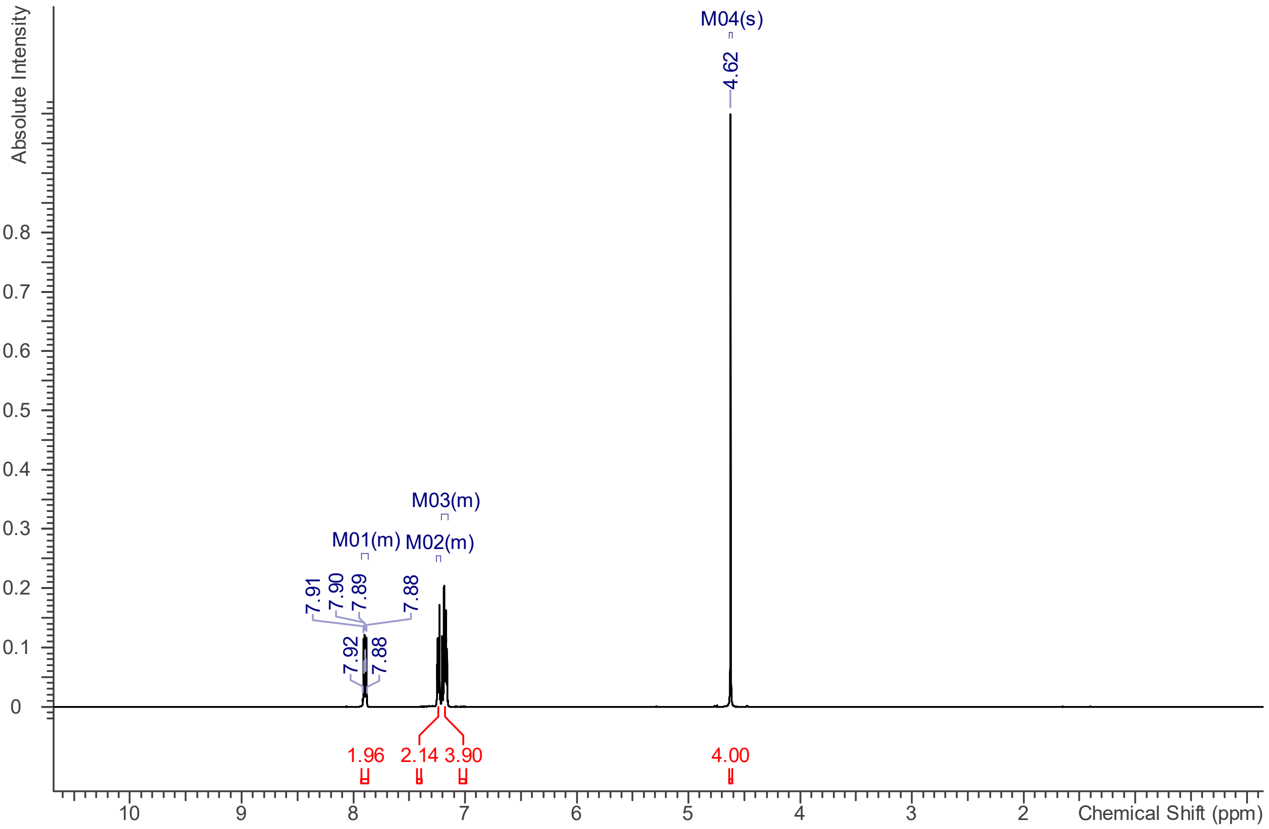

^{13}C-NMR (126 MHz, CDCl$_3$)

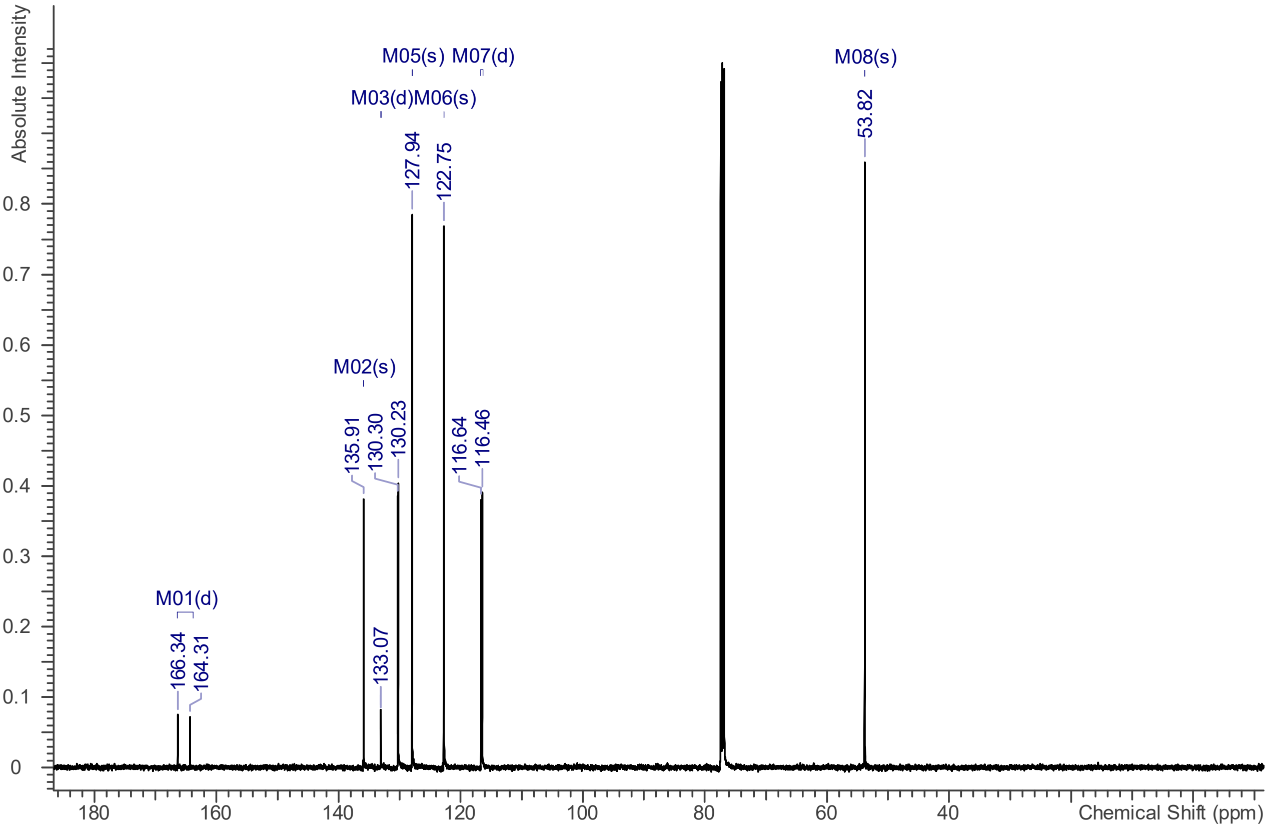

2.2. 4-Chloro-2-[(4-fluorophenyl)sulfonyl]isoindoline **2b**

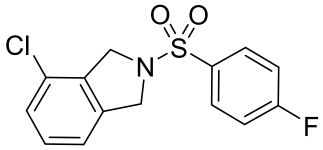

UPLC/MS

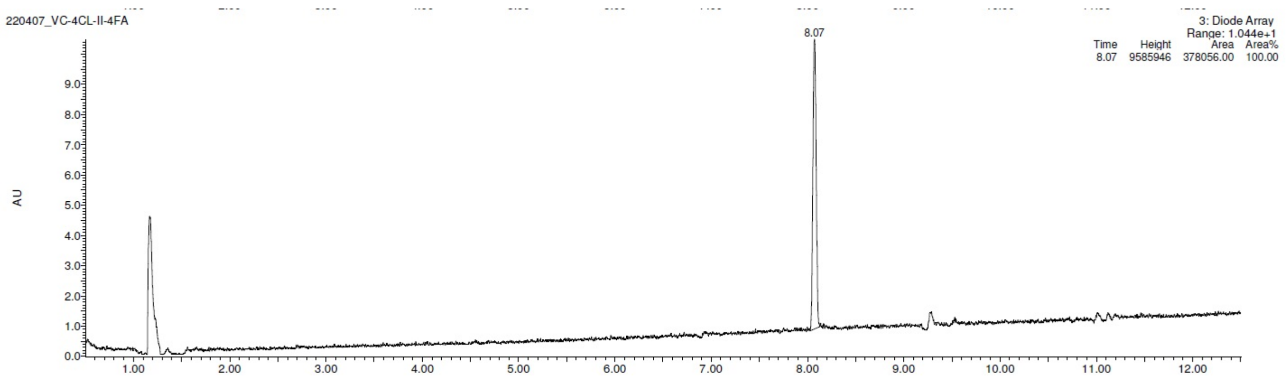

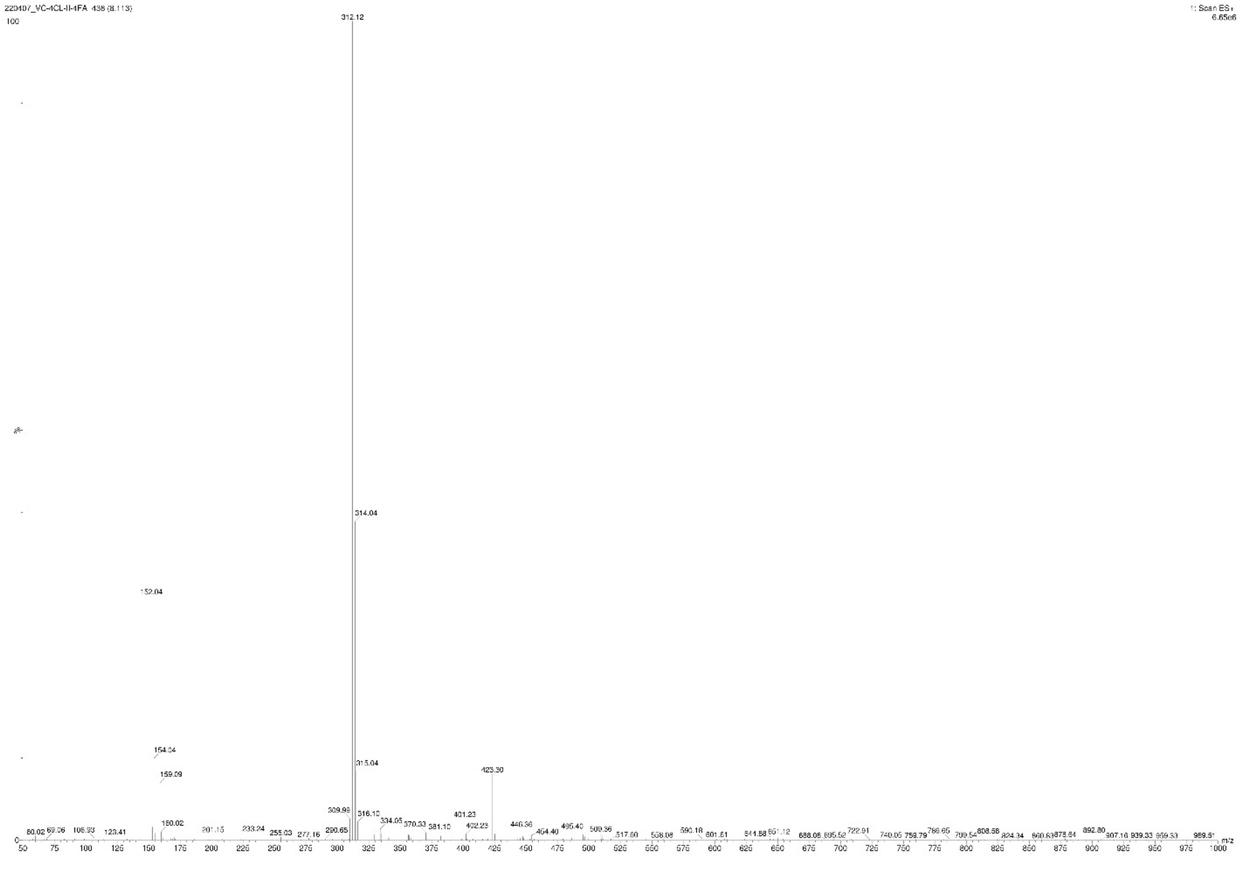

^{1}H-NMR (500 MHz, $CDCl_3$)

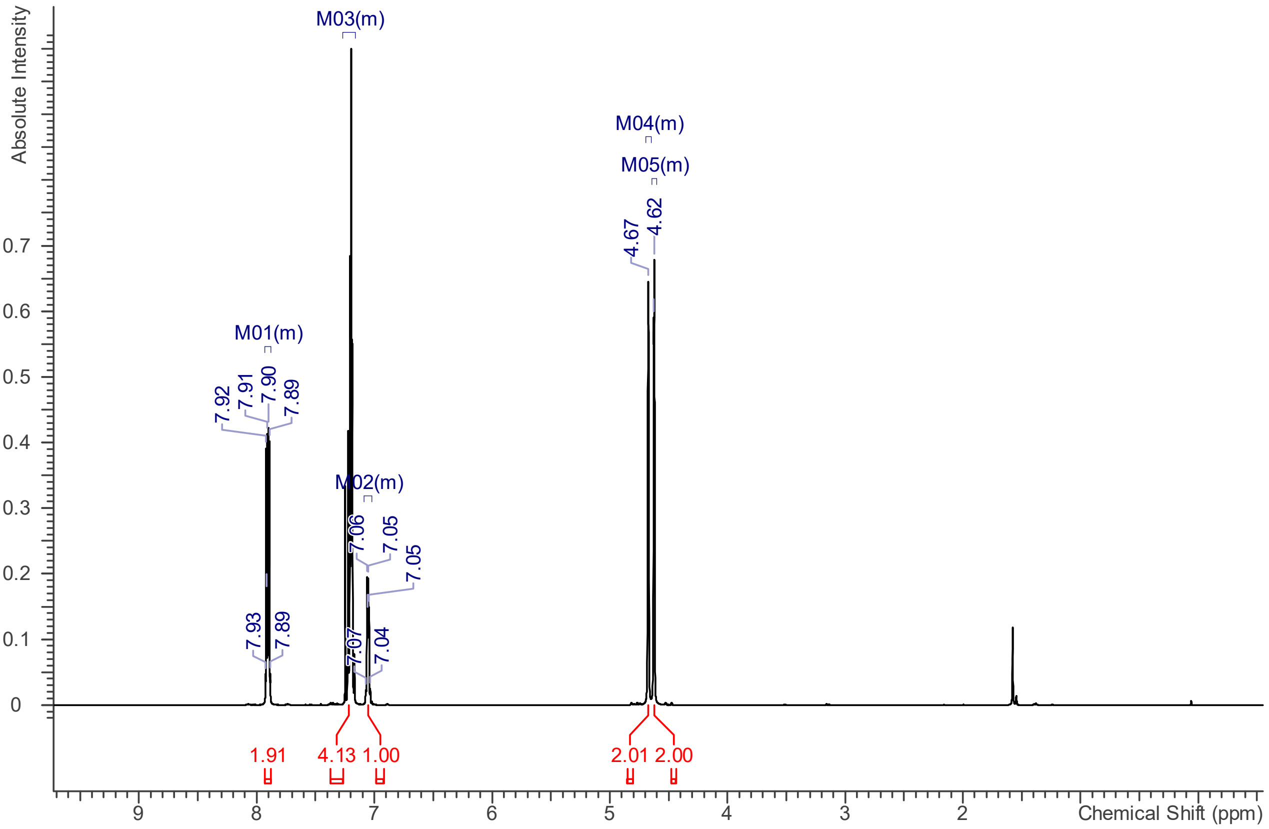

^{13}C-NMR (126 MHz, $CDCl_3$)

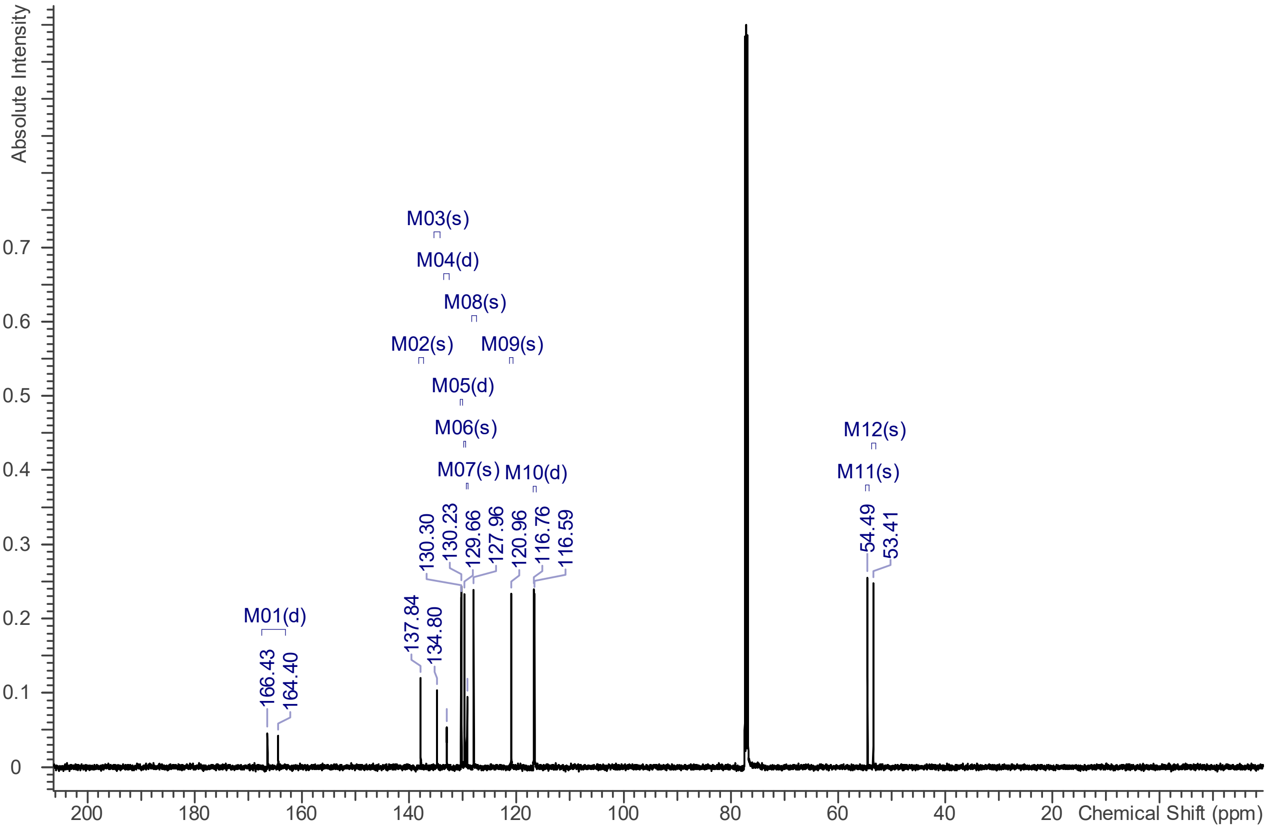

2.3. 4-Bromo-2-[(4-fluorophenyl)sulfonyl]isoindoline **2c**

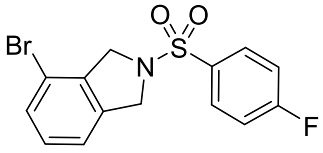

UPLC/MS

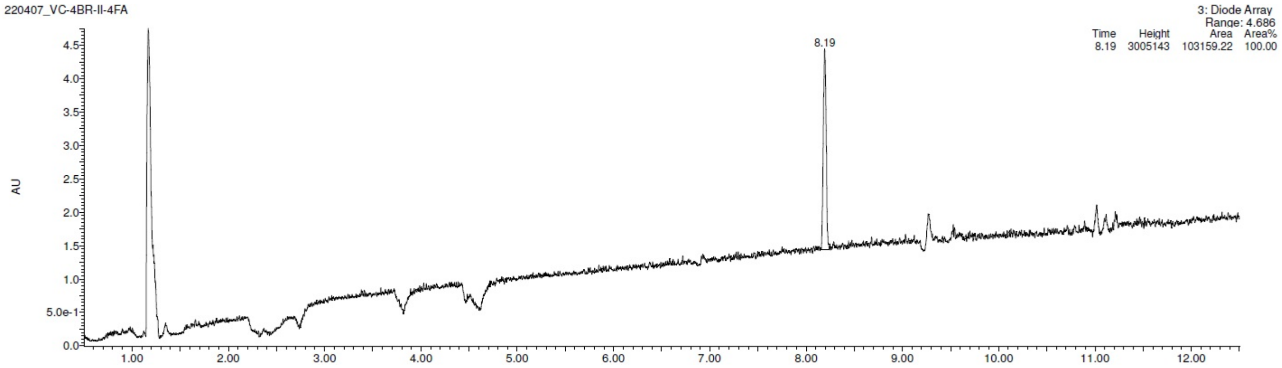

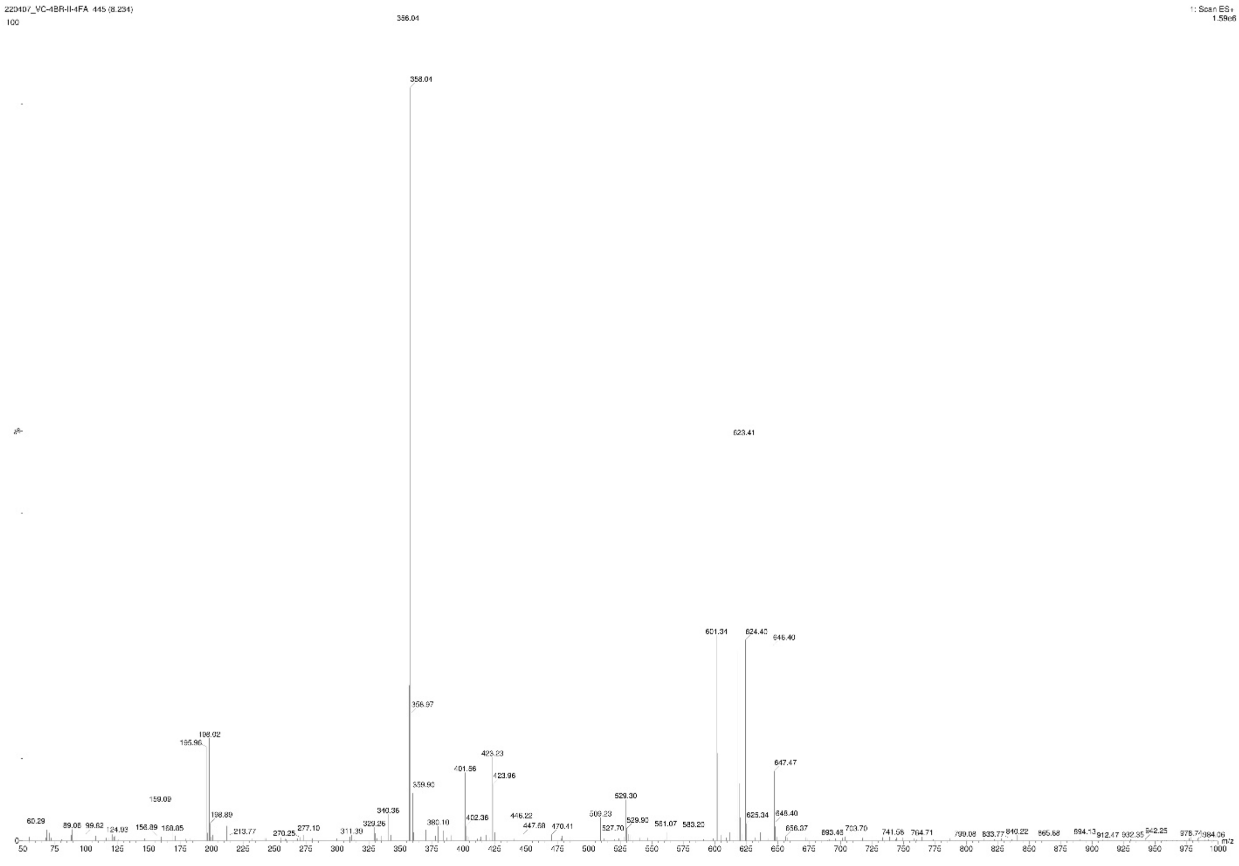

^{1}H-NMR (500 MHz, $CDCl_3$)

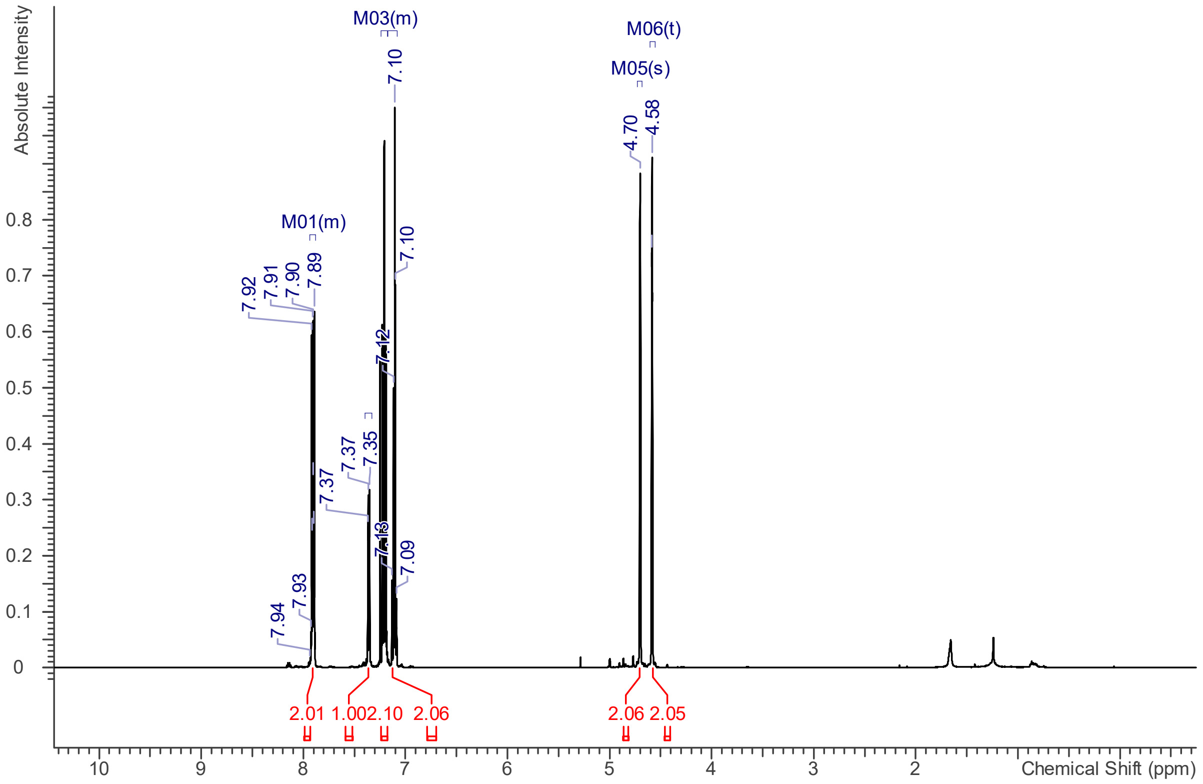

^{13}C-NMR (126 MHz, $CDCl_3$)

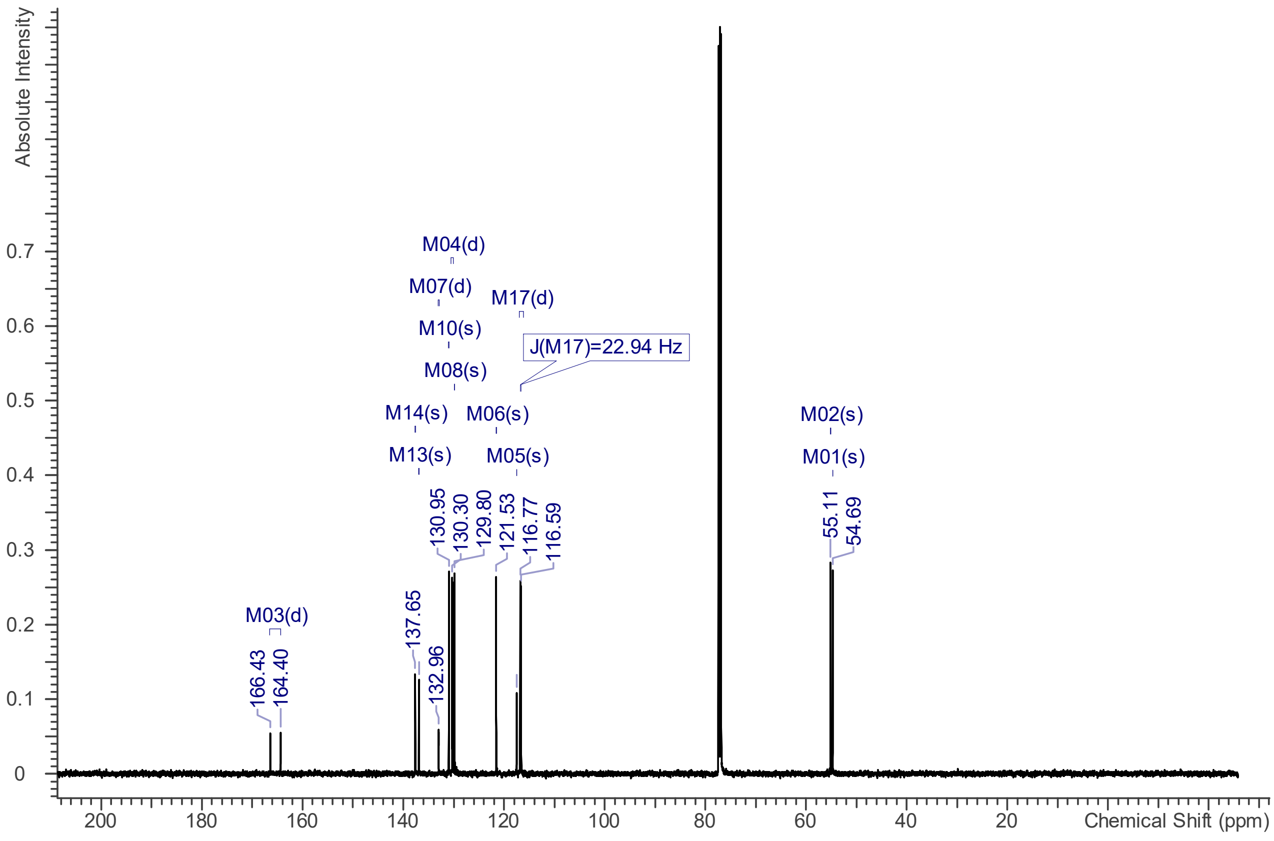

2.4. 5-Bromo-2-[(4-fluorophenyl)sulfonyl]isoindoline **2d**

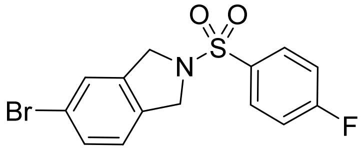

UPLC/MS

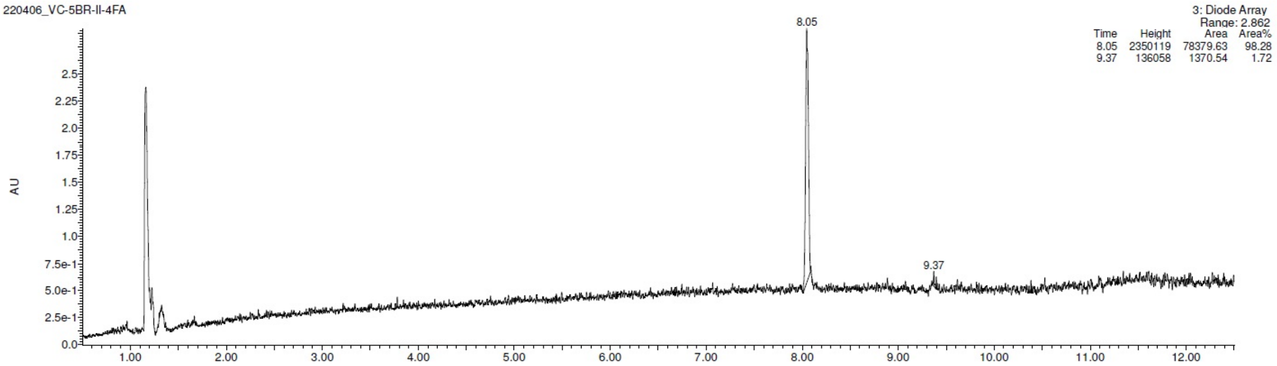

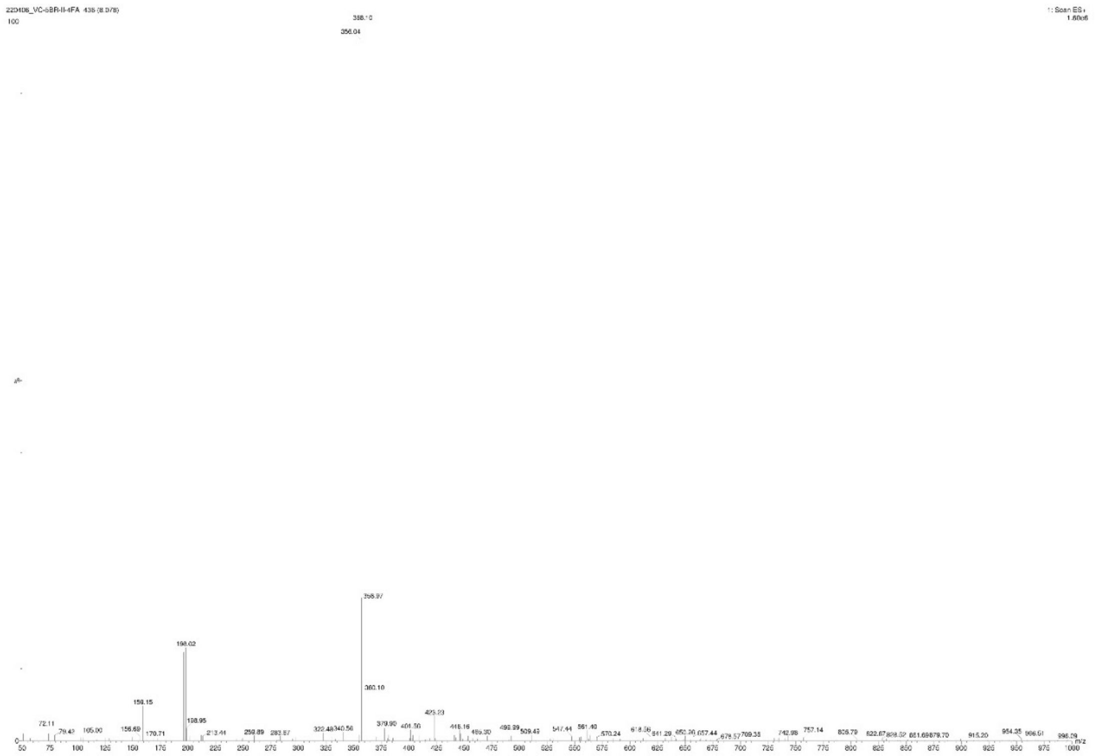

^{1}H-NMR (500 MHz, $CDCl_3$)

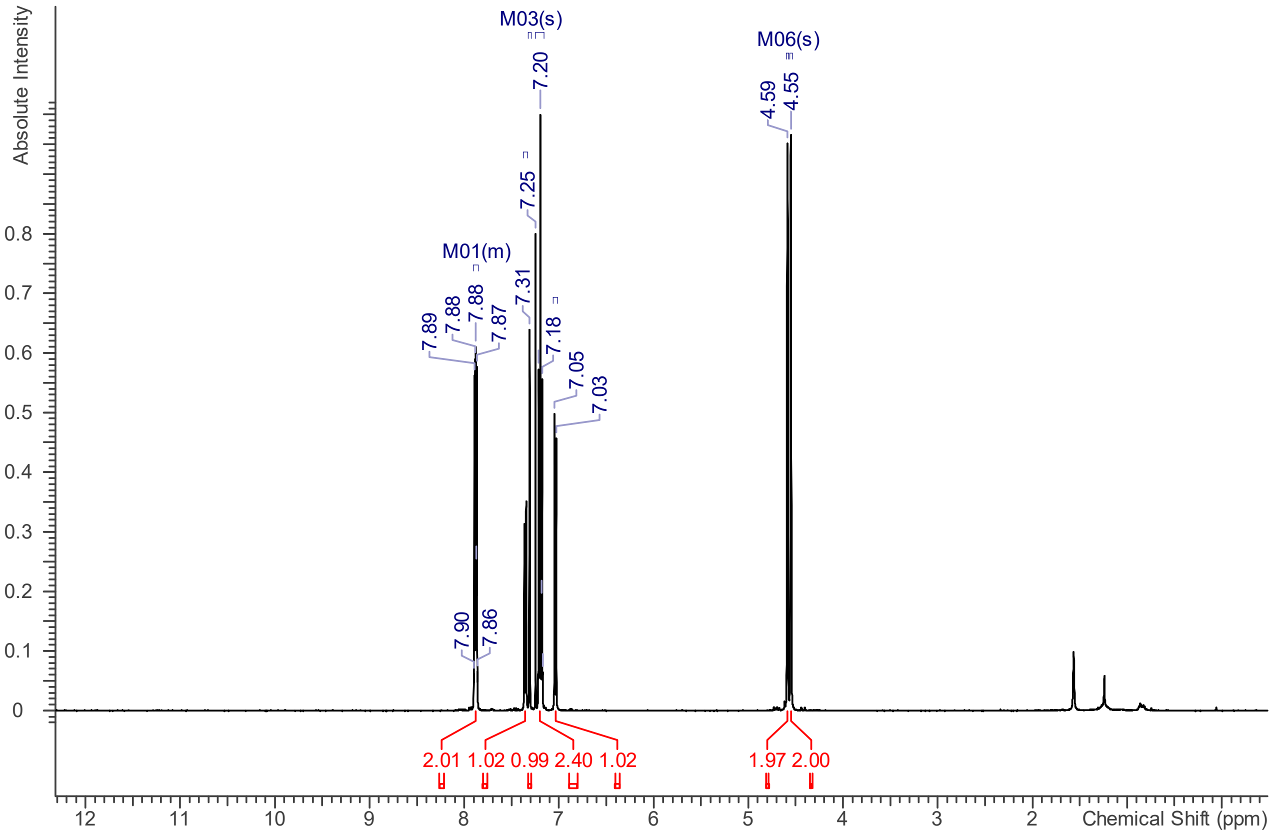

^{13}C-NMR (126 MHz, $CDCl_3$)

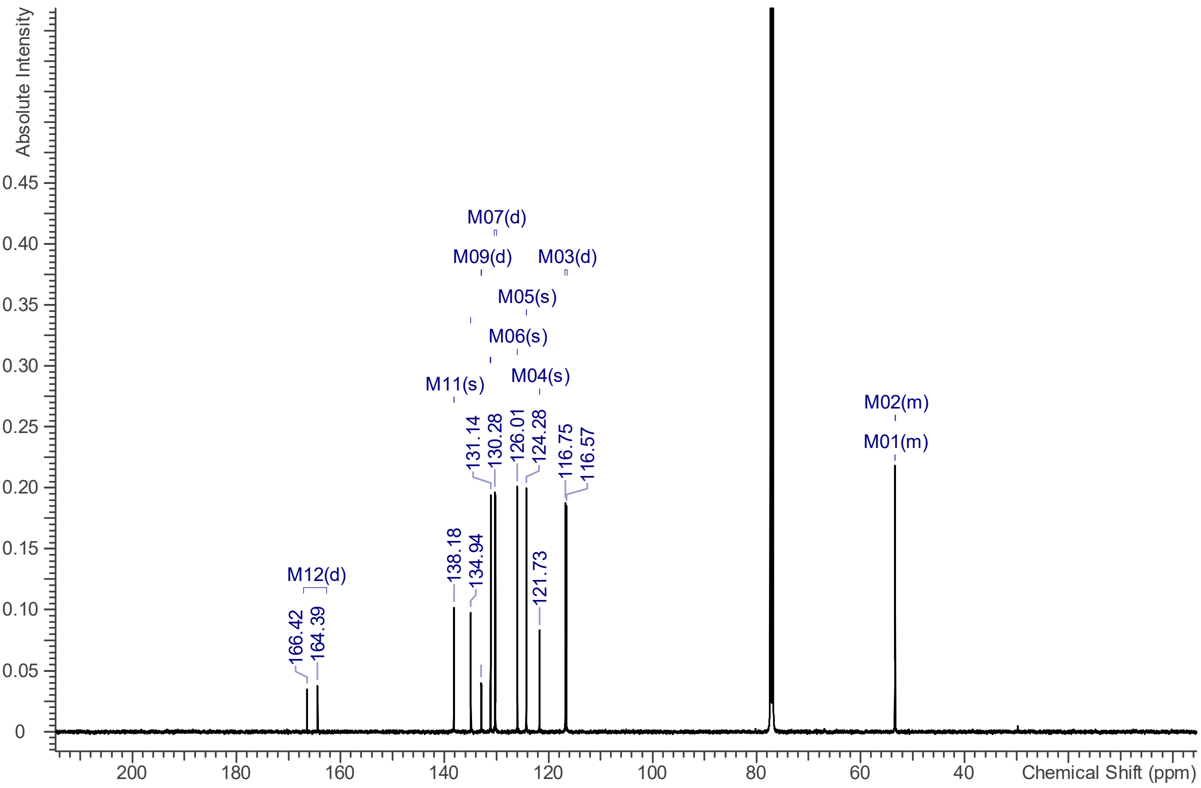

2.5. 2-[(3-Fluorophenyl)sulfonyl]isoindoline **2e**

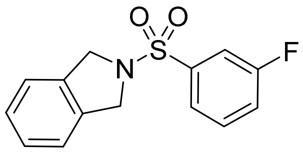

UPLC/MS

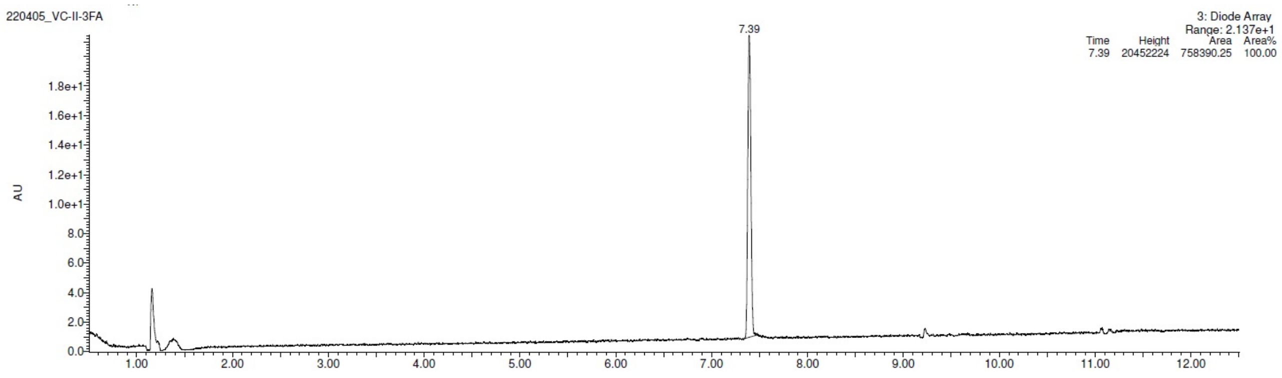

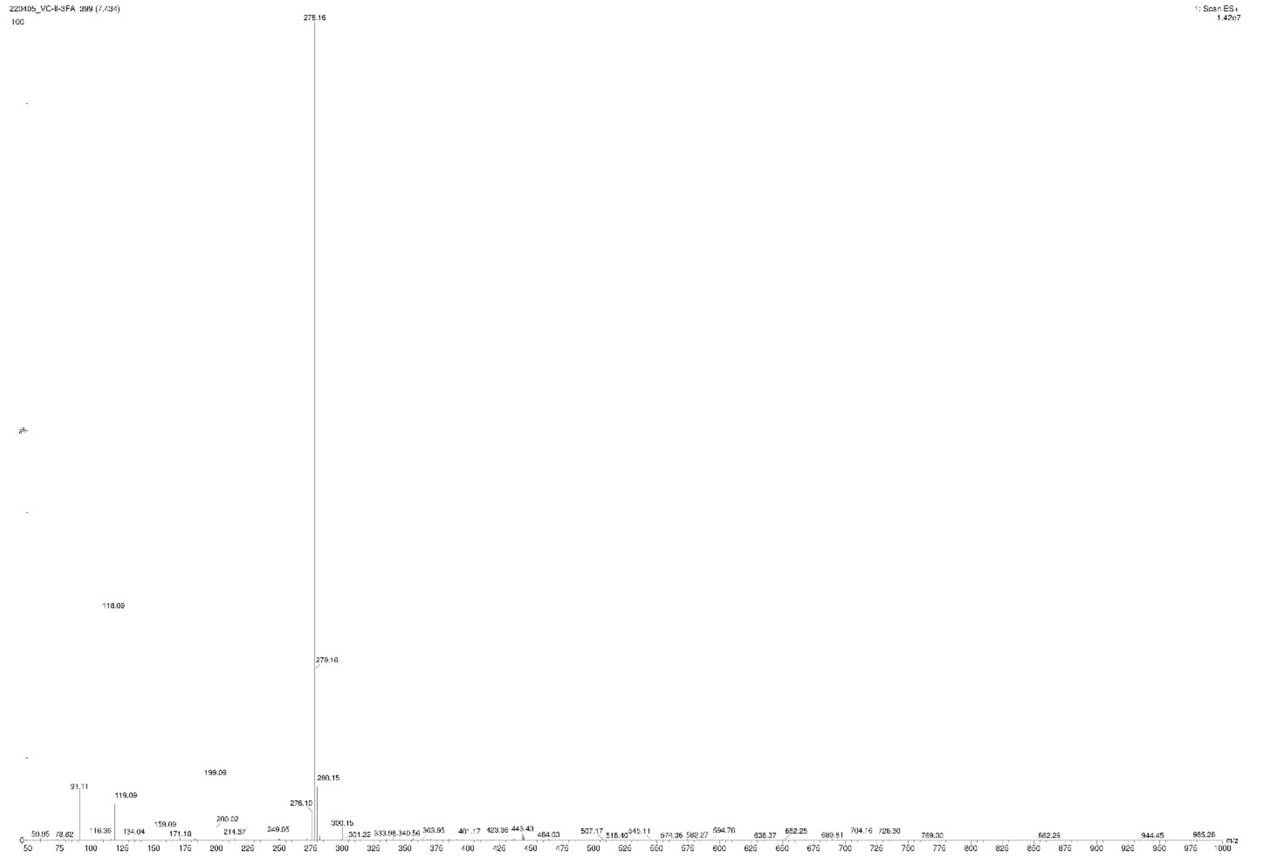

^{1}H-NMR (500 MHz, $CDCl_3$)

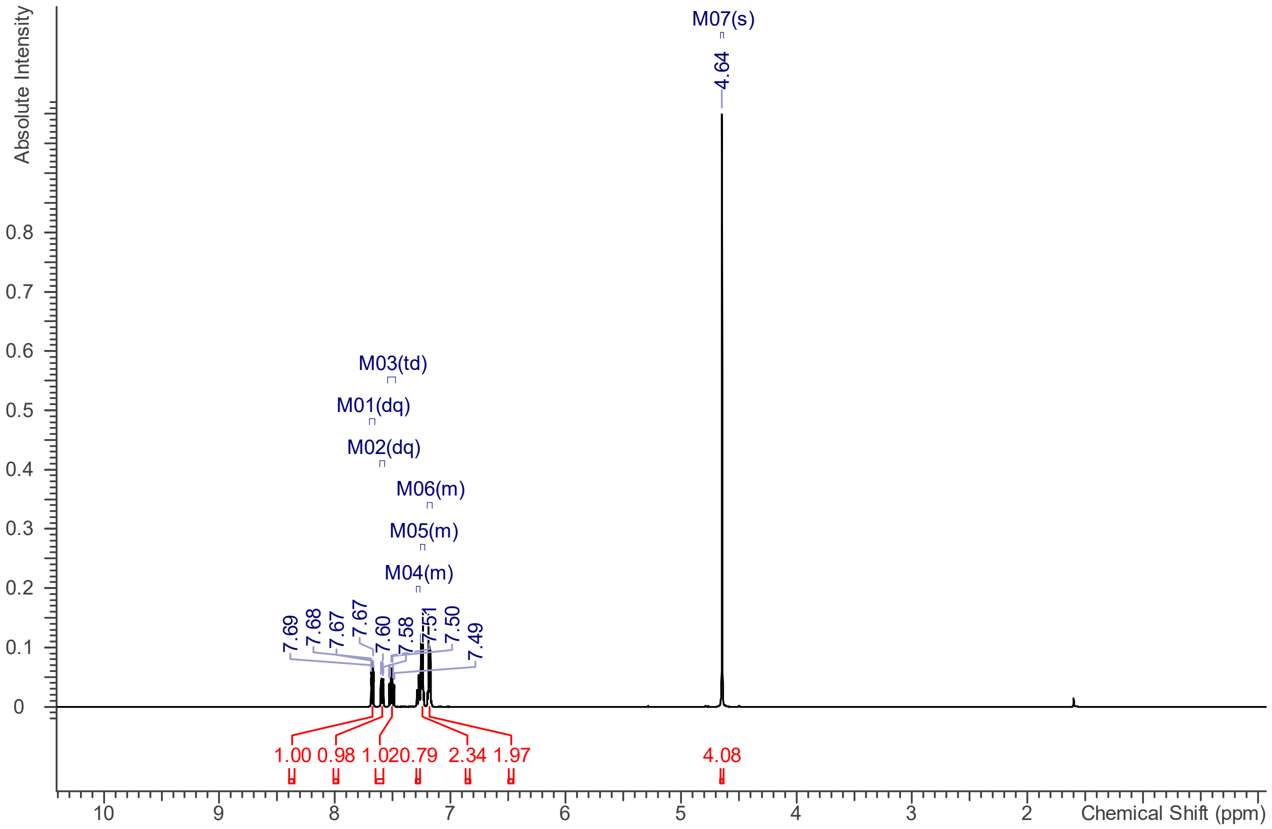

^{13}C-NMR (126 MHz, $CDCl_3$)

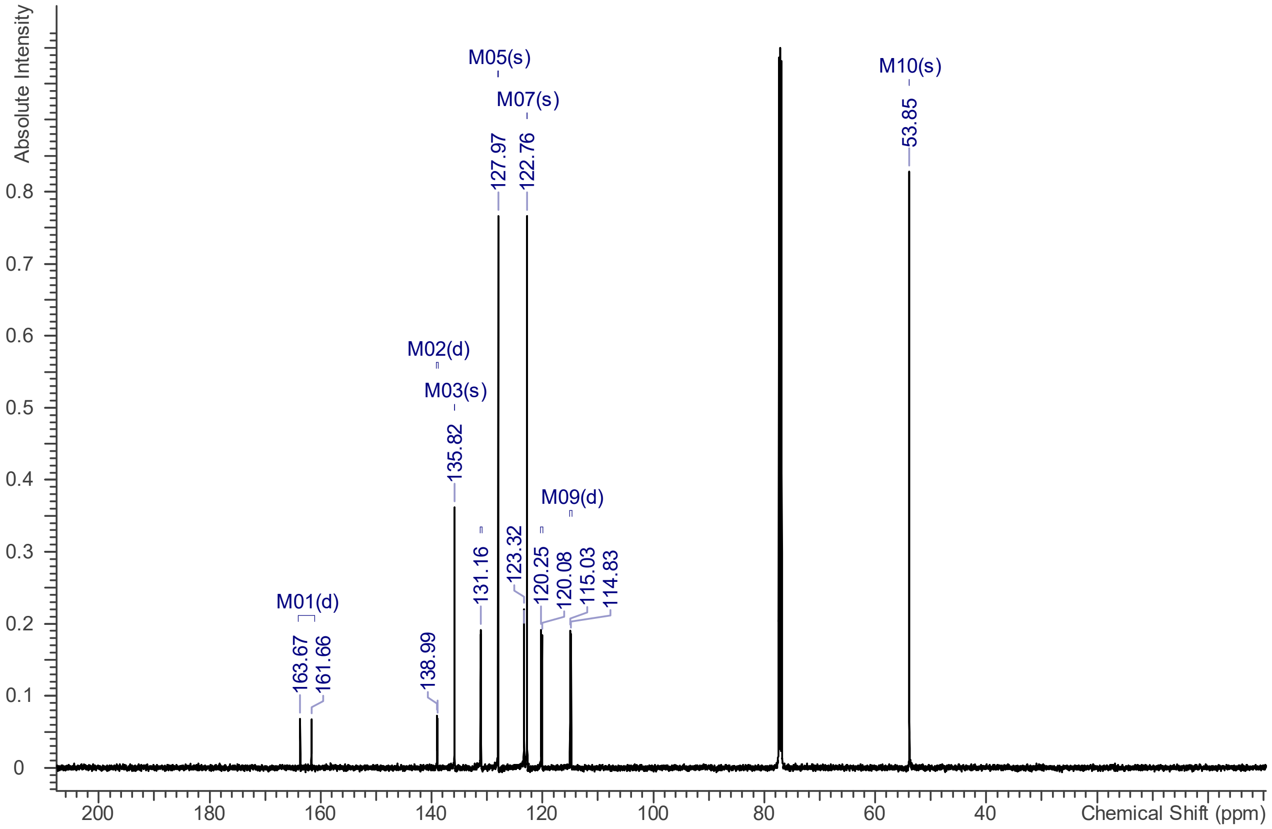

2.6. 4-Chloro-2-[(3-fluorophenyl)sulfonyl]isoindoline **2f**

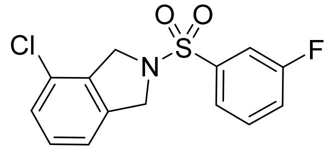

UPLC/MS

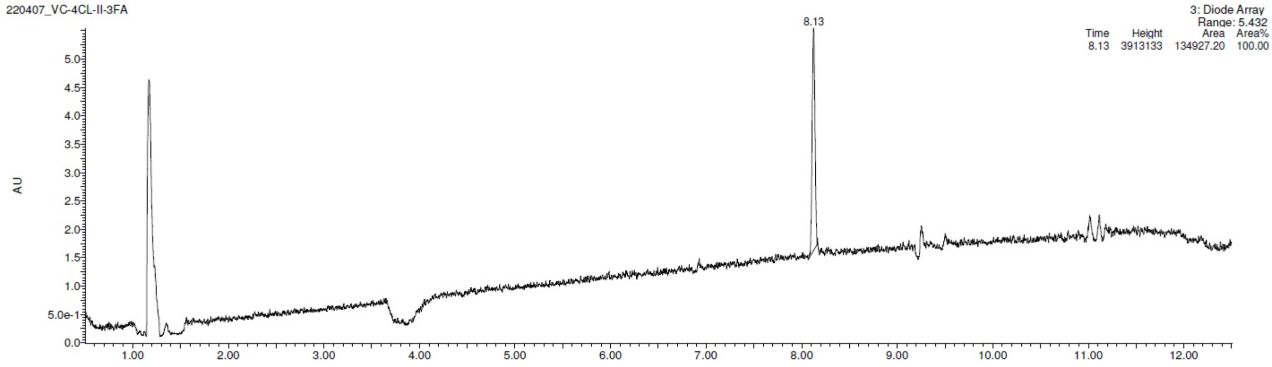

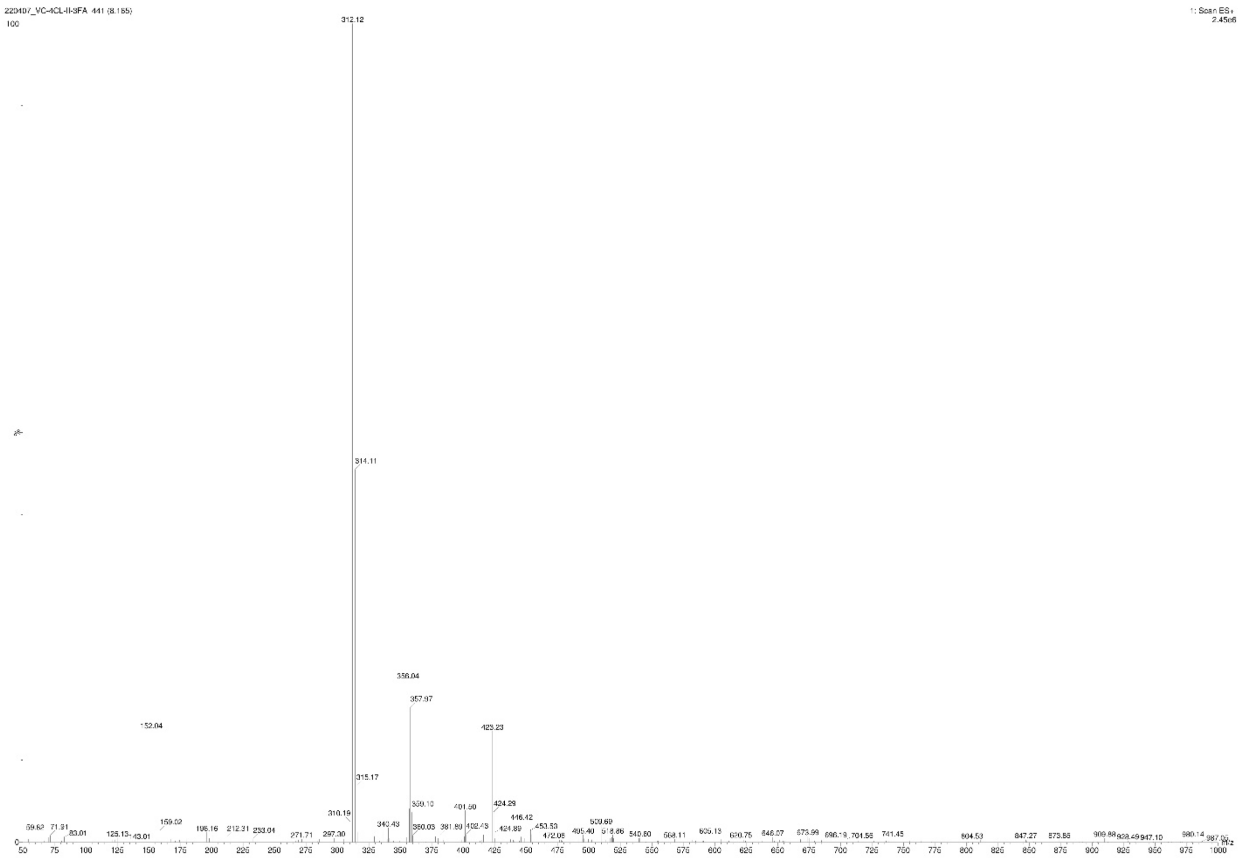

^{1}H-NMR (500 MHz, $CDCl_3$)

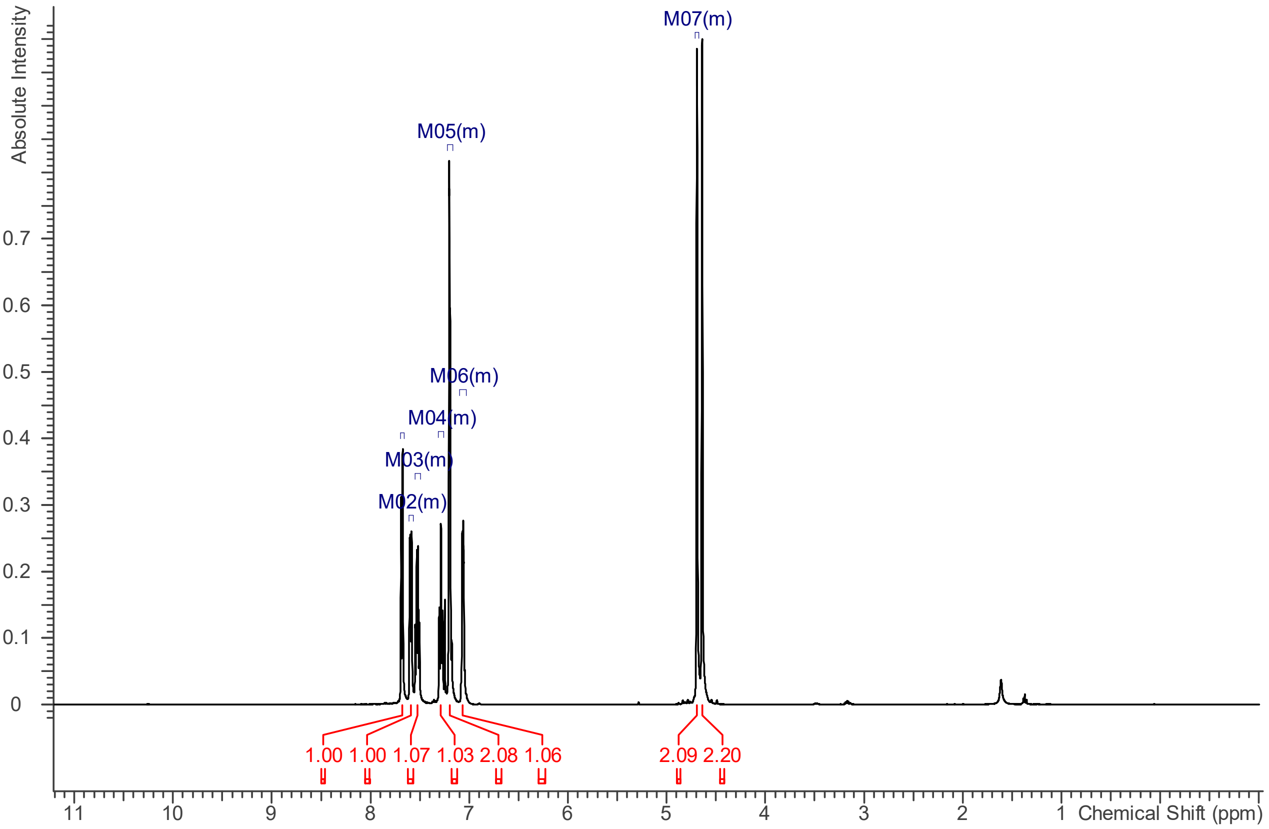

^{13}C-NMR (126 MHz, $CDCl_3$)

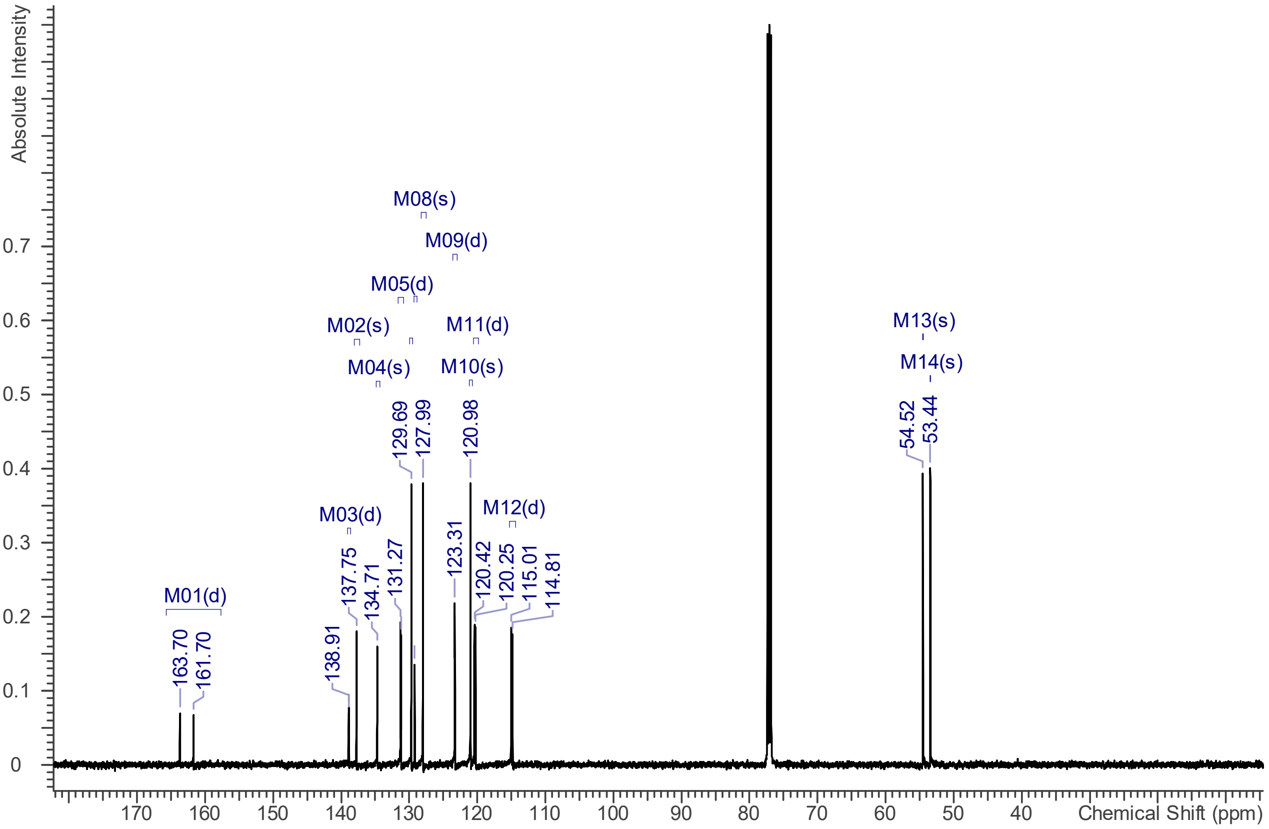

2.7. 4-Bromo-2-[(3-fluorophenyl)sulfonyl]isoindoline **2g**

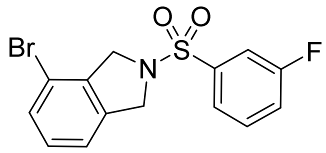

UPLC/MS

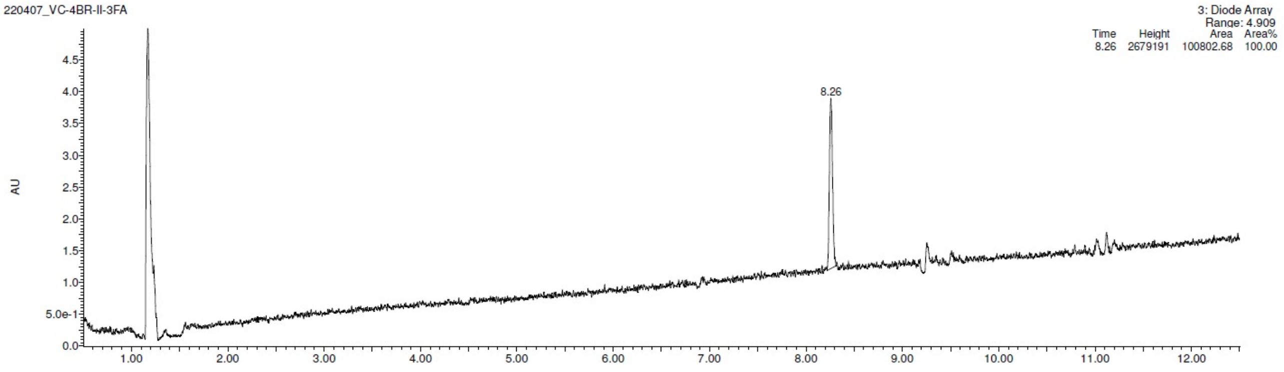

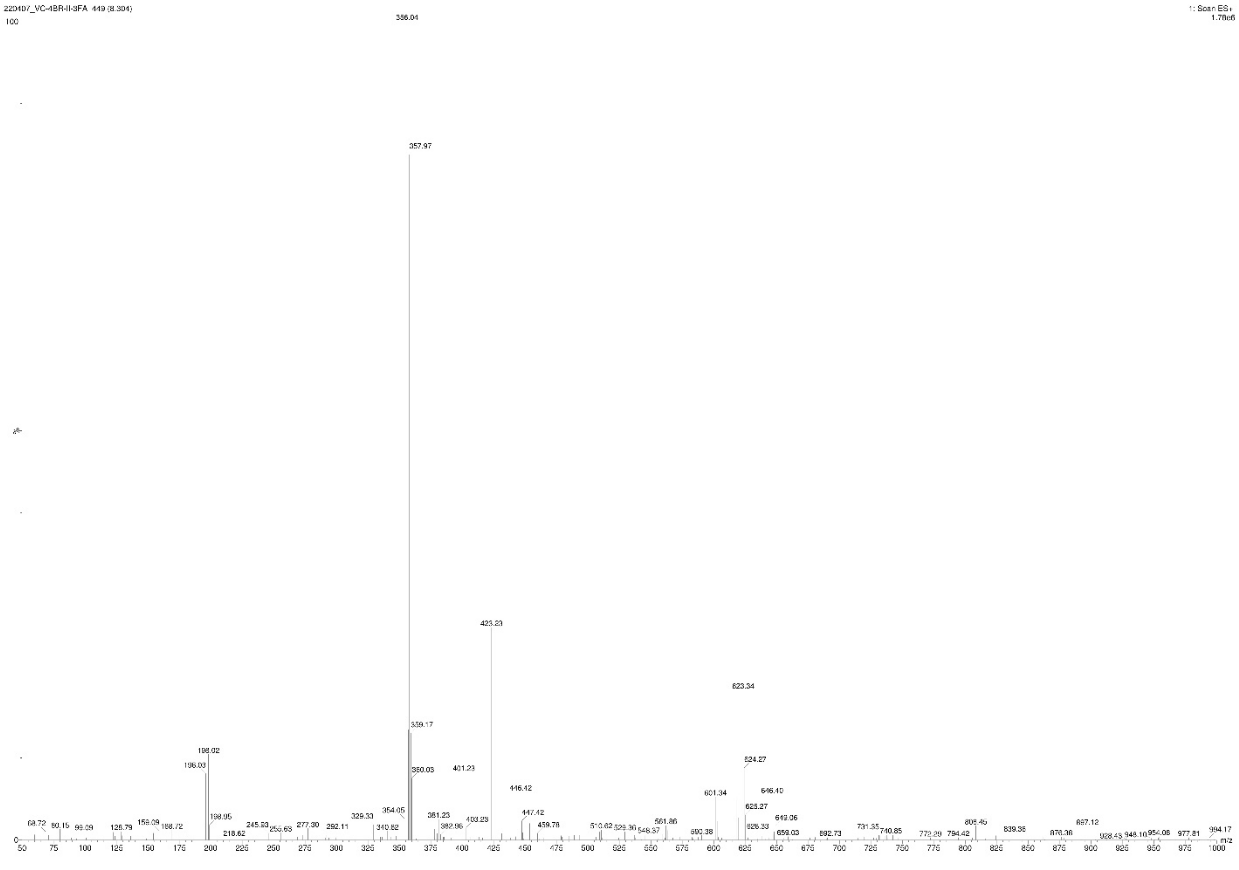

^{1}H-NMR (500 MHz, CDCl$_3$)

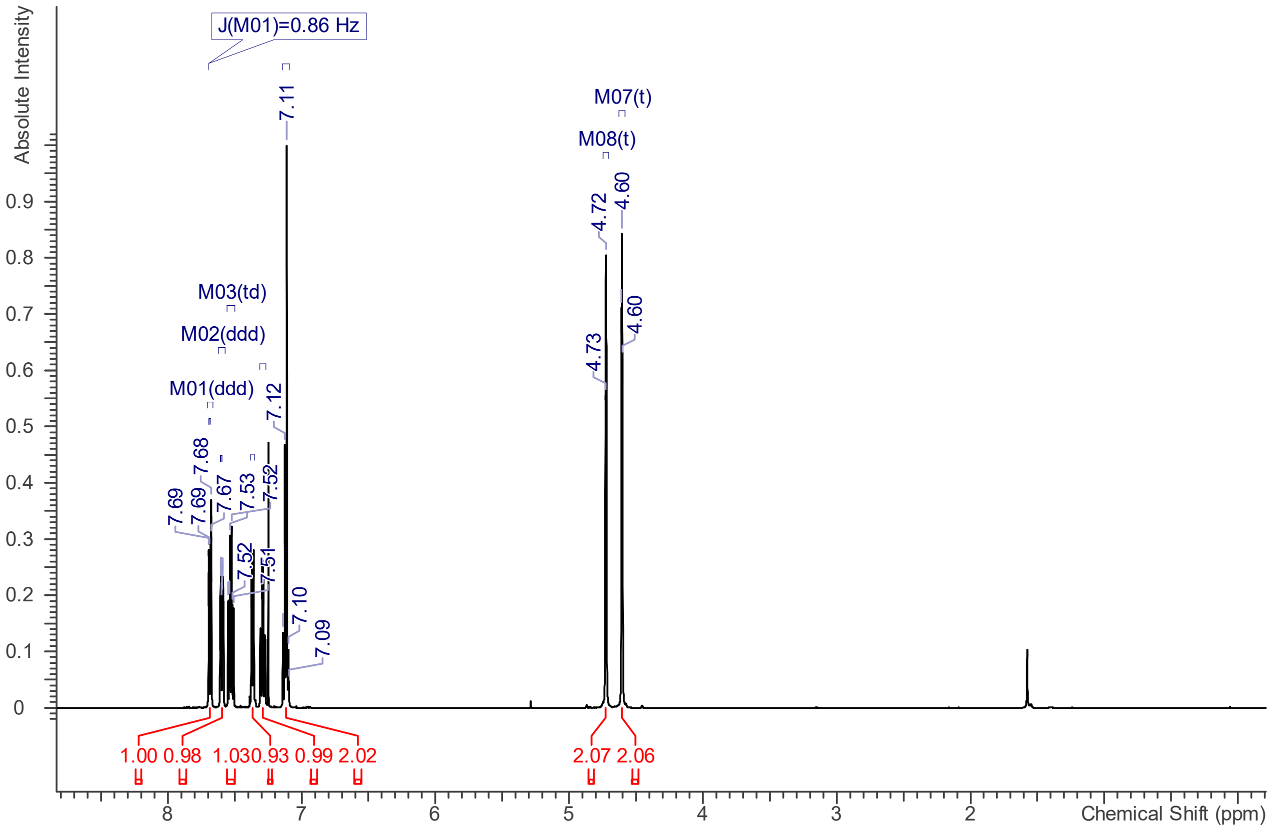

^{13}C-NMR (126 MHz, CDCl$_3$)

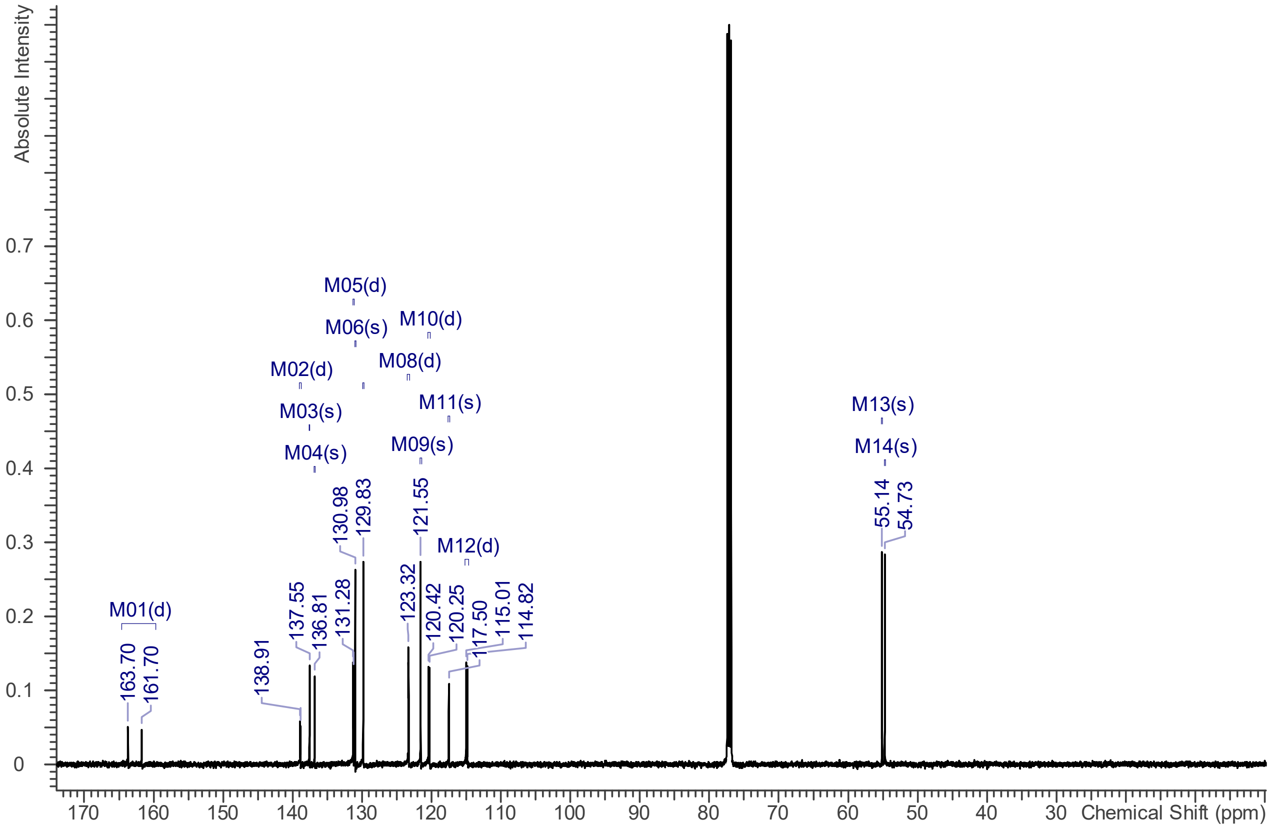

2.8. 5-Bromo-2-[(3-fluorophenyl)sulfonyl]isoindoline **2h**

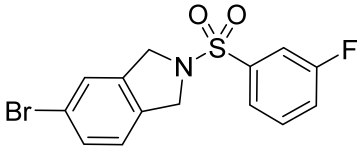

UPLC/MS

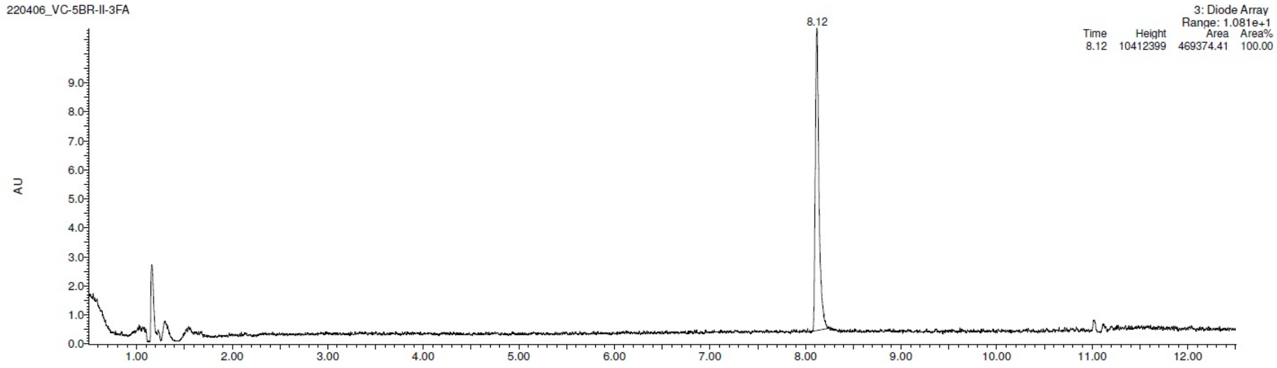

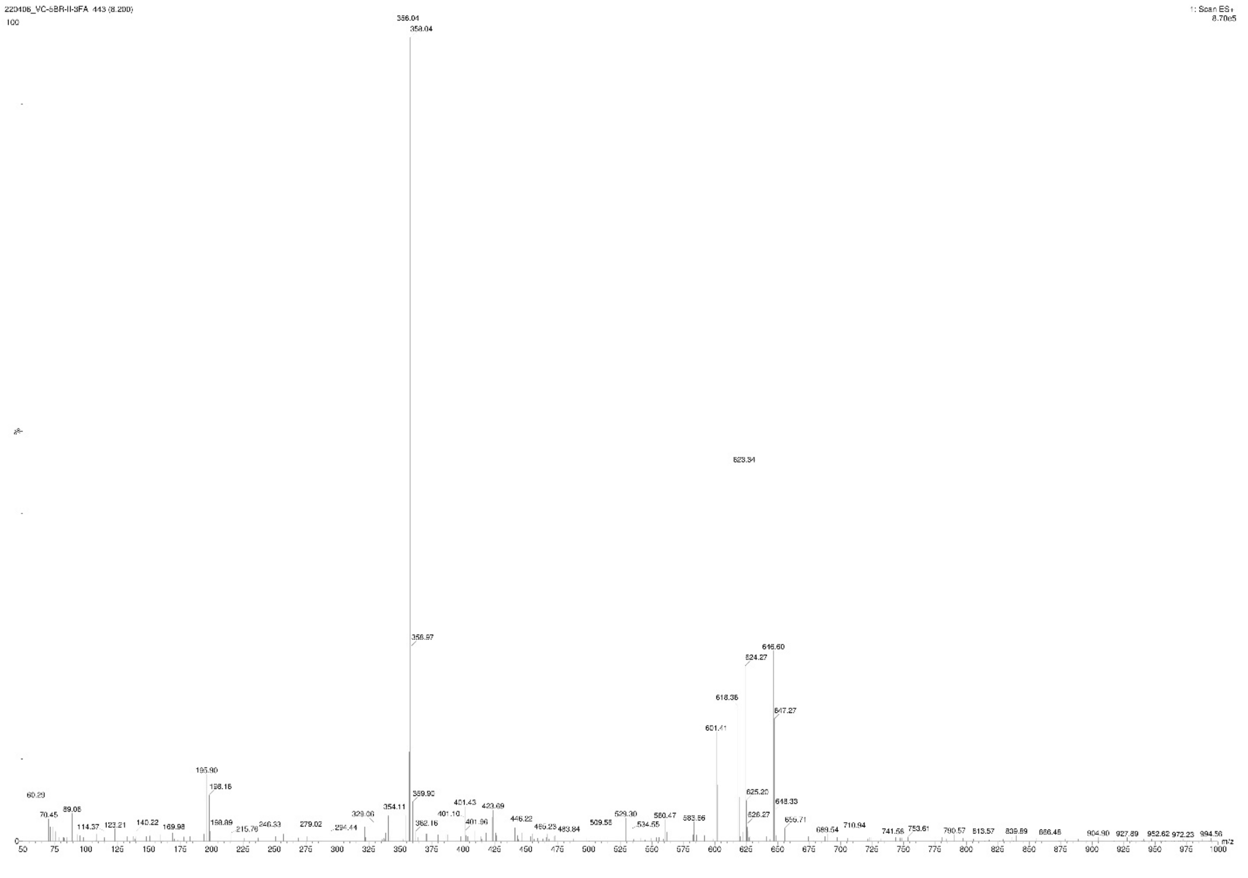

^{1}H-NMR (500 MHz, $CDCl_3$)

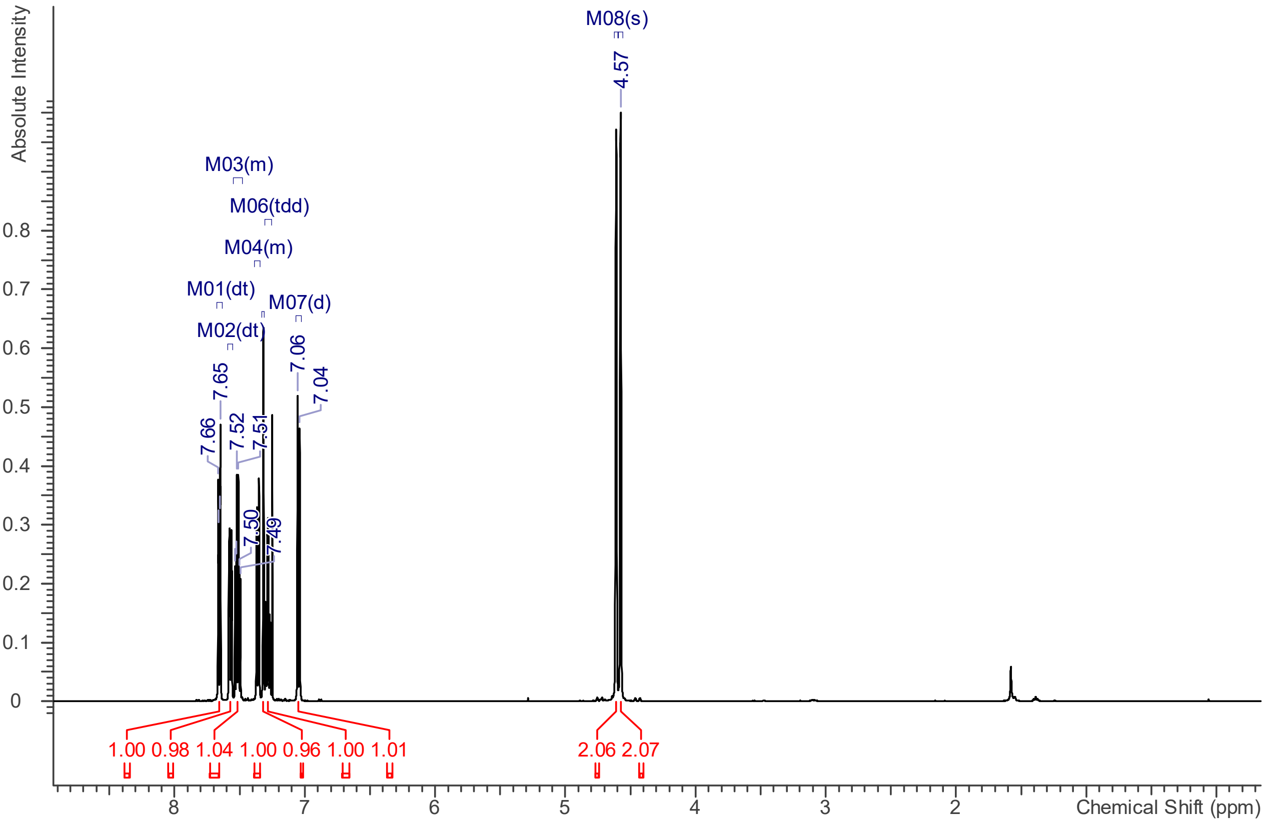

^{13}C-NMR (126 MHz, $CDCl_3$)

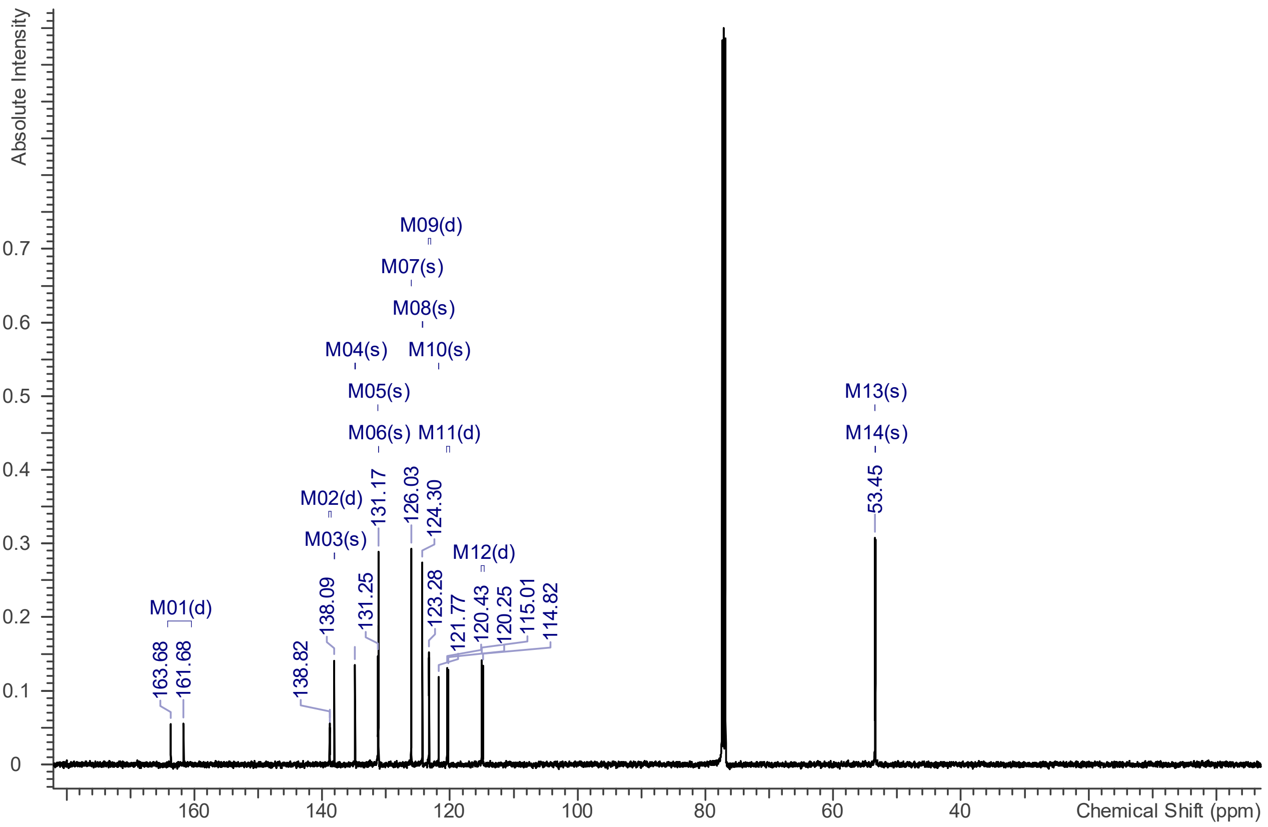

2.9. 2-[(2-Fluorophenyl)sulfonyl]isoindoline **2i**

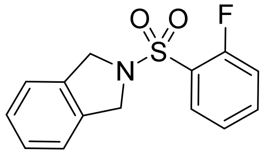

UPLC/MS

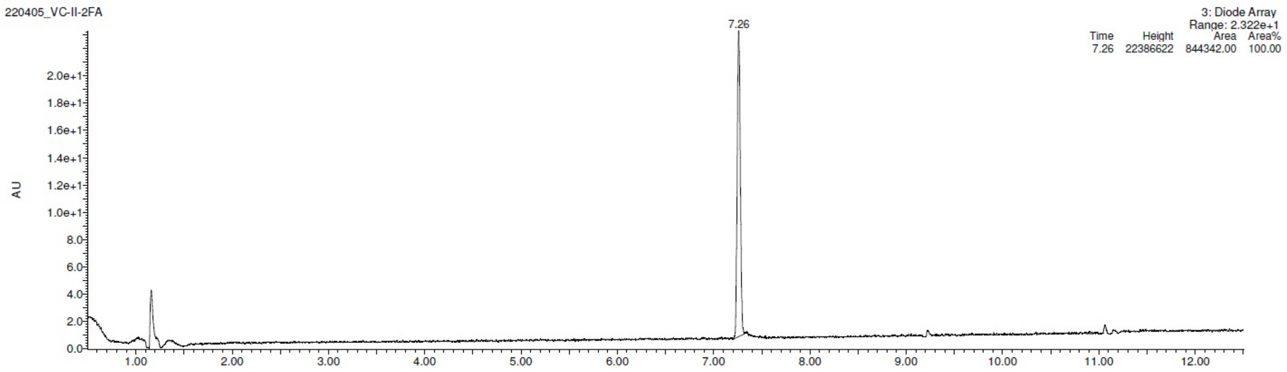

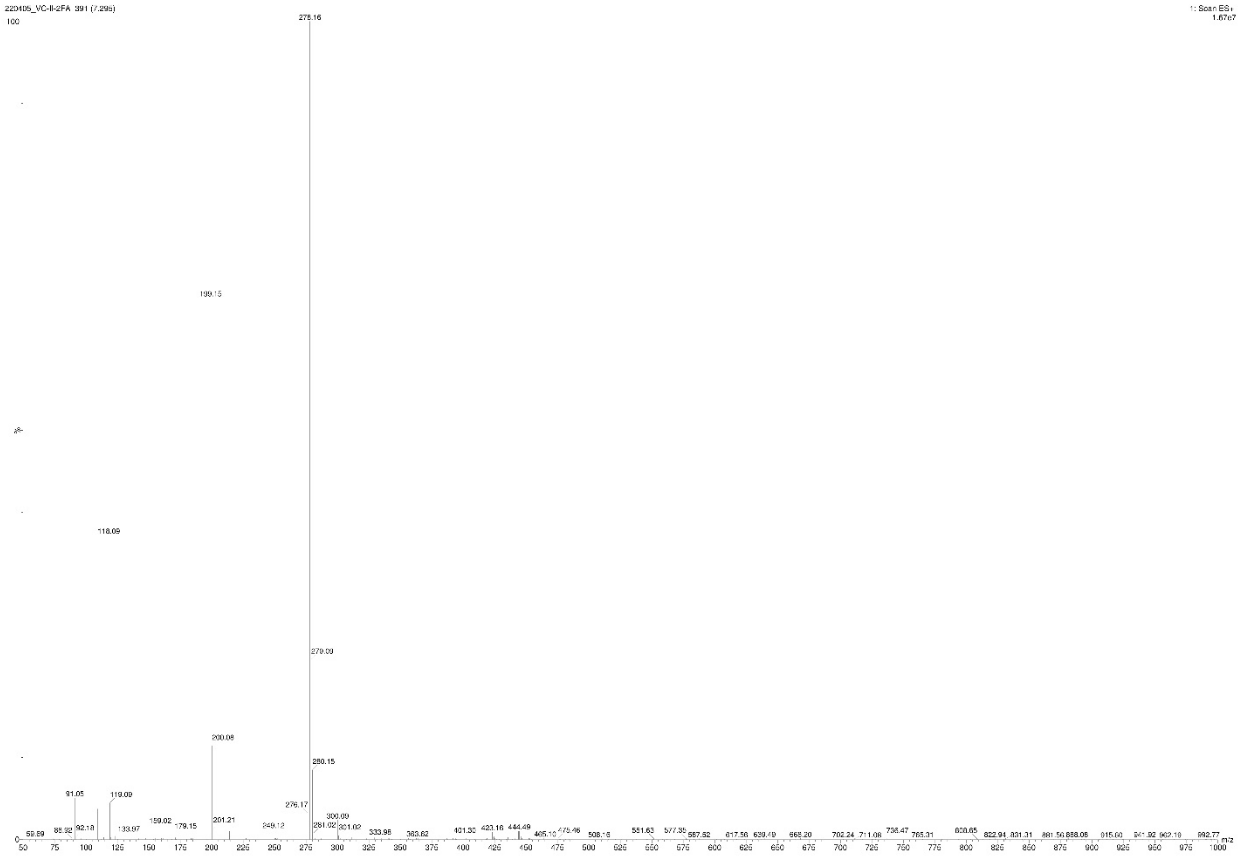

^{1}H-NMR (500 MHz, $CDCl_3$)

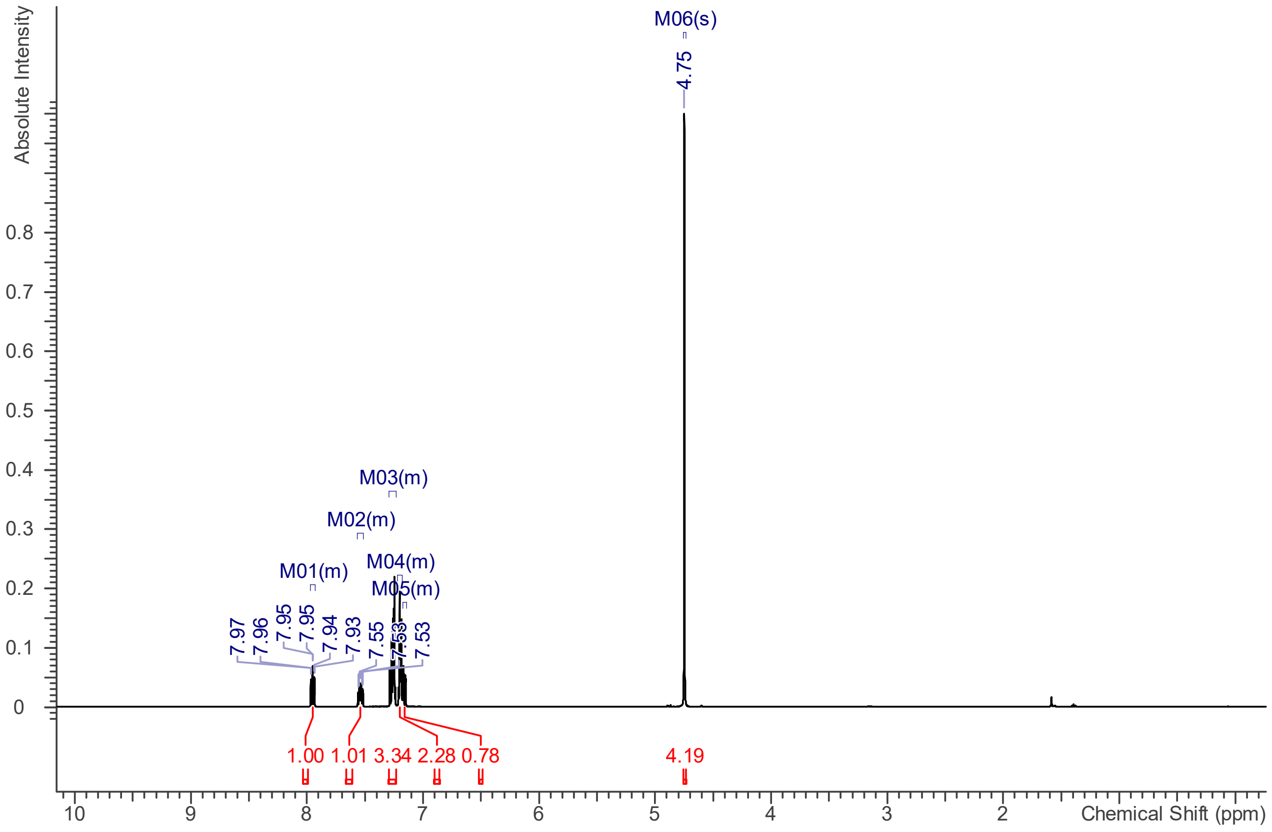

^{13}C-NMR (126 MHz, $CDCl_3$)

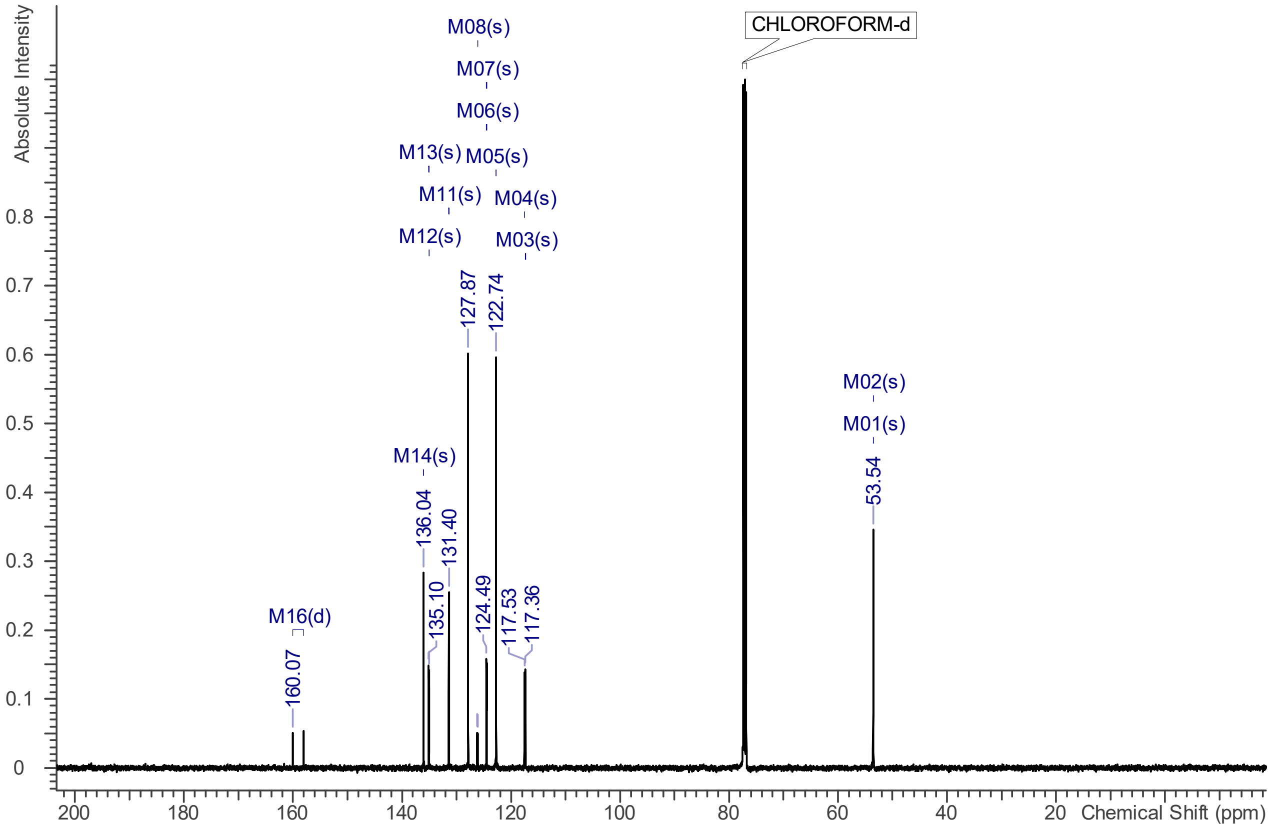

2.10. 4-Chloro-2-[(2-fluorophenyl)sulfonyl]isoindoline **2j**

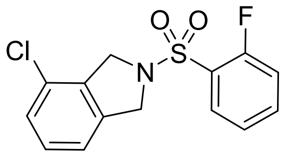

UPLC/MS

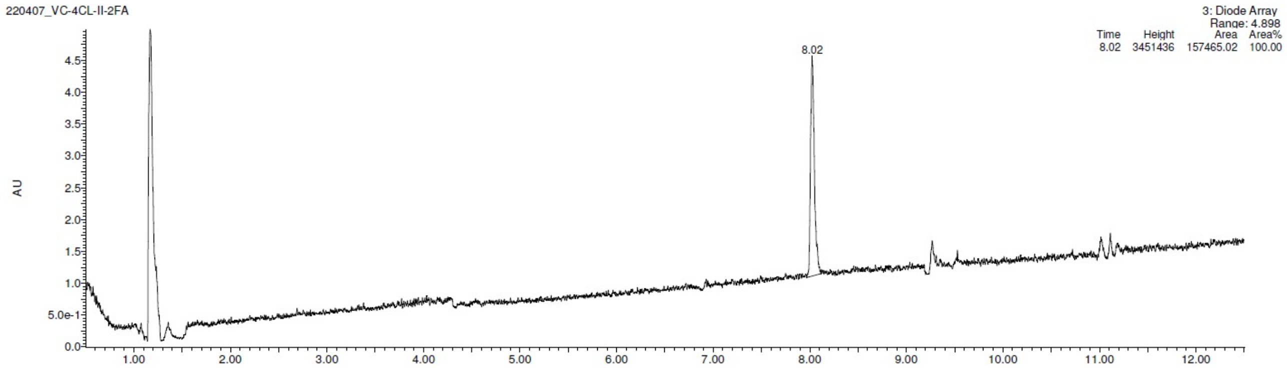

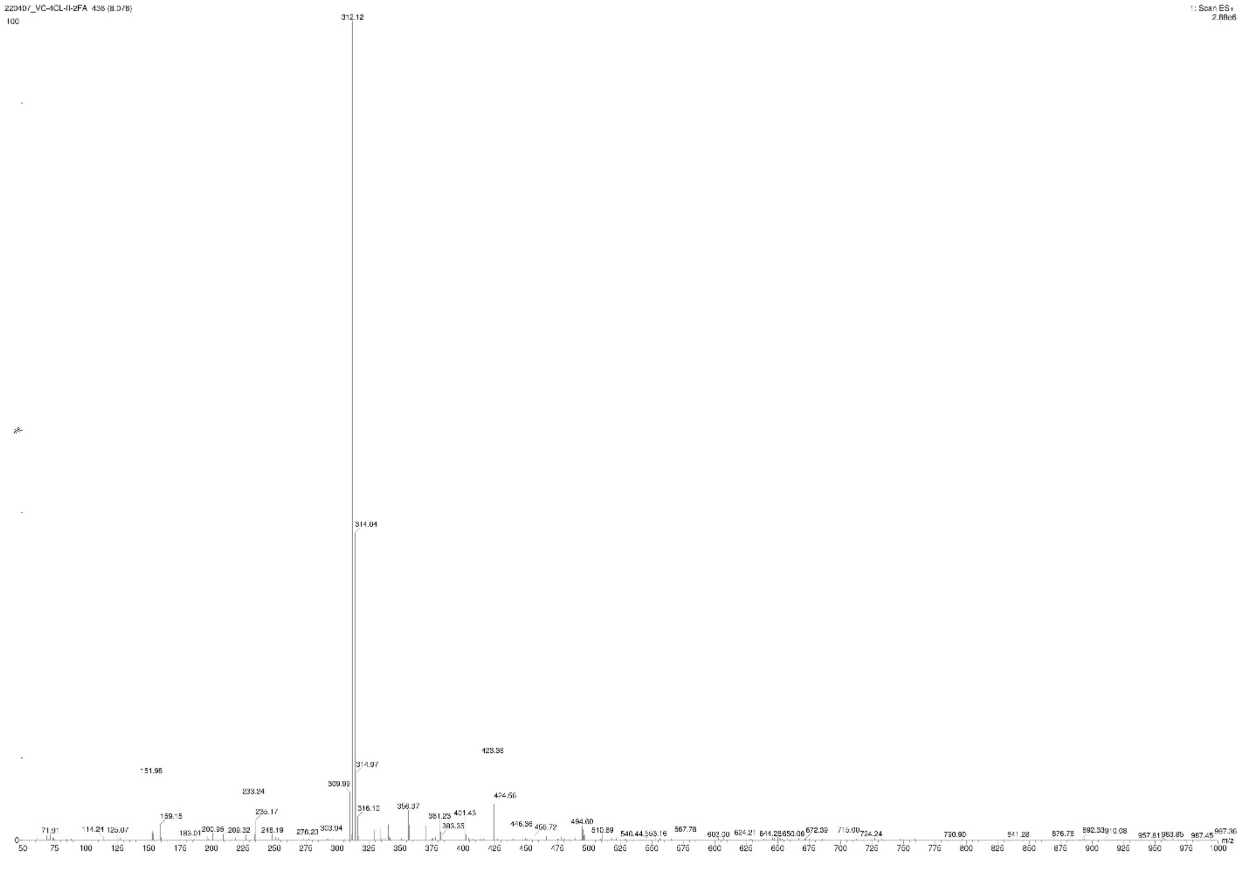

^{1}H-NMR (500 MHz, $CDCl_3$)

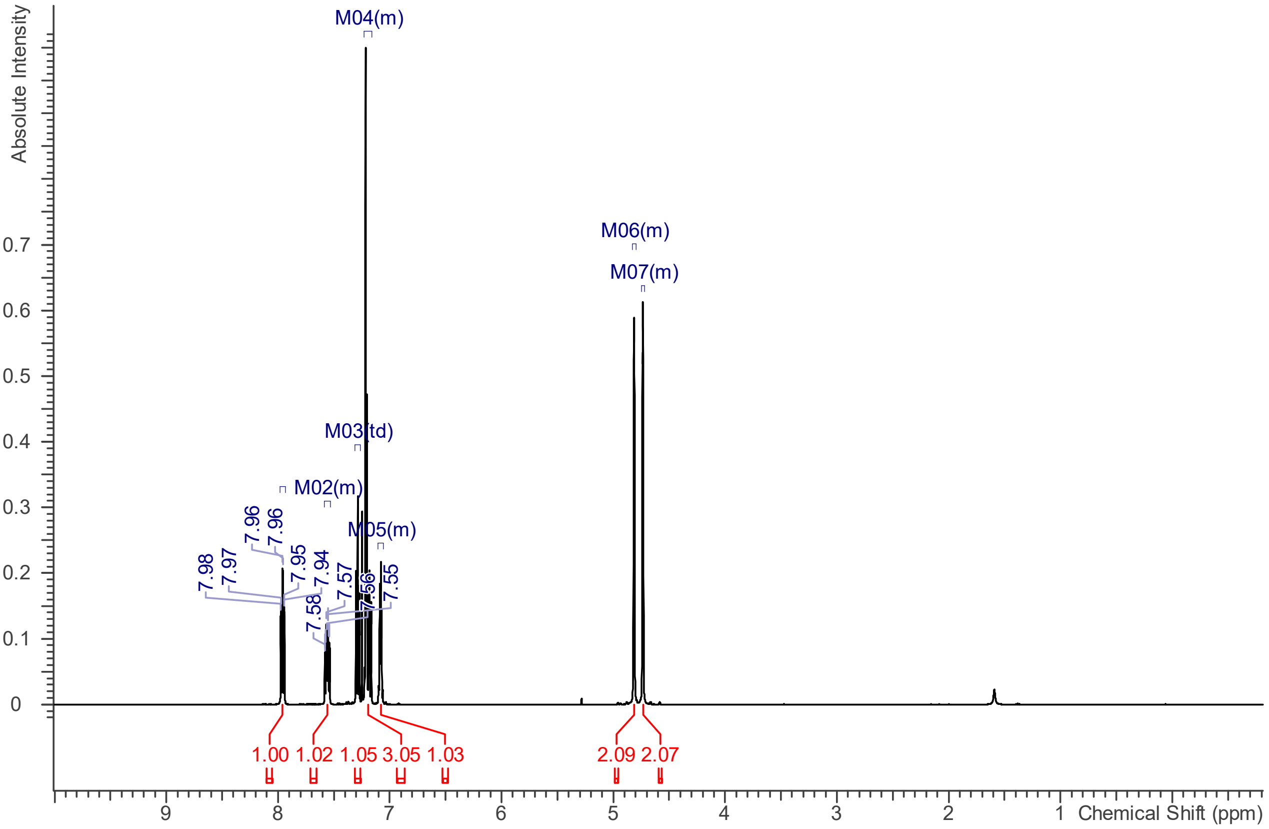

^{13}C-NMR (126 MHz, $CDCl_3$)

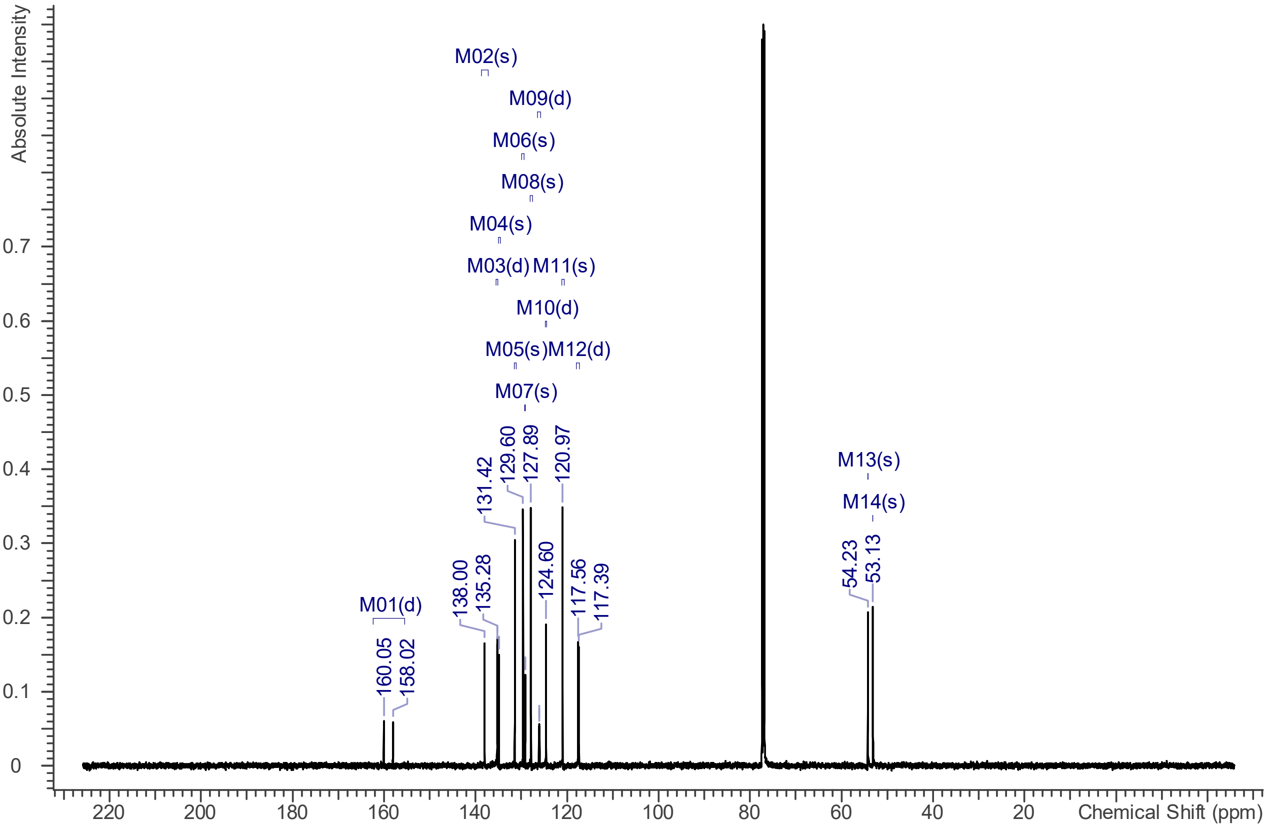

2.11. *4-Bromo-2-[(2-fluorophenyl)sulfonyl]isoindoline* **2k**

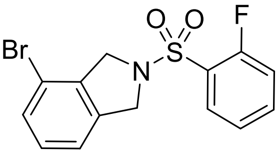

UPLC/MS

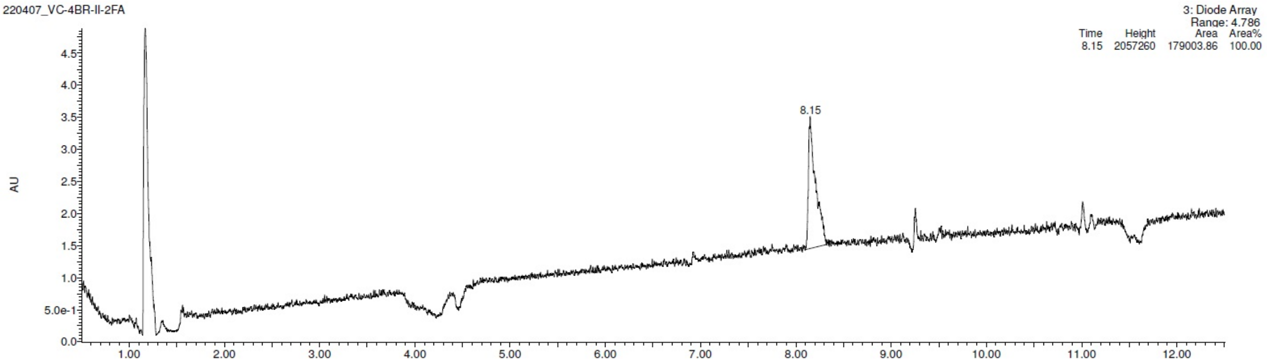

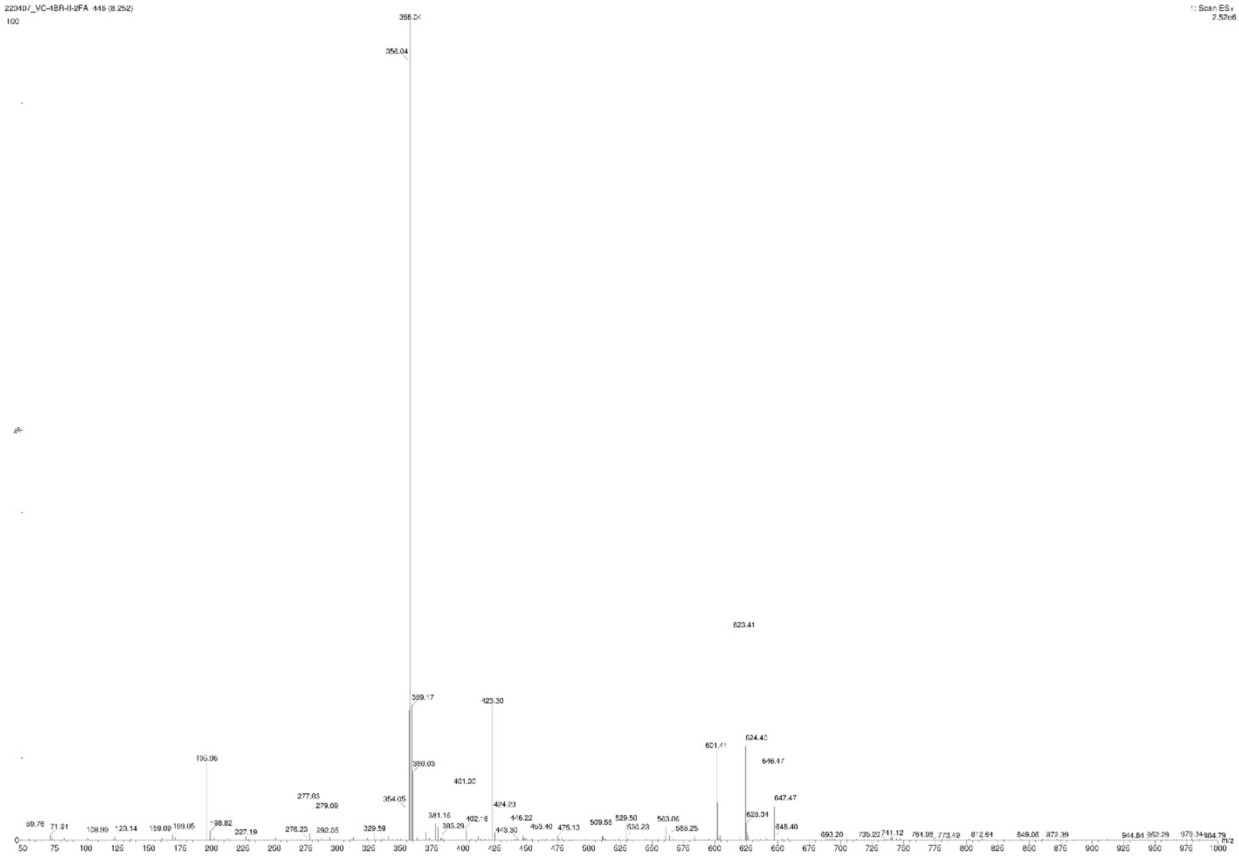

^{1}H-NMR (500 MHz, CDCl$_3$)

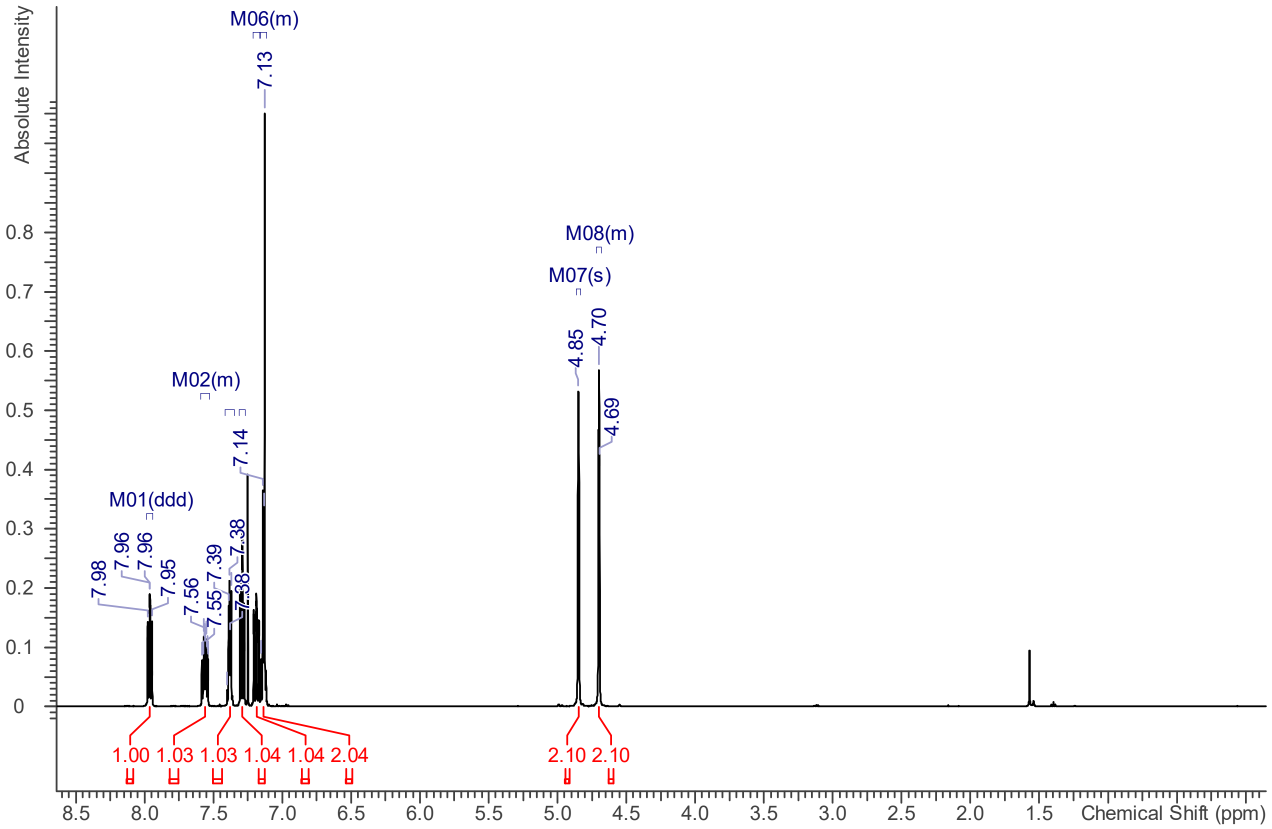

^{13}C-NMR (126 MHz, CDCl$_3$)

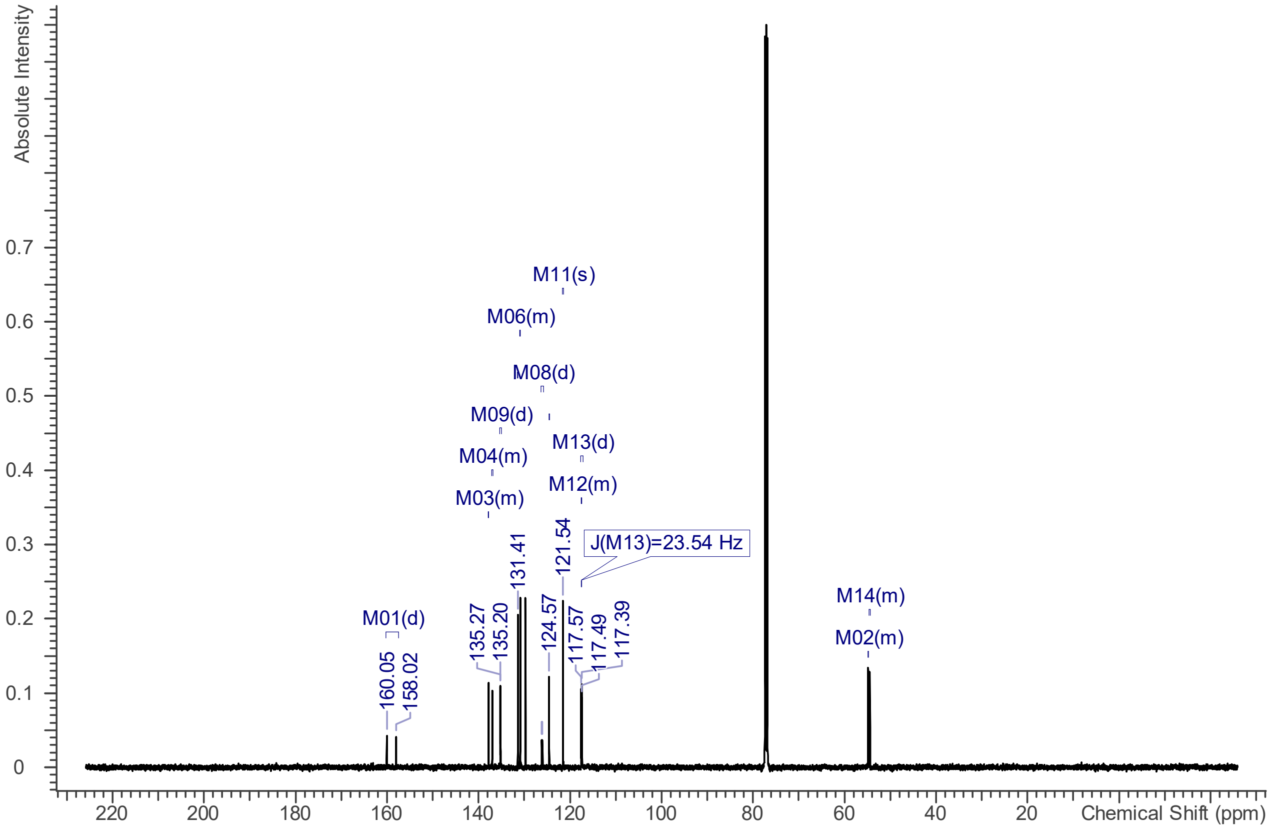

2.12. 5-Bromo-2-[(2-fluorophenyl)sulfonyl]isoindoline **2l**

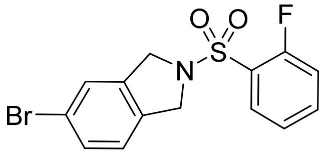

UPLC/MS

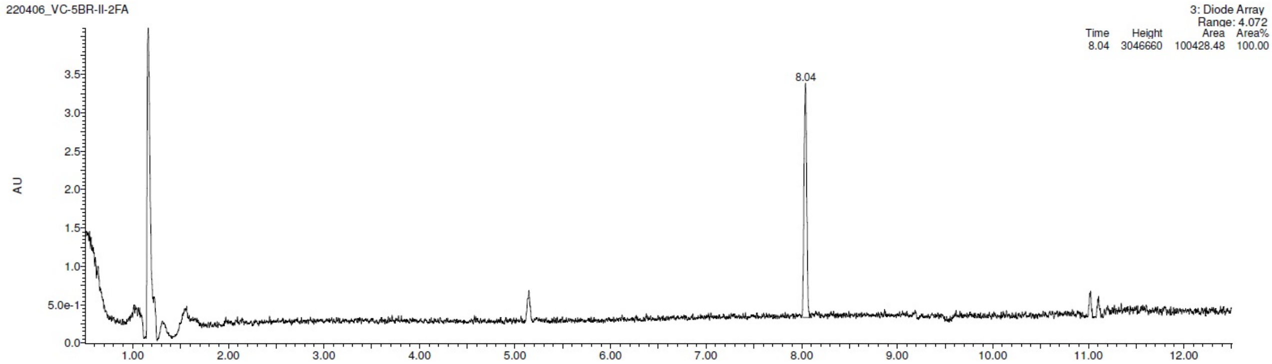

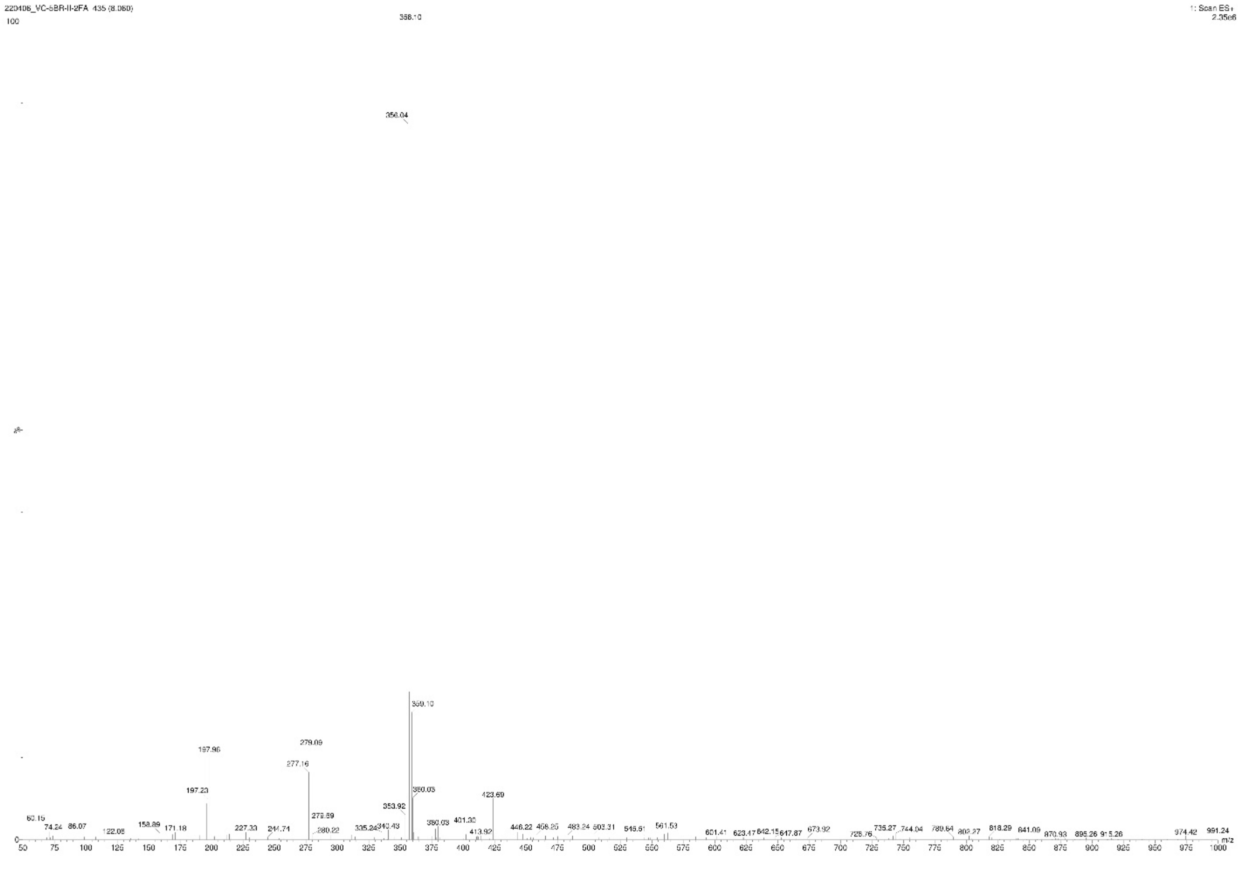

^{1}H-NMR (500 MHz, $CDCl_3$)

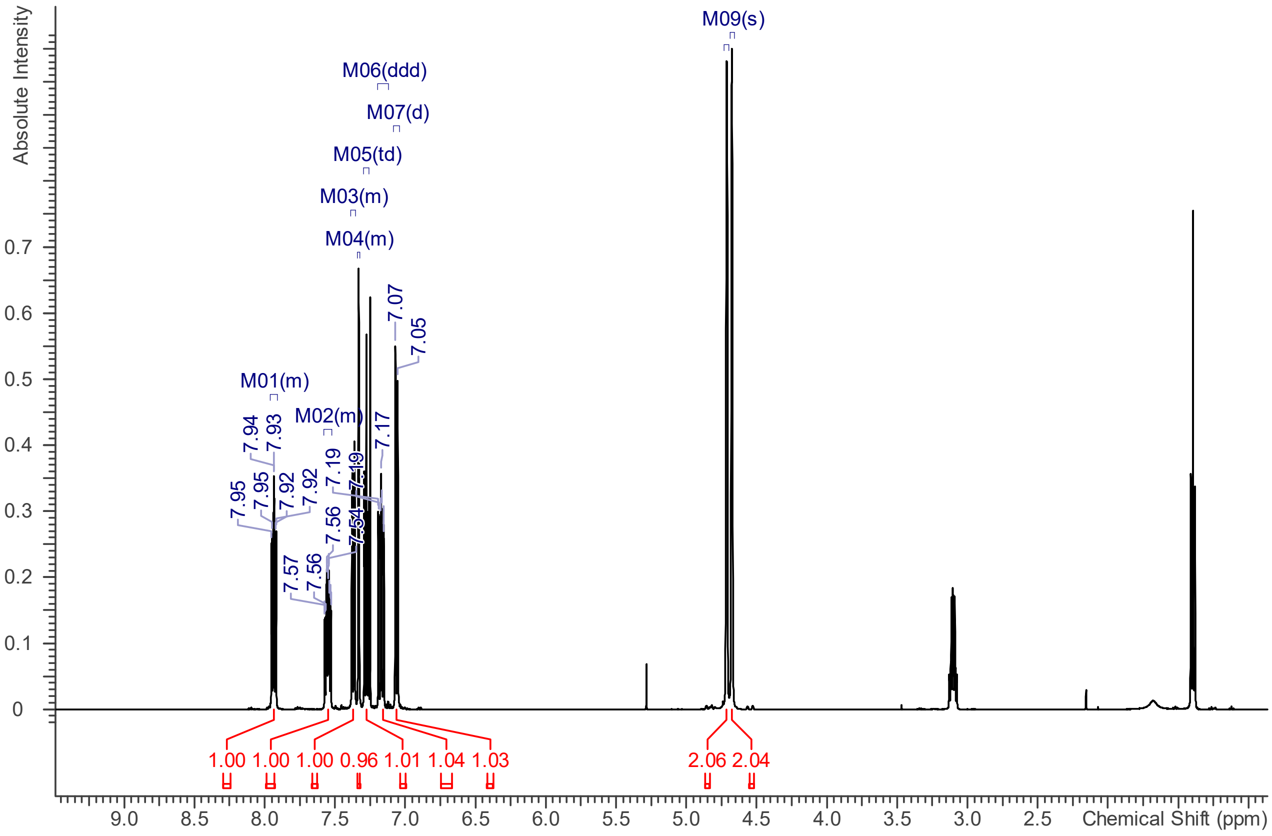

^{13}C-NMR (126 MHz, $CDCl_3$)

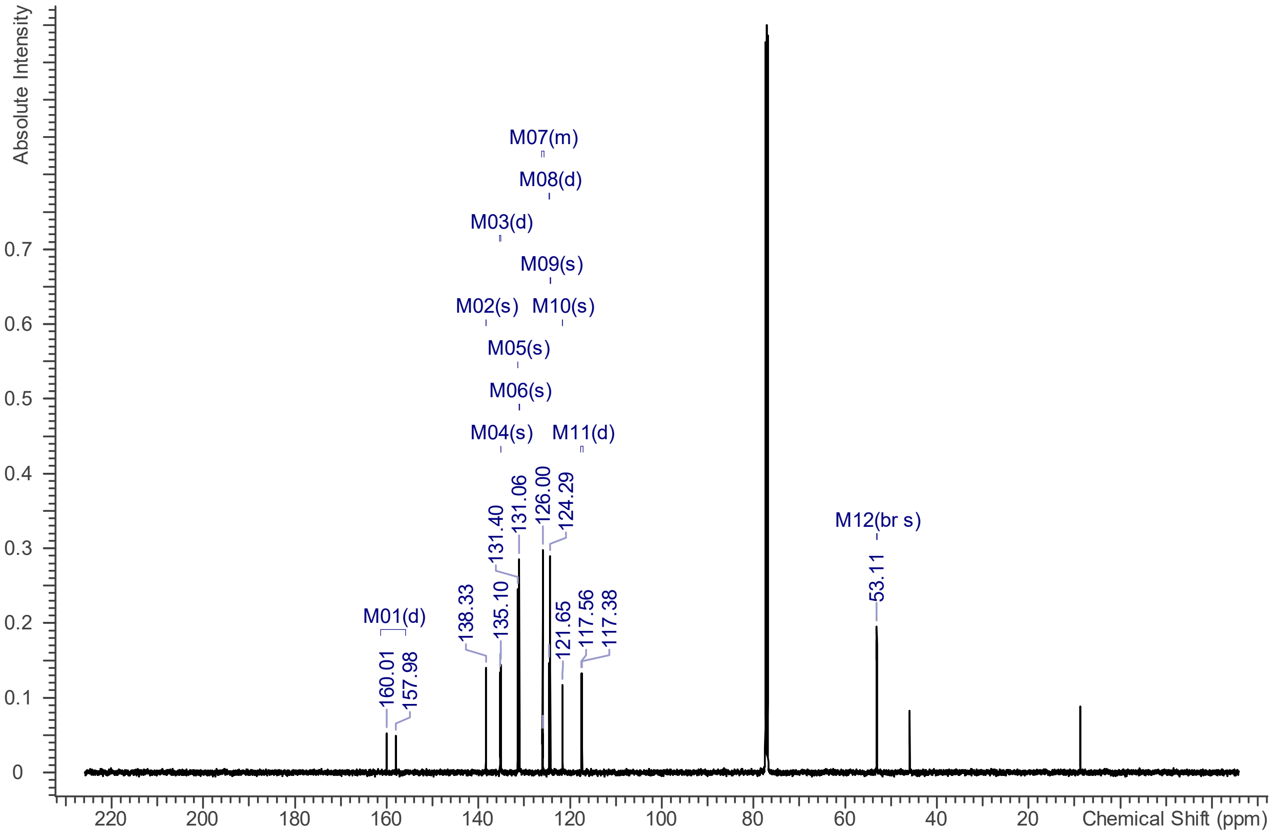

3. UPLC/MS, ^{1}H NMR and ^{13}C NMR spectra of final compounds 3a–l

3.1. 2-{[4-(Piperazin-1-yl)phenyl]sulfonyl}isoindoline **3a**

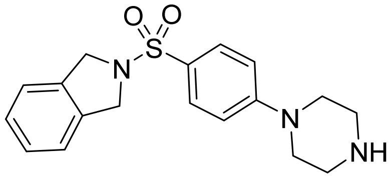

UPLC/MS

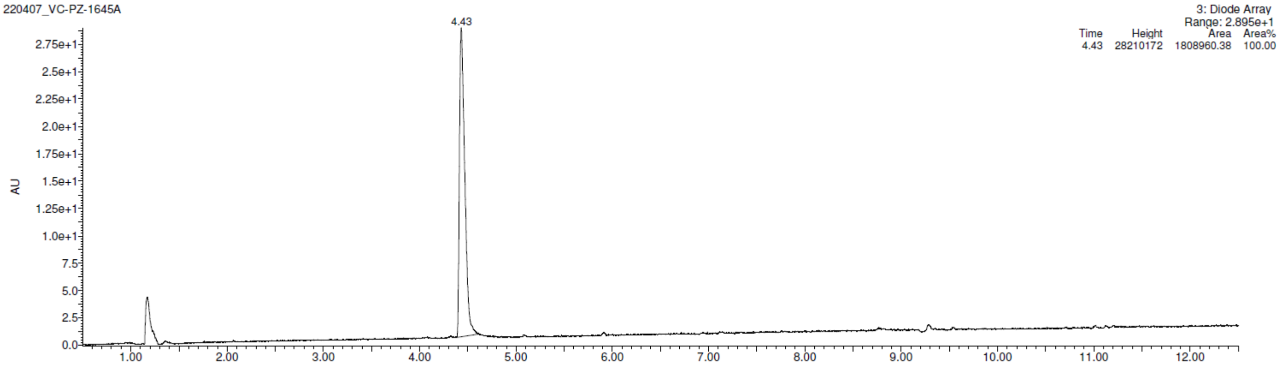

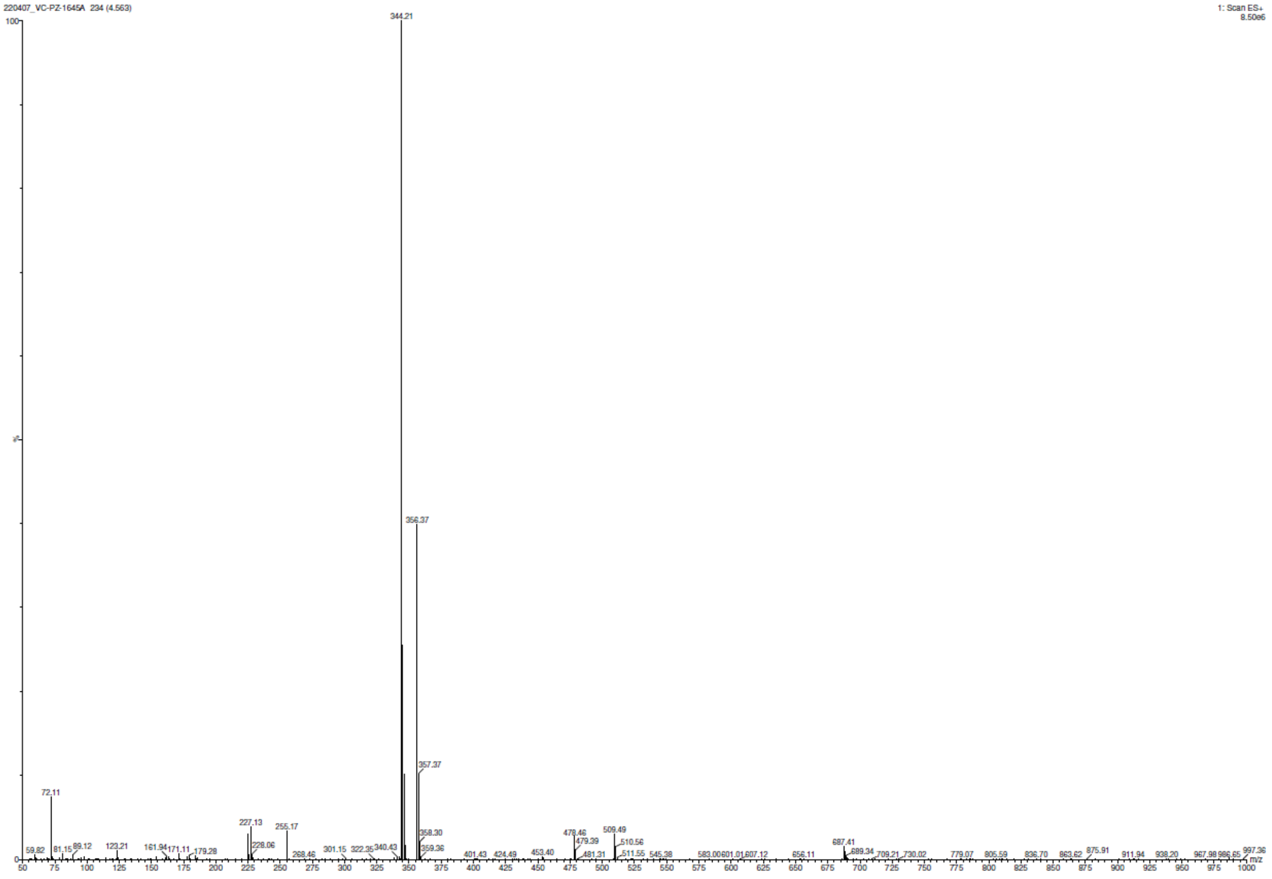

^{1}H-NMR (500 MHz, $CDCl_3$)

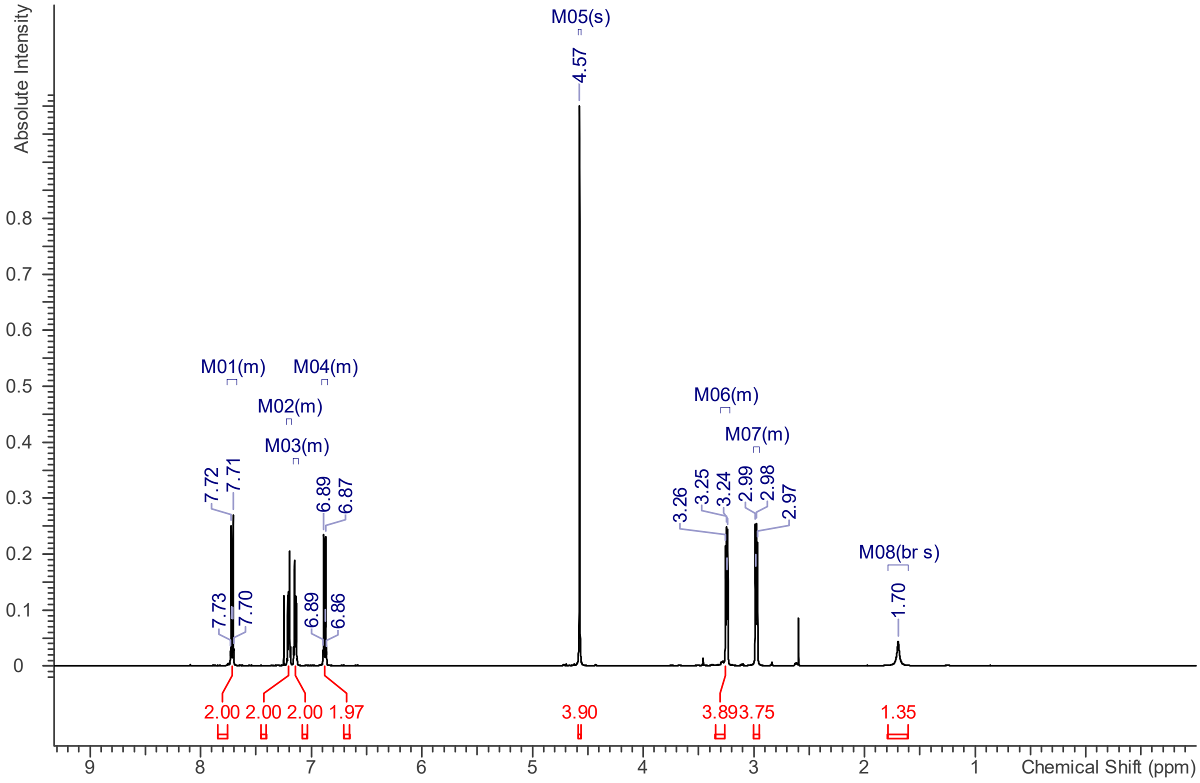

^{13}C-NMR (126 MHz, DMSO-d_6)

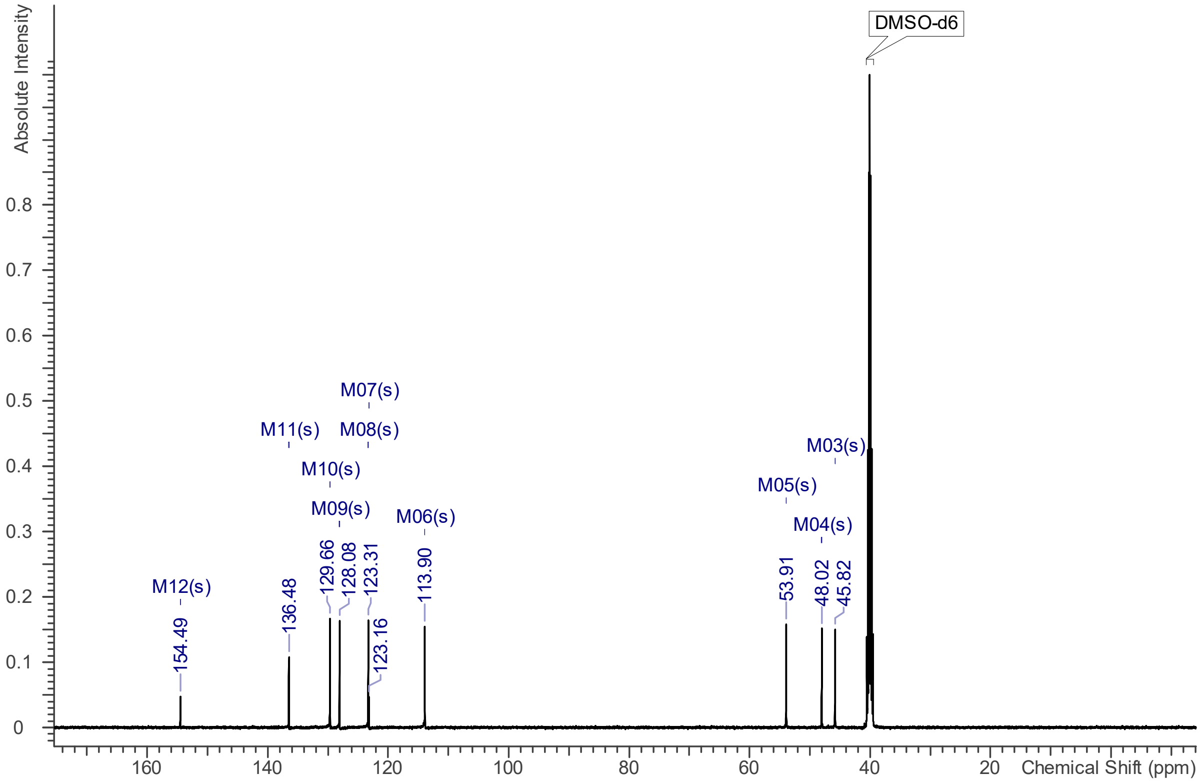

3.2. 4-Chloro-2-{[4-(piperazin-1-yl)phenyl]sulfonyl}isoindoline **3b**

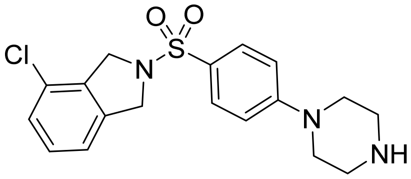

UPLC/MS

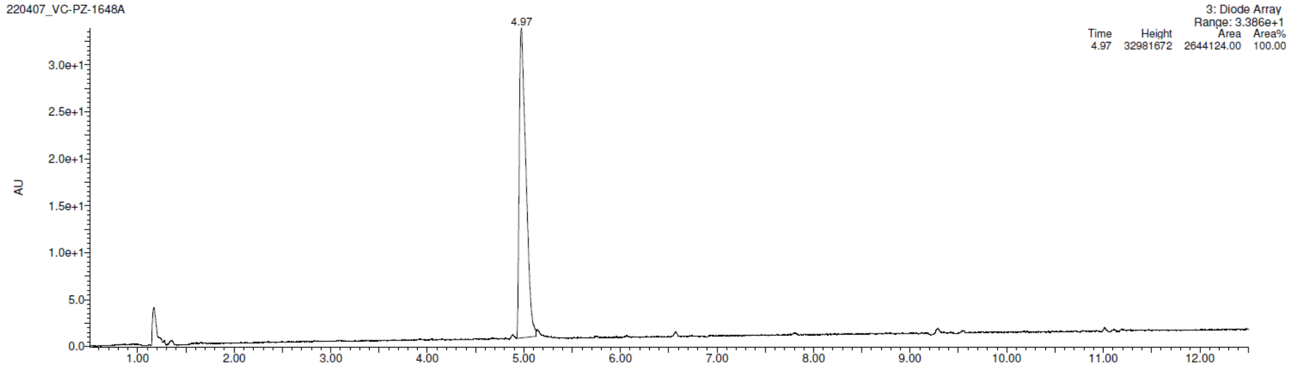

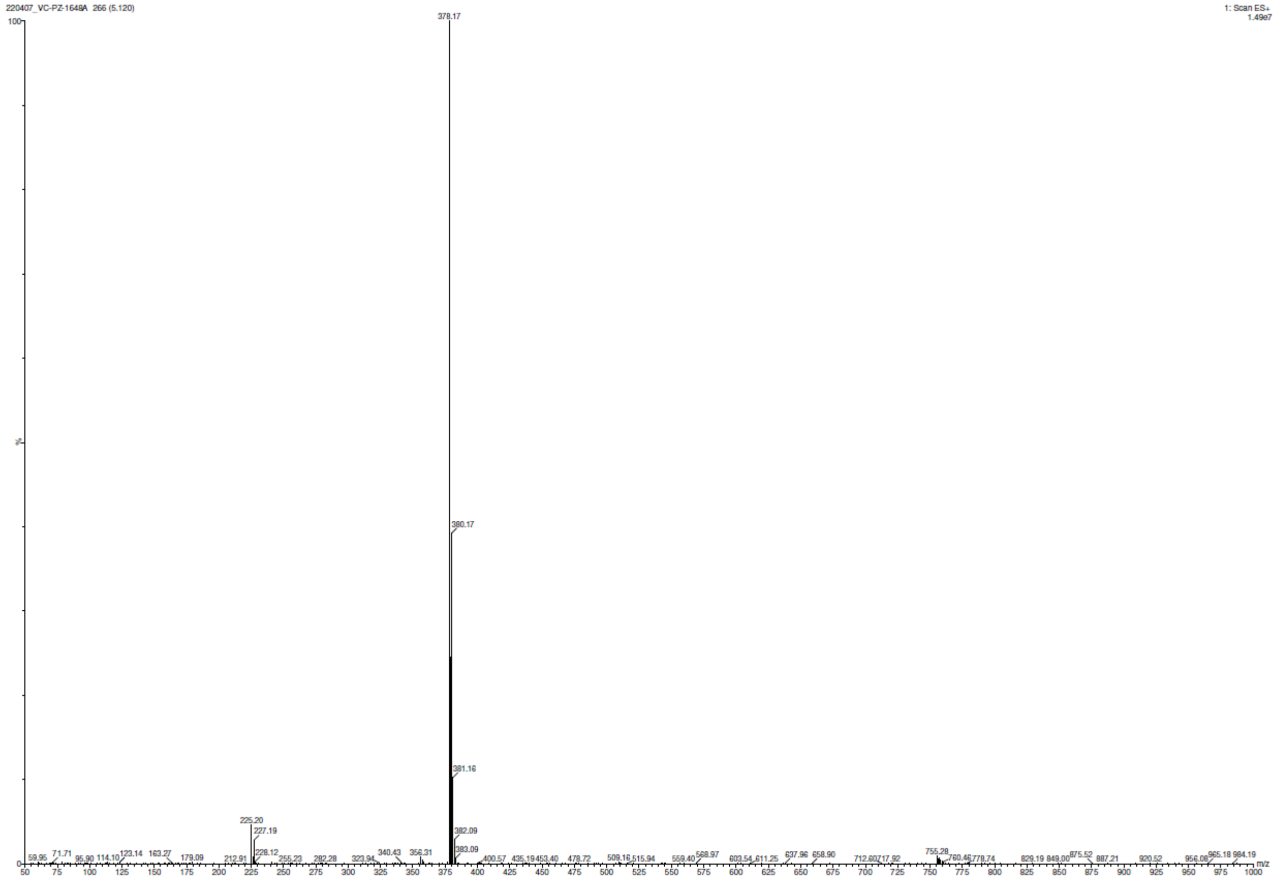

^{1}H-NMR (500 MHz, DMSO-d_6)

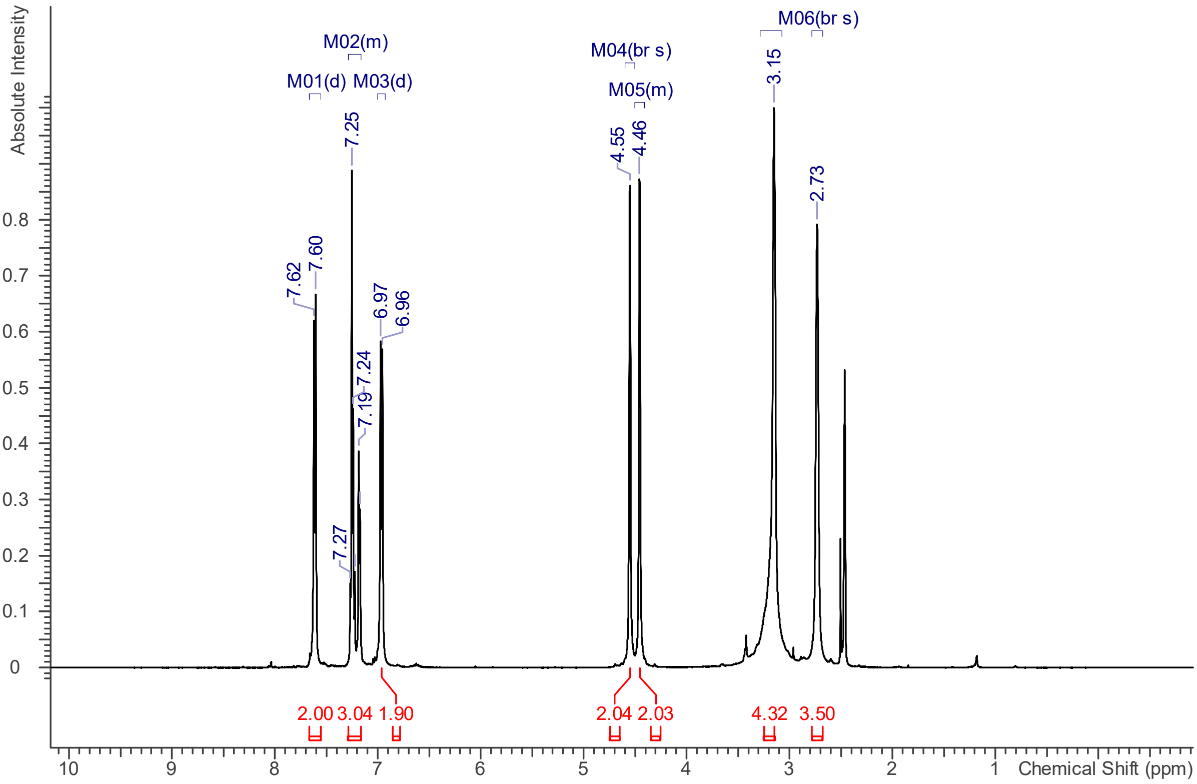

^{13}C-NMR (126 MHz, DMSO-d_6)

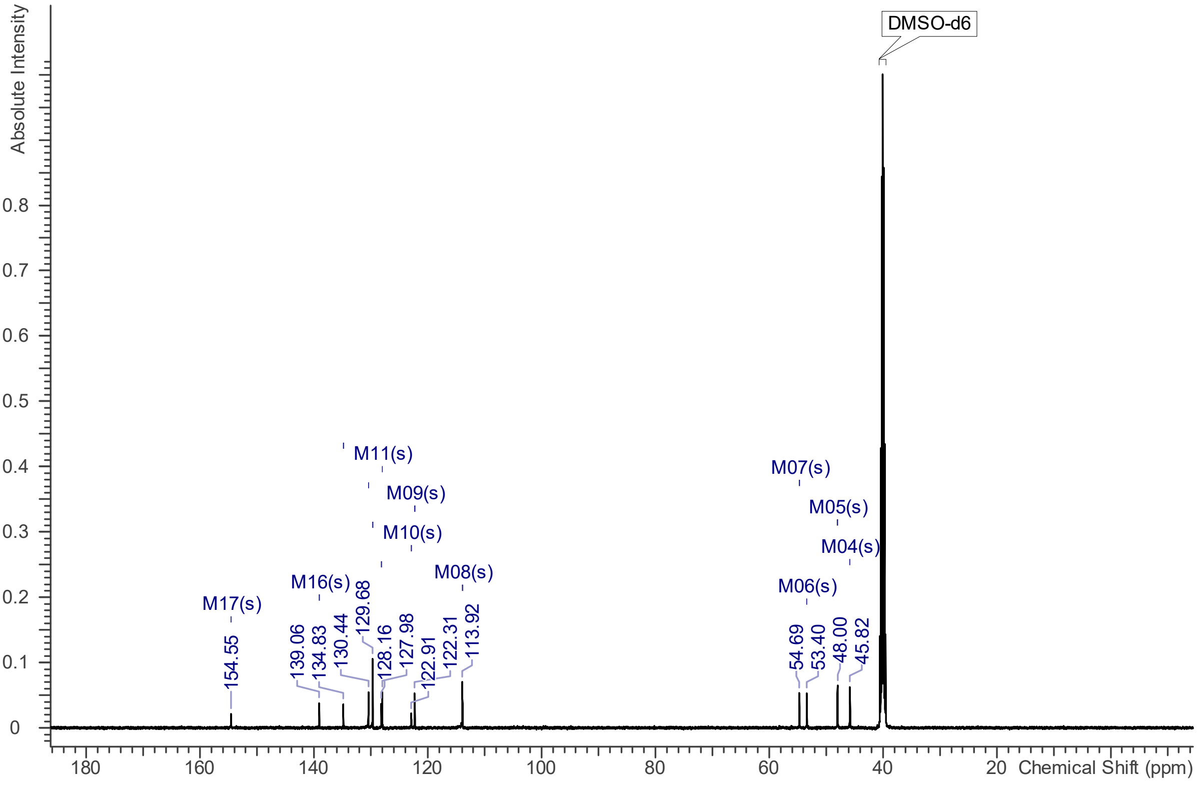

3.3. 4-Bromo-2-{[4-(piperazin-1-yl)phenyl]sulfonyl}isoindoline **3c**

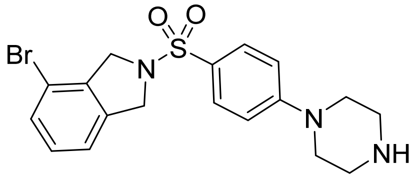

UPLC/MS

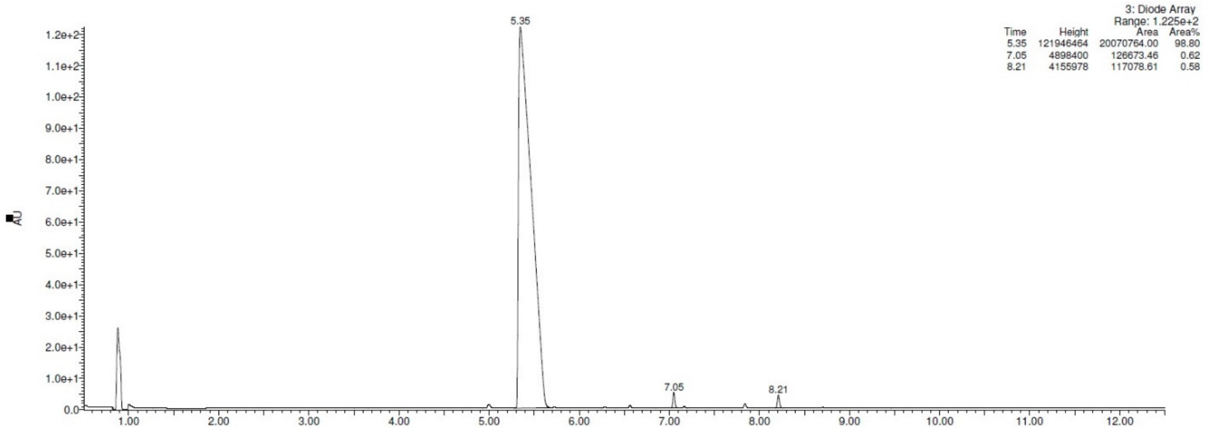

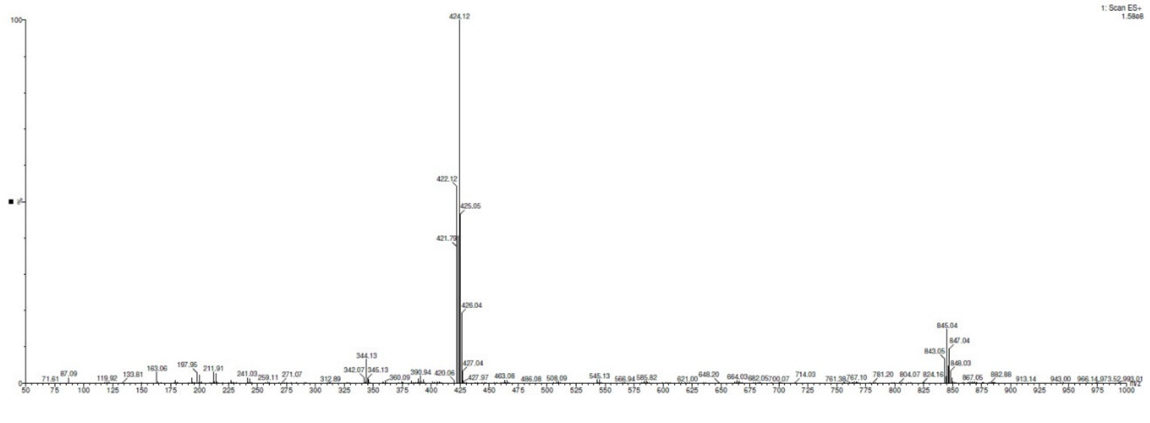

^{1}H-NMR (500 MHz, DMSO-d_6)

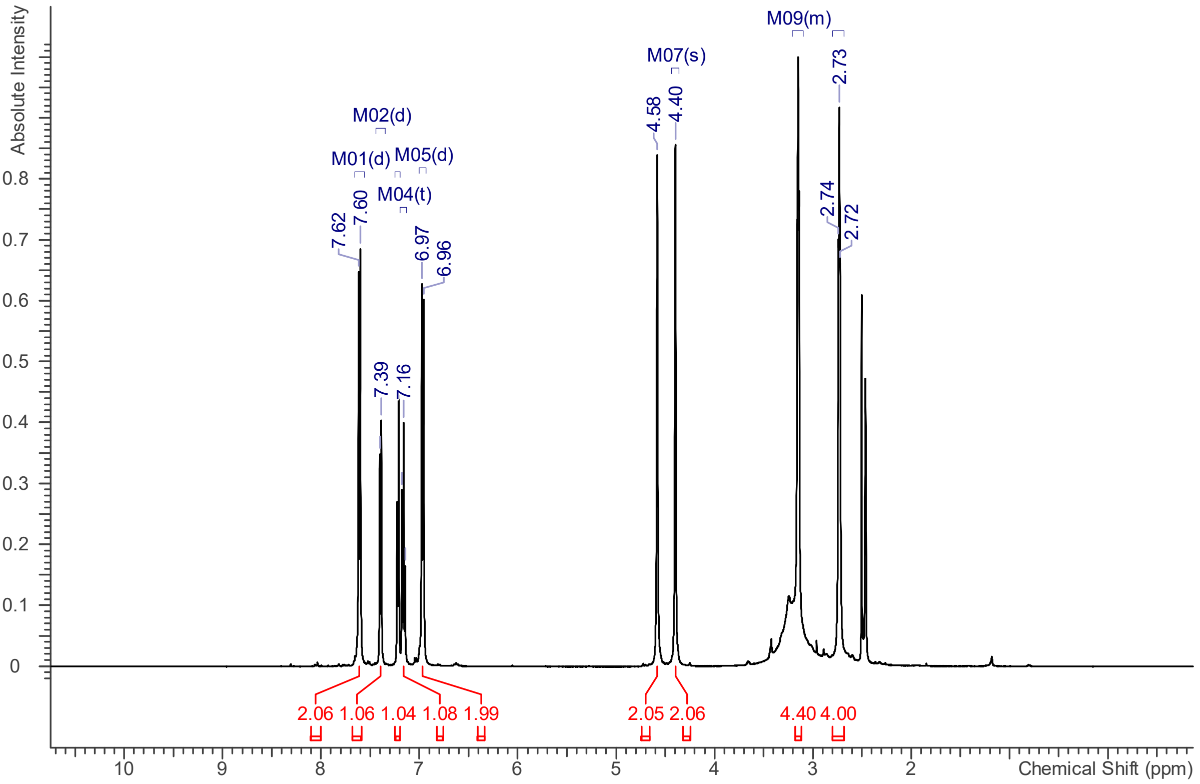

^{13}C-NMR (126 MHz, DMSO-d_6)

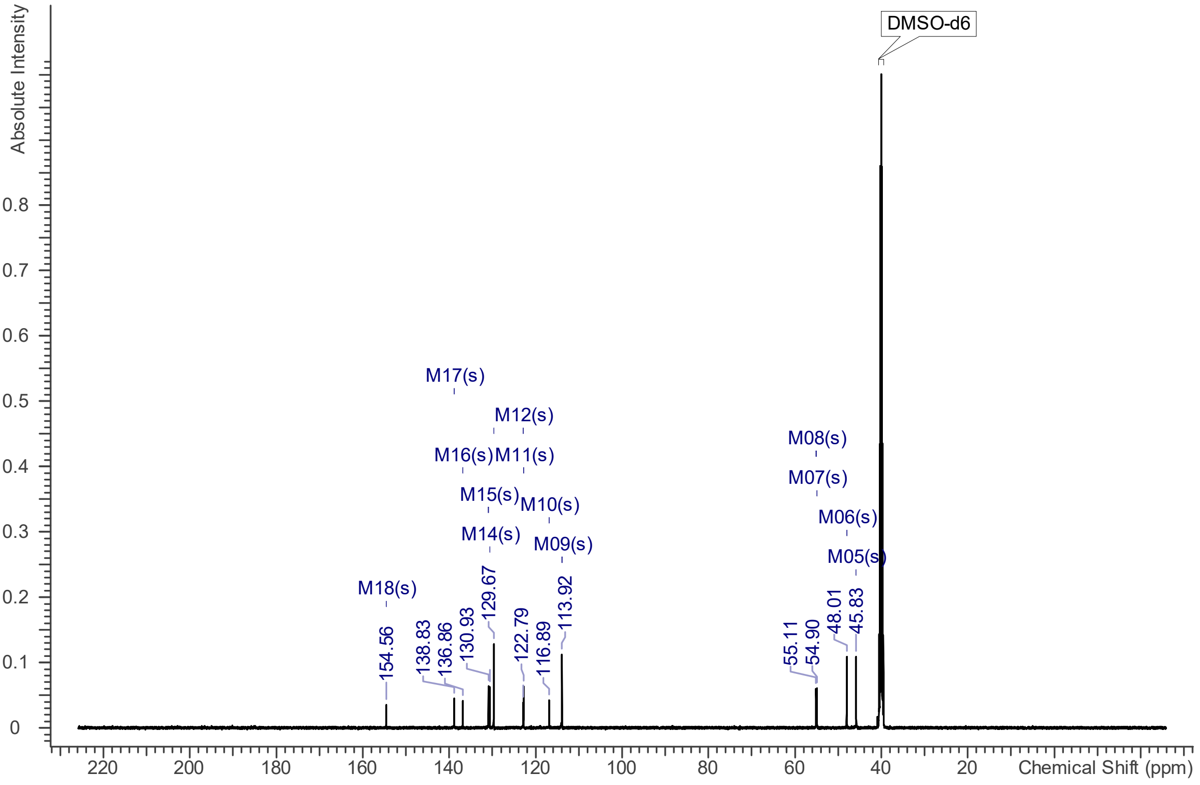

3.4. 5-Bromo-2-{[4-(piperazin-1-yl)phenyl]sulfonyl}isoindoline **3d**

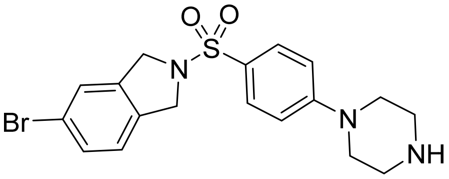

UPLC/MS

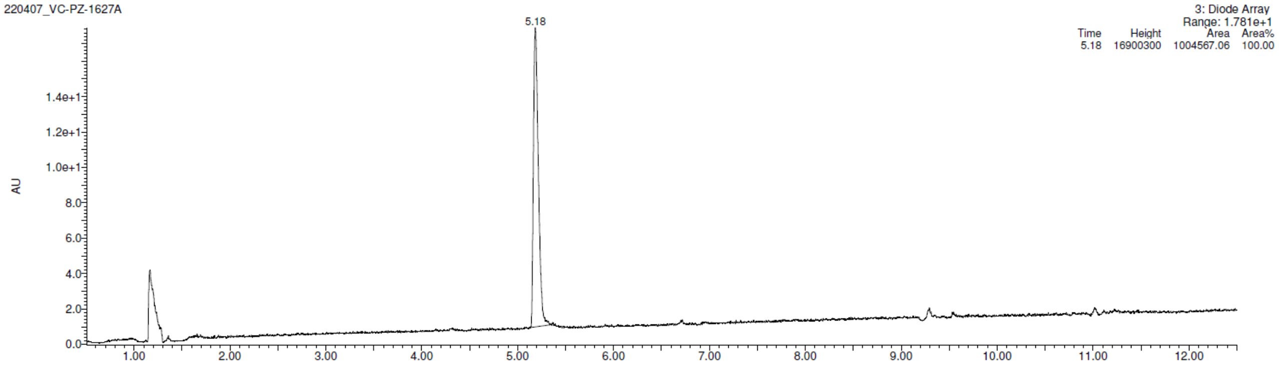

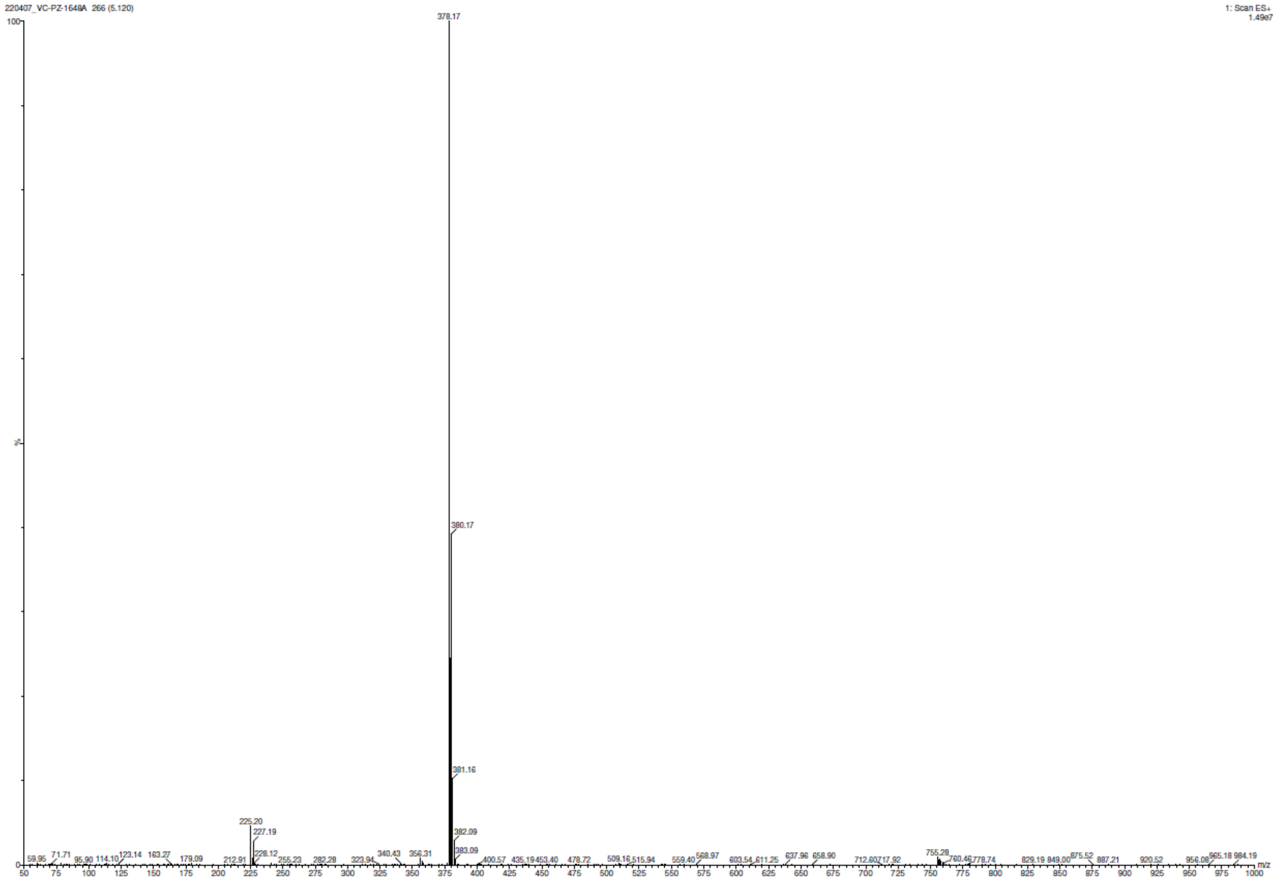

^{1}H-NMR (500 MHz, DMSO-d_6)

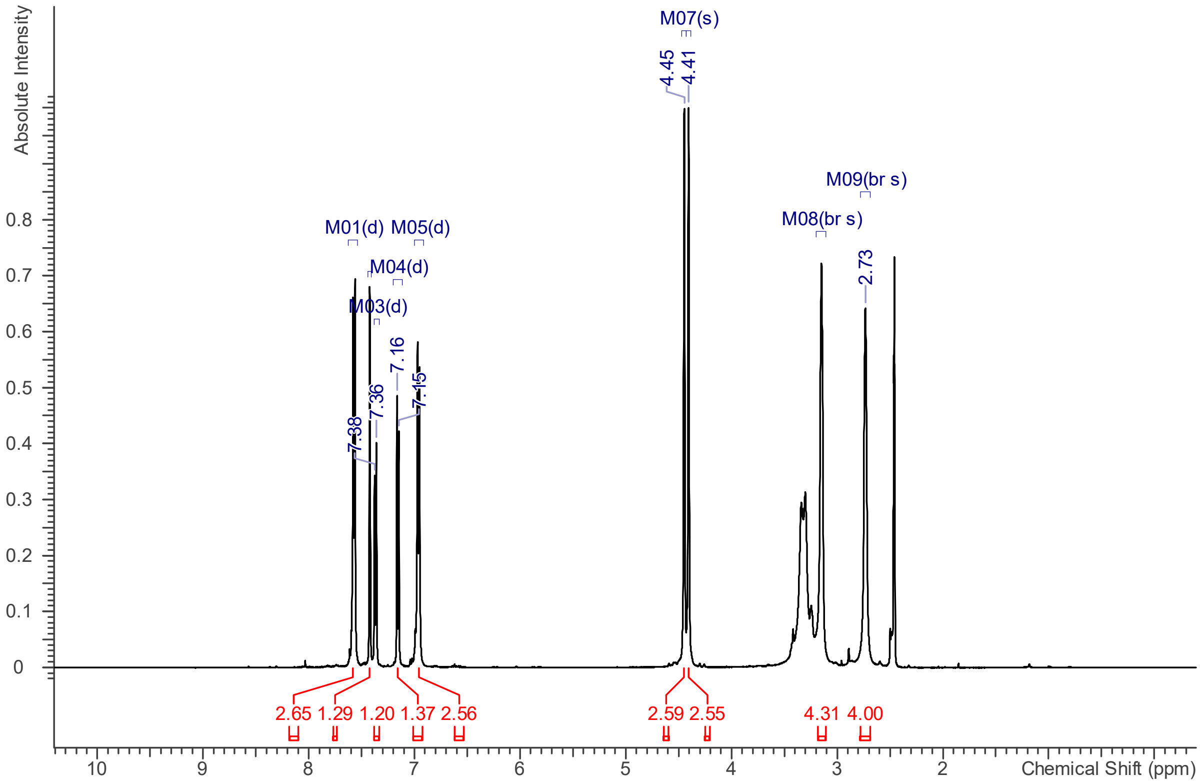

^{13}C-NMR (126 MHz, DMSO-d_6)

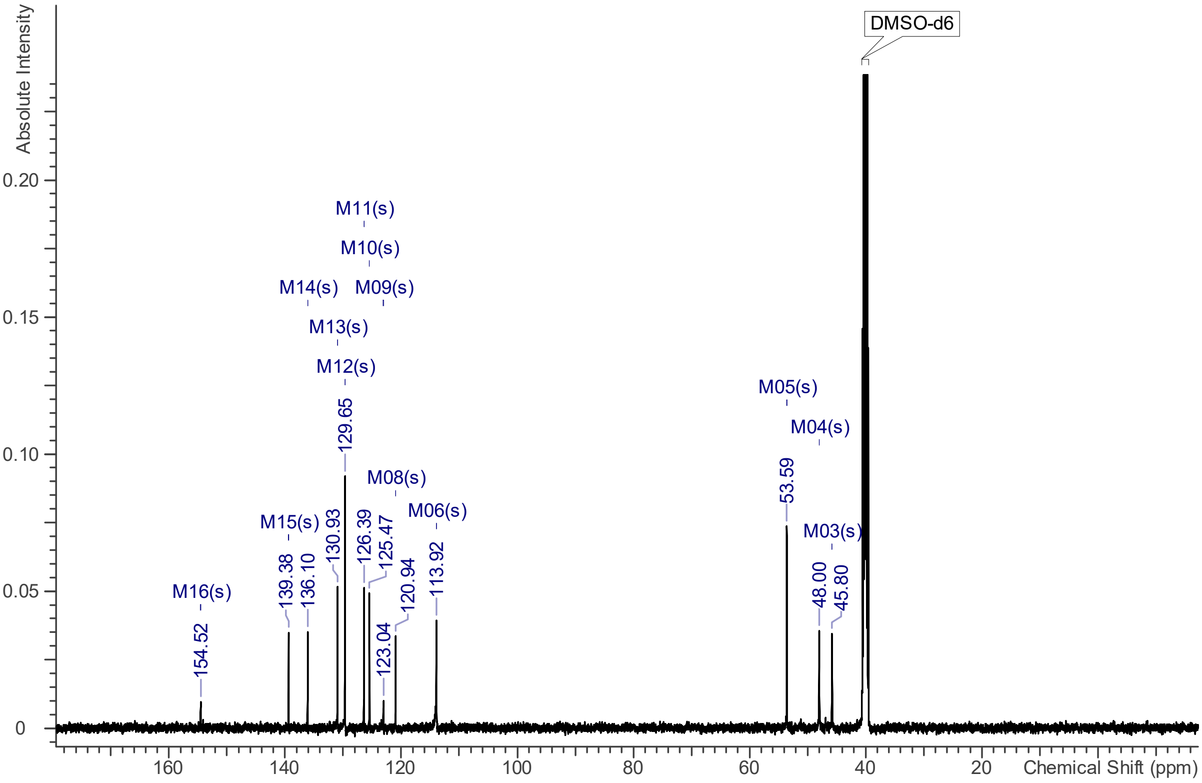

3.5. 2-{[3-(Piperazin-1-yl)phenyl]sulfonyl}isoindoline **3e**

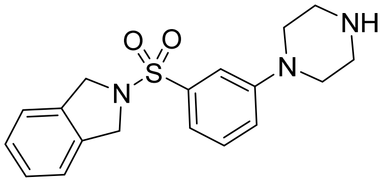

UPLC/MS

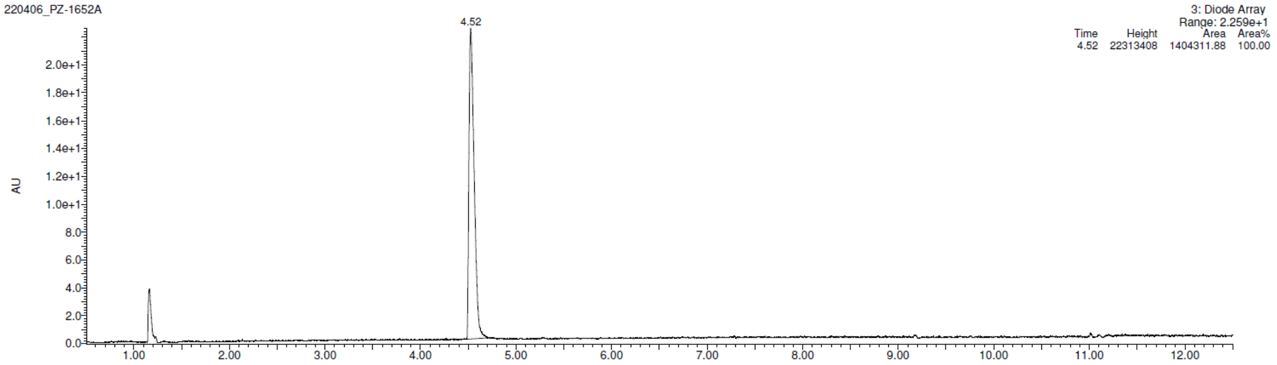

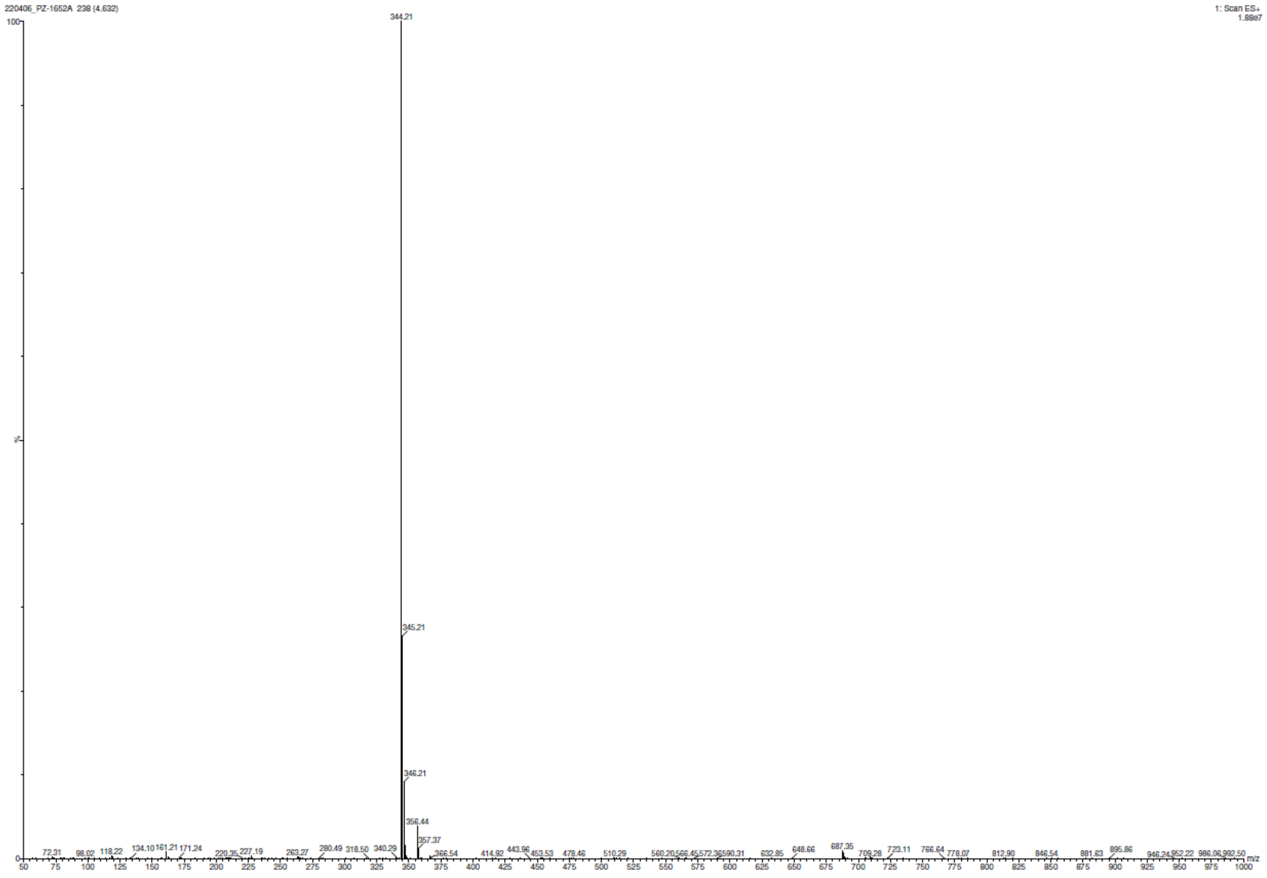

^{1}H-NMR (500 MHz, CDCl$_3$)

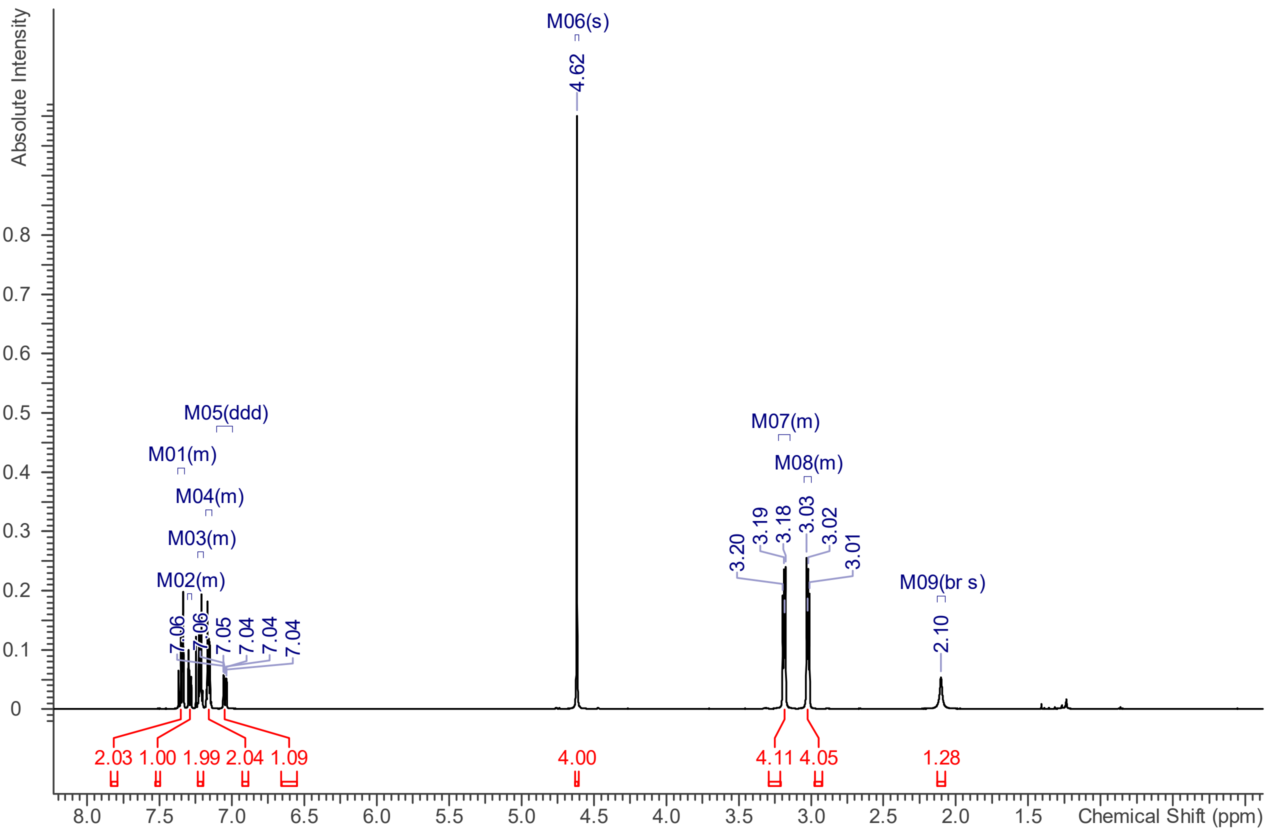

^{13}C-NMR (126 MHz, CDCl$_3$)

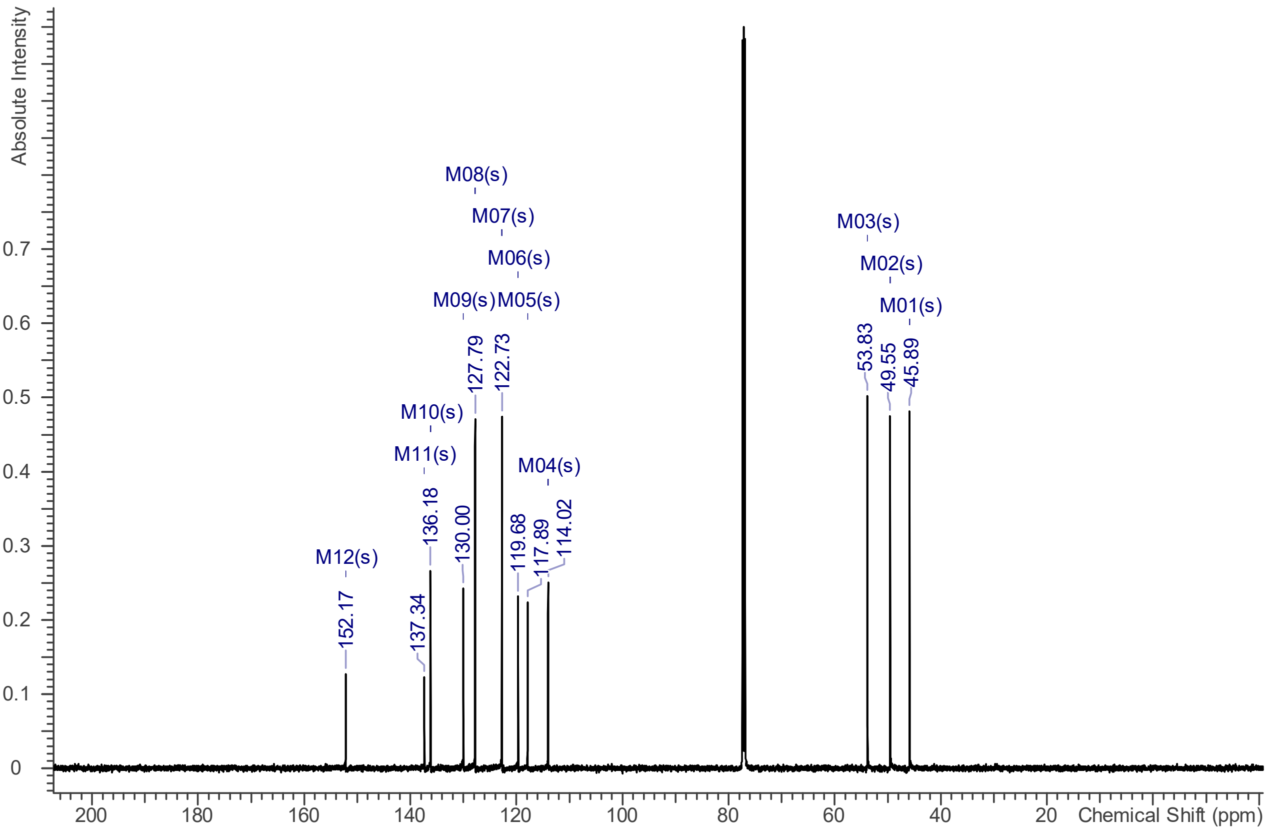

3.6. 4-Chloro-2-{[3-(piperazin-1-yl)phenyl]sulfonyl}isoindoline **3f**

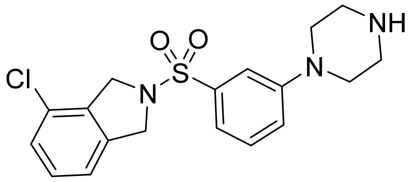

UPLC/MS

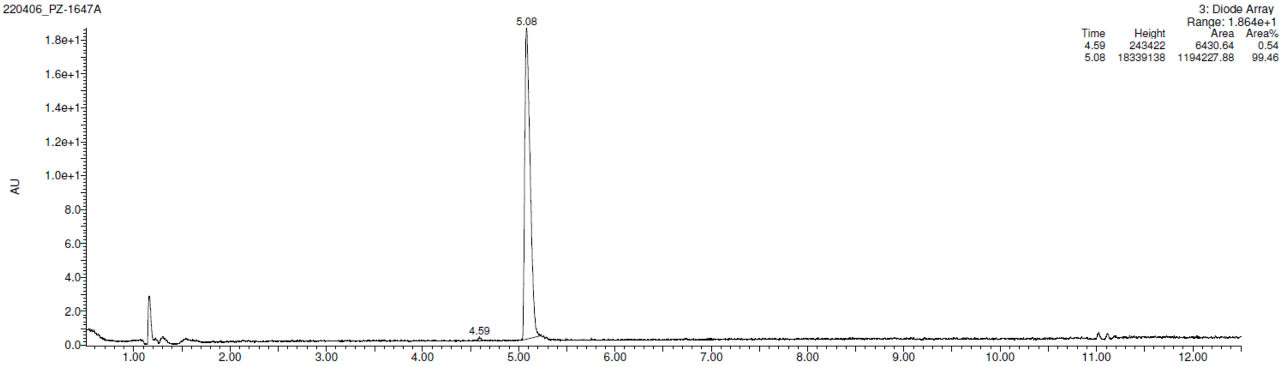

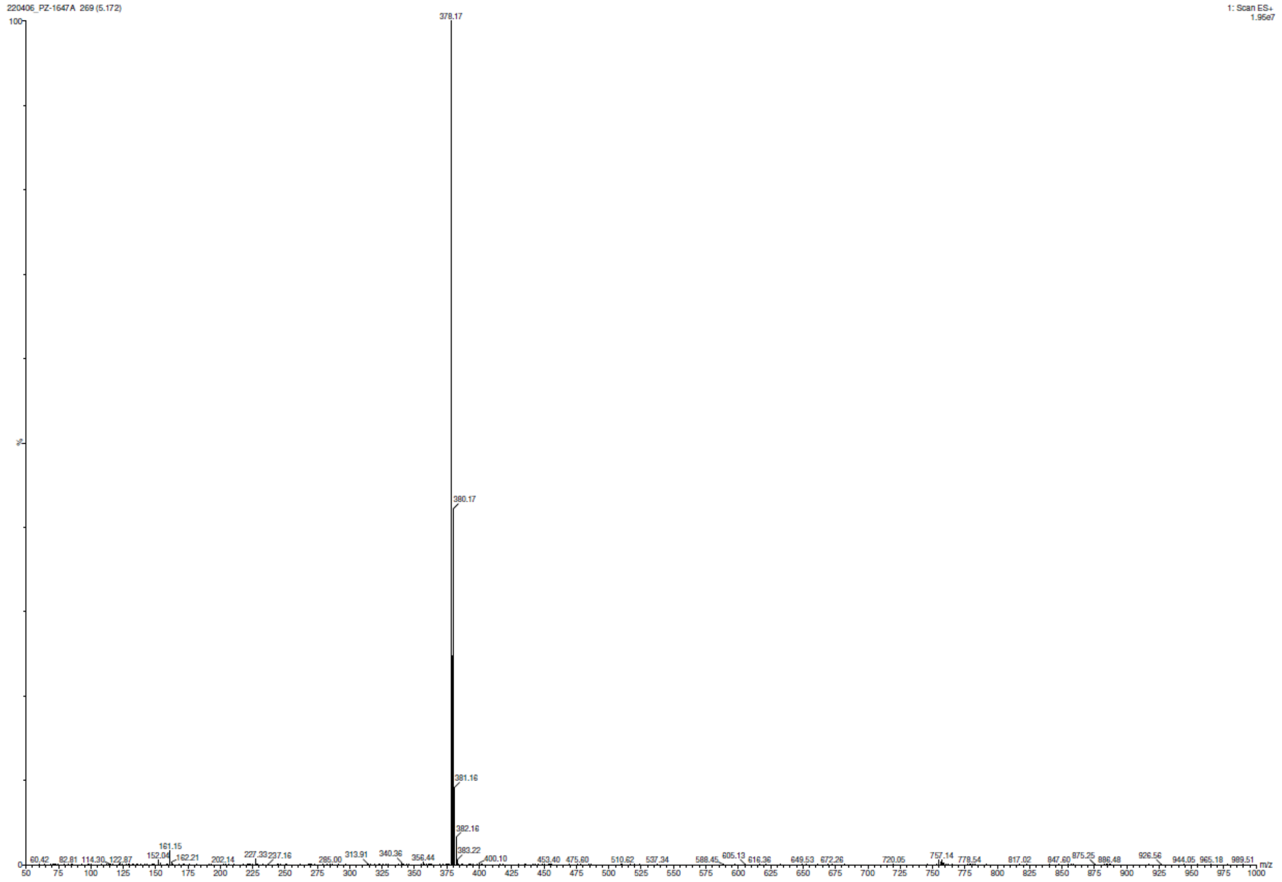

^{1}H-NMR (500 MHz, $CDCl_3$)

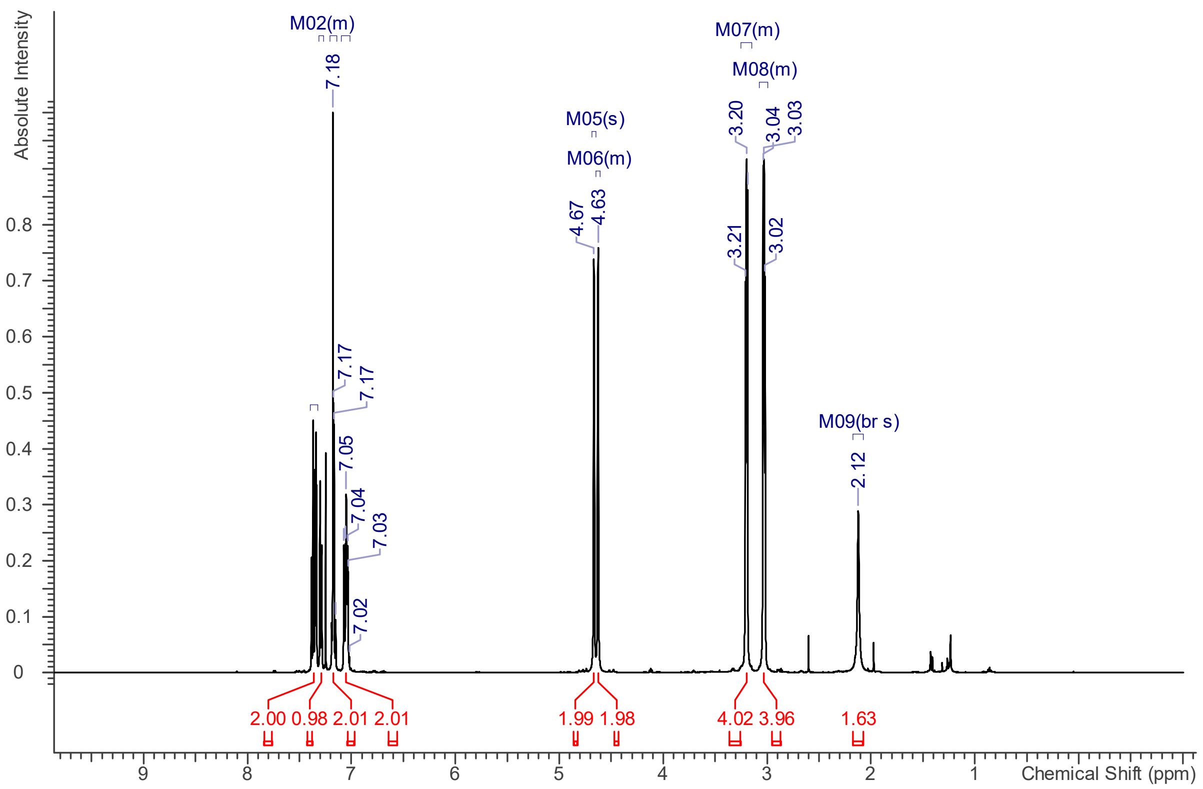

^{13}C-NMR (126 MHz, $CDCl_3$)

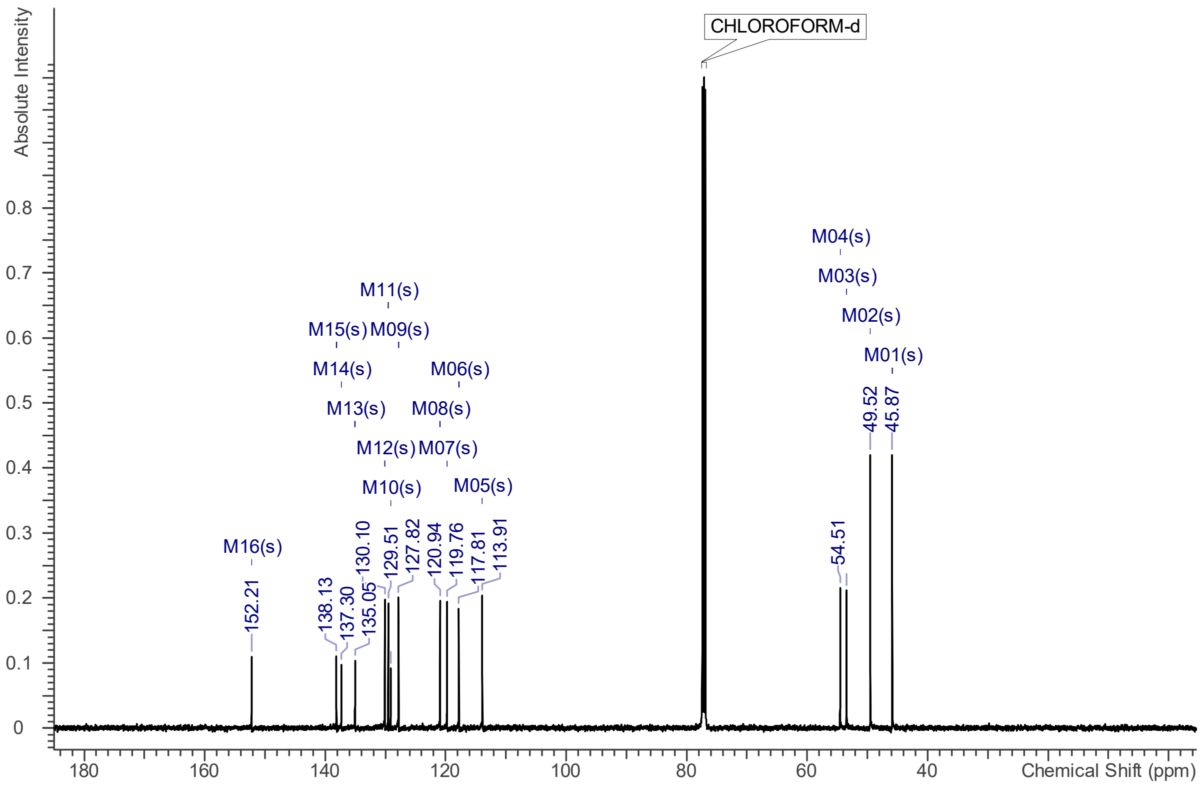

3.7. 4-Bromo-2-{[3-(piperazin-1-yl)phenyl]sulfonyl}isoindoline **3g**

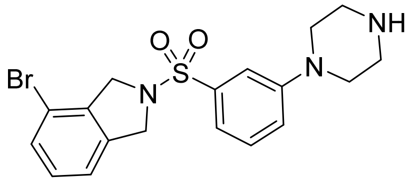

UPLC/MS

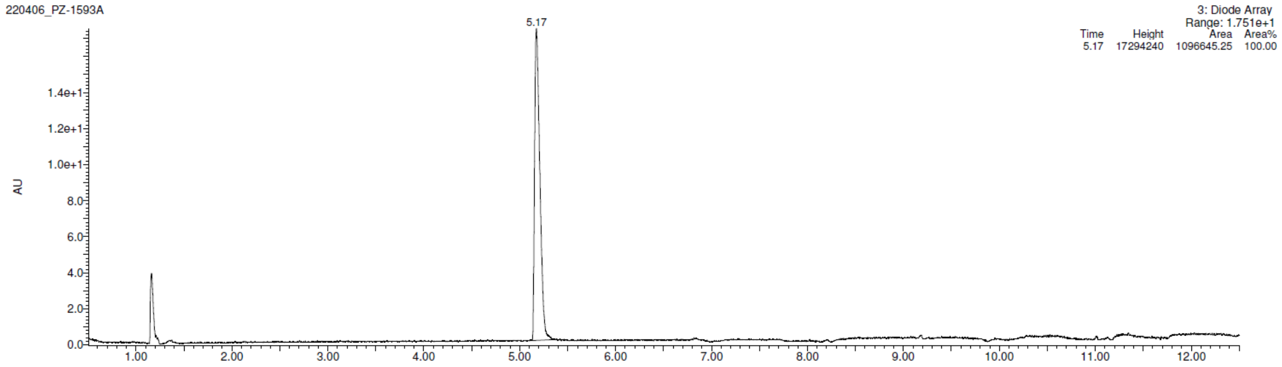

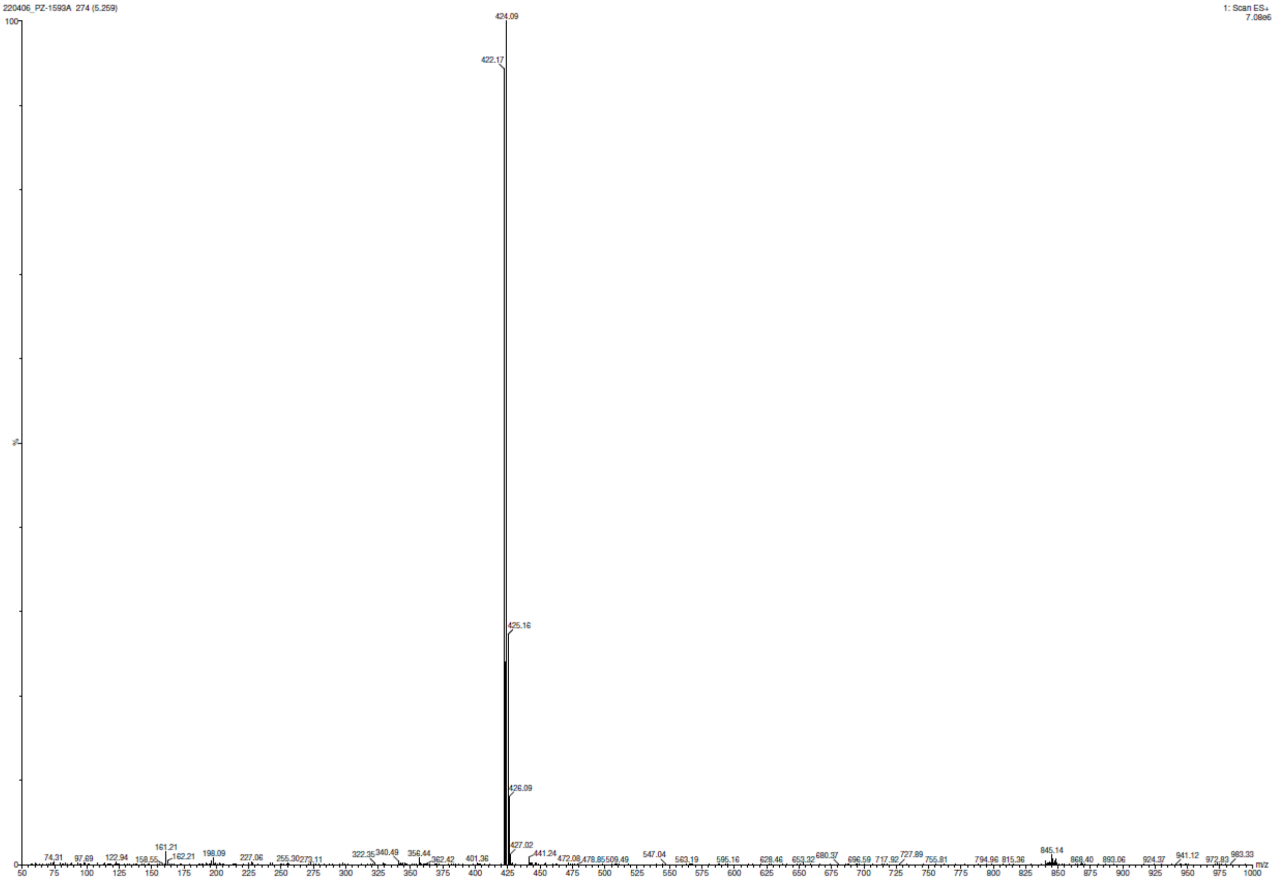

^{1}H-NMR (500 MHz, DMSO-d_6)

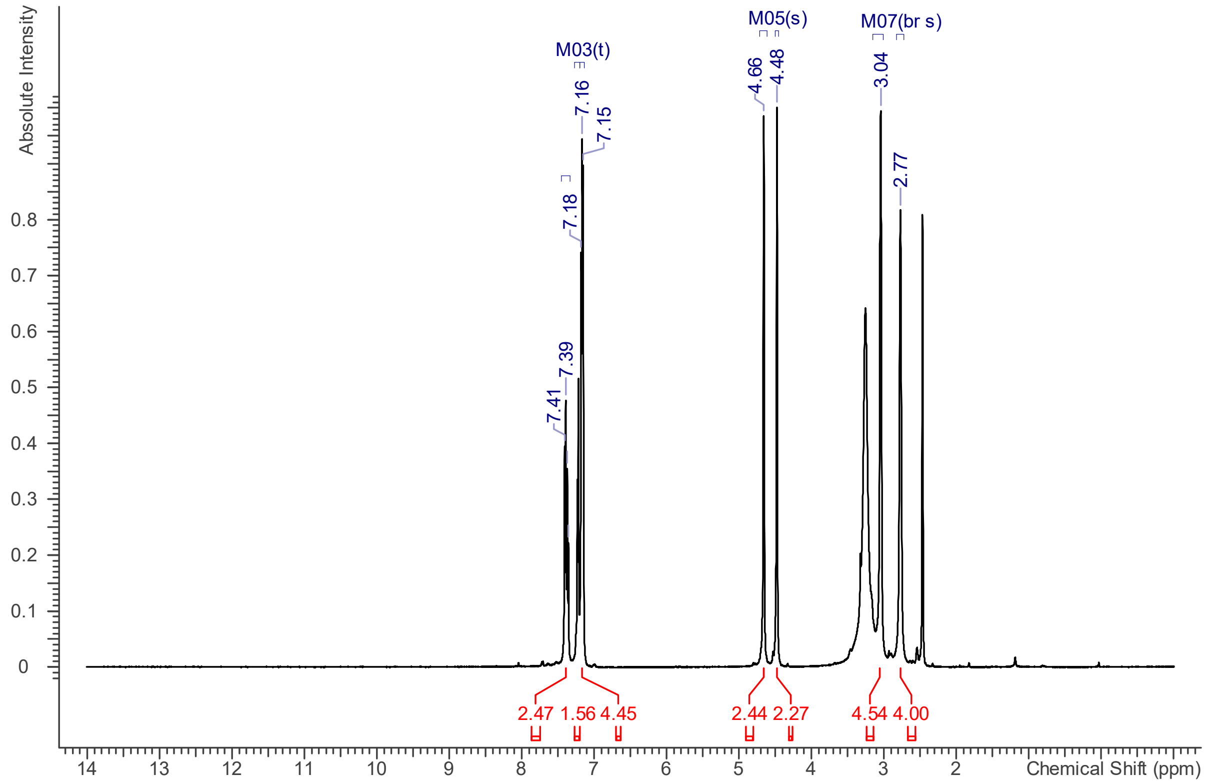

^{13}C-NMR (126 MHz, DMSO-d_6)

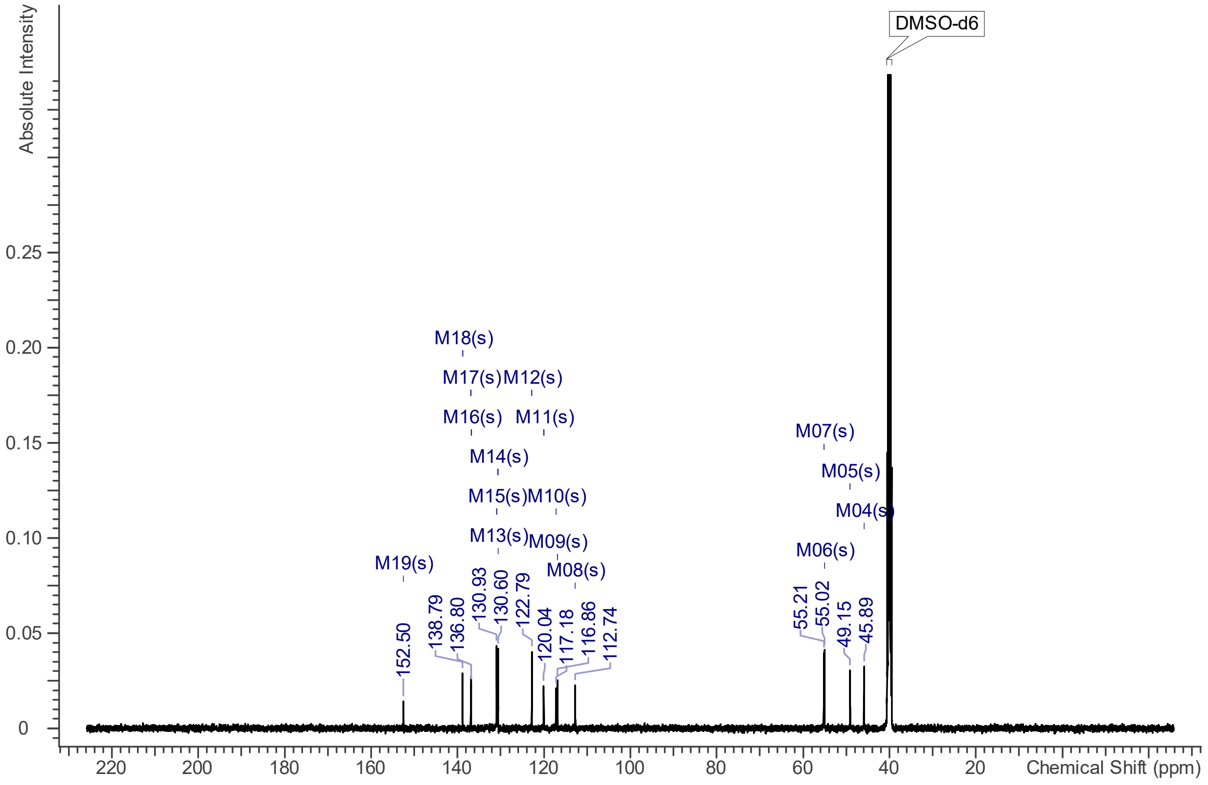

3.8. 5-Bromo-2-{[3-(piperazin-1-yl)phenyl]sulfonyl}isoindoline **3h**

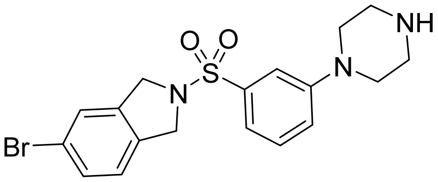

UPLC/MS

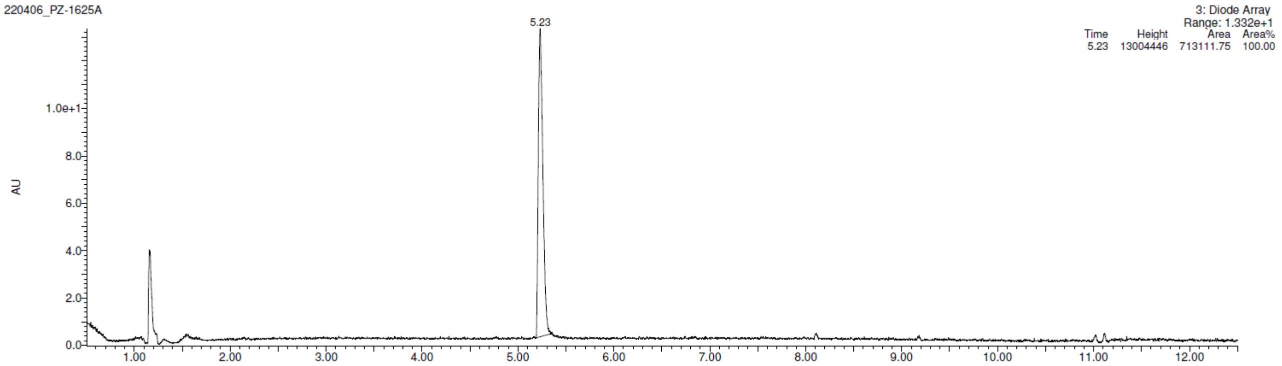

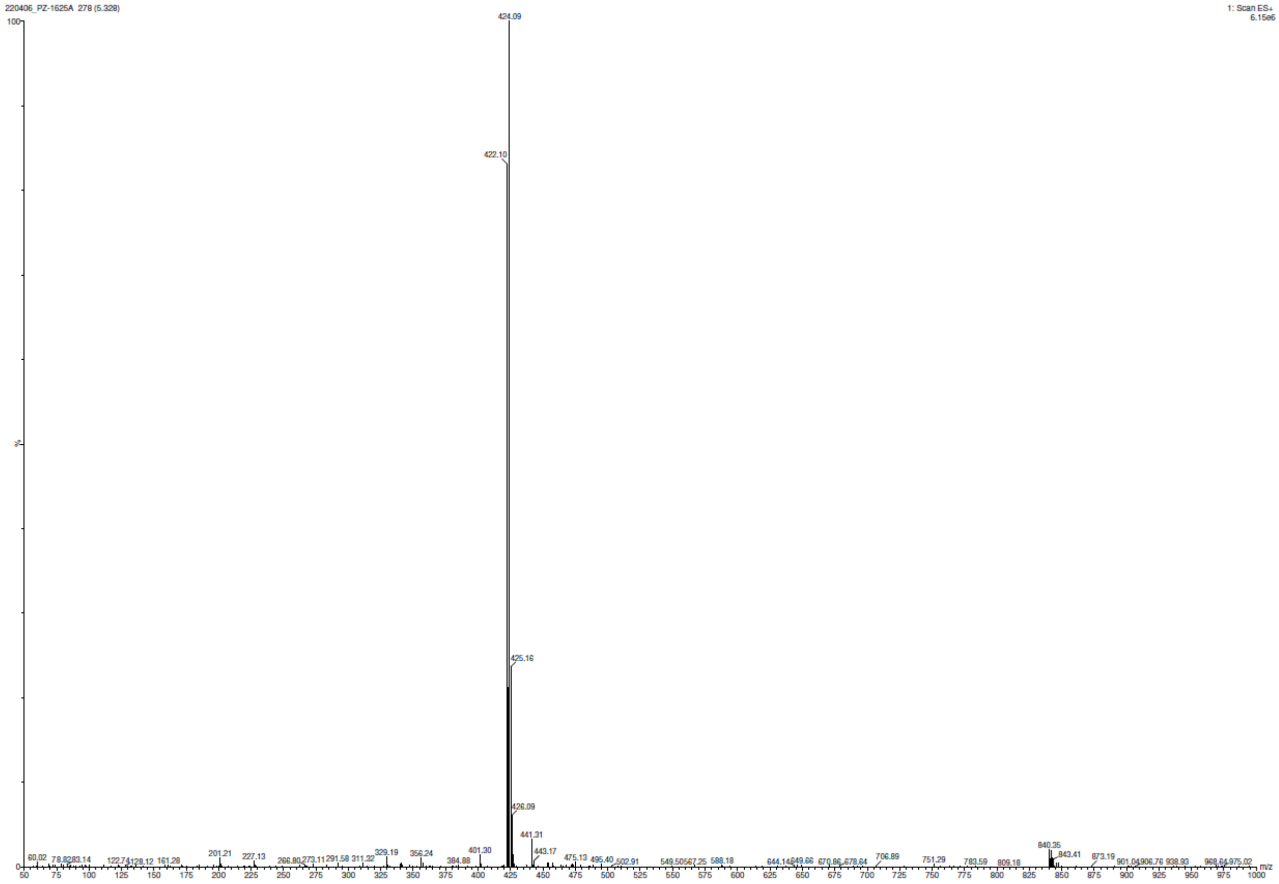

^{1}H-NMR (500 MHz, DMSO-d_6)

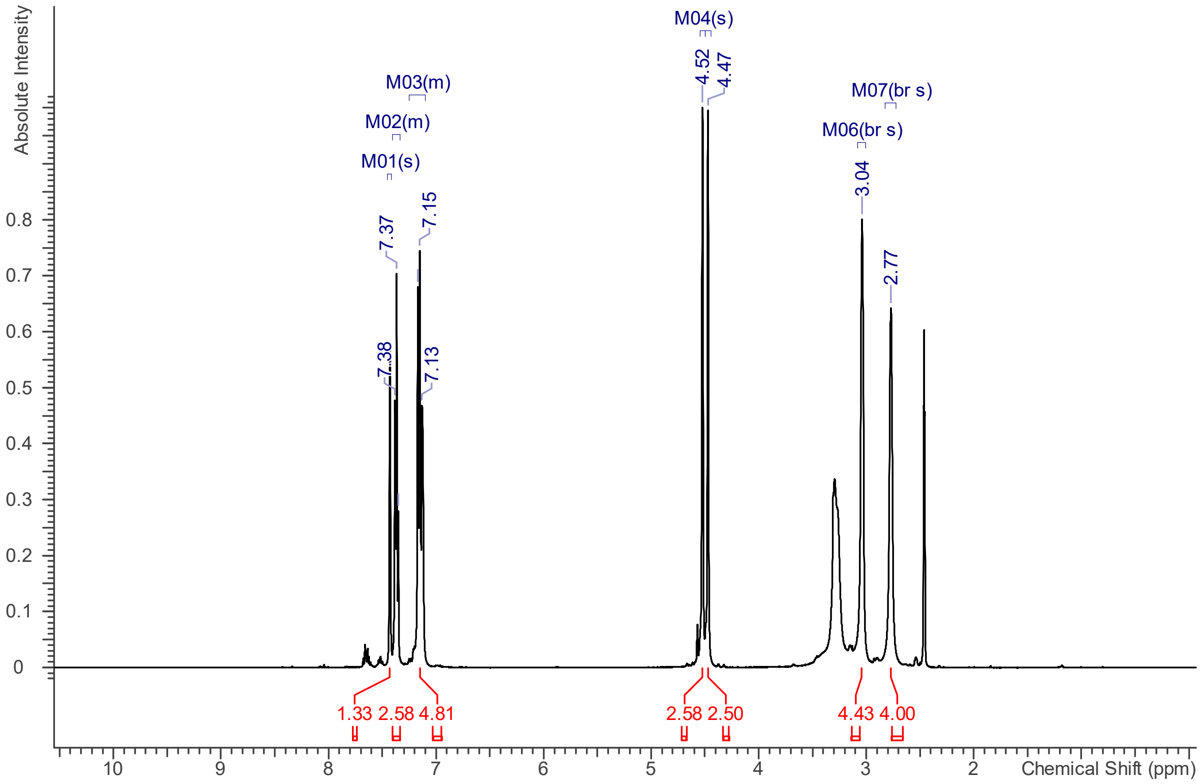

^{13}C-NMR (126 MHz, DMSO-d_6)

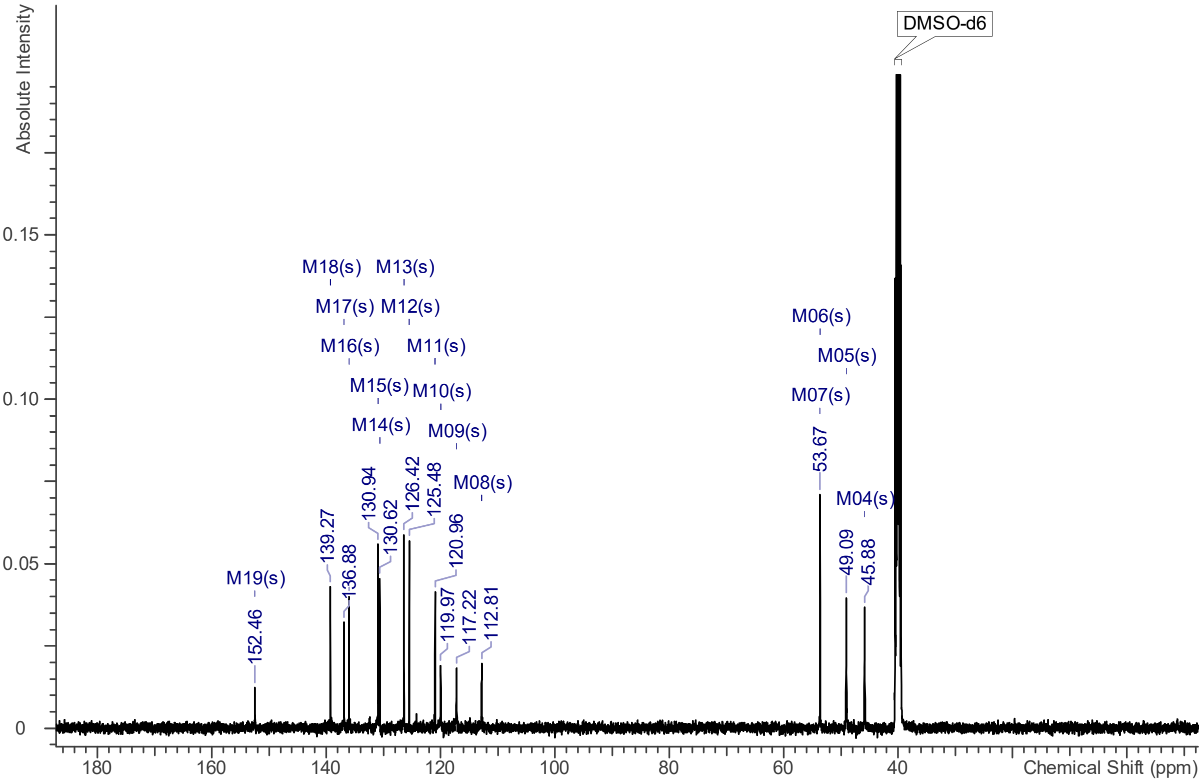

3.9. 2-{[2-(Piperazin-1-yl)phenyl]sulfonyl}isoindoline **3i**

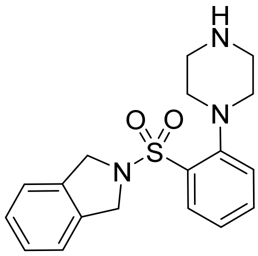

UPLC/MS

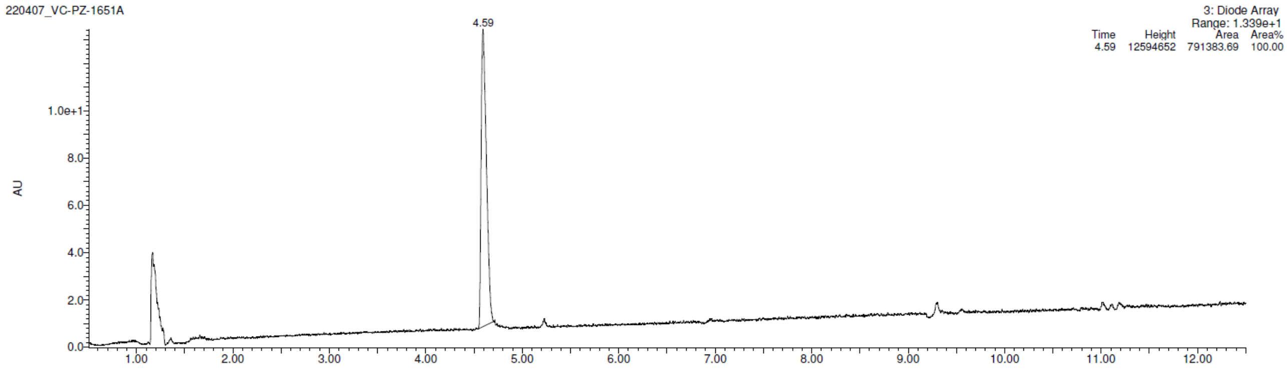

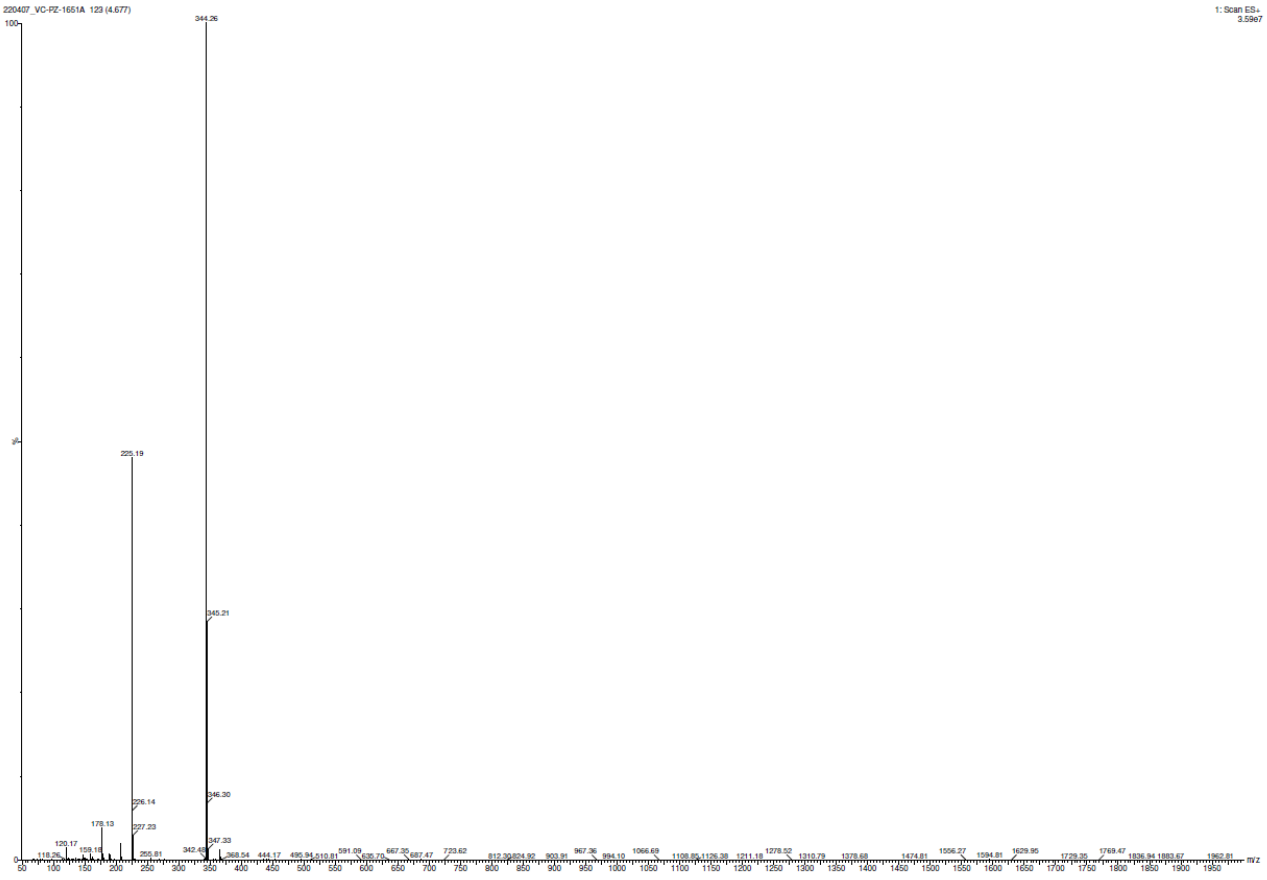

^{1}H-NMR (500 MHz, $CDCl_3$)

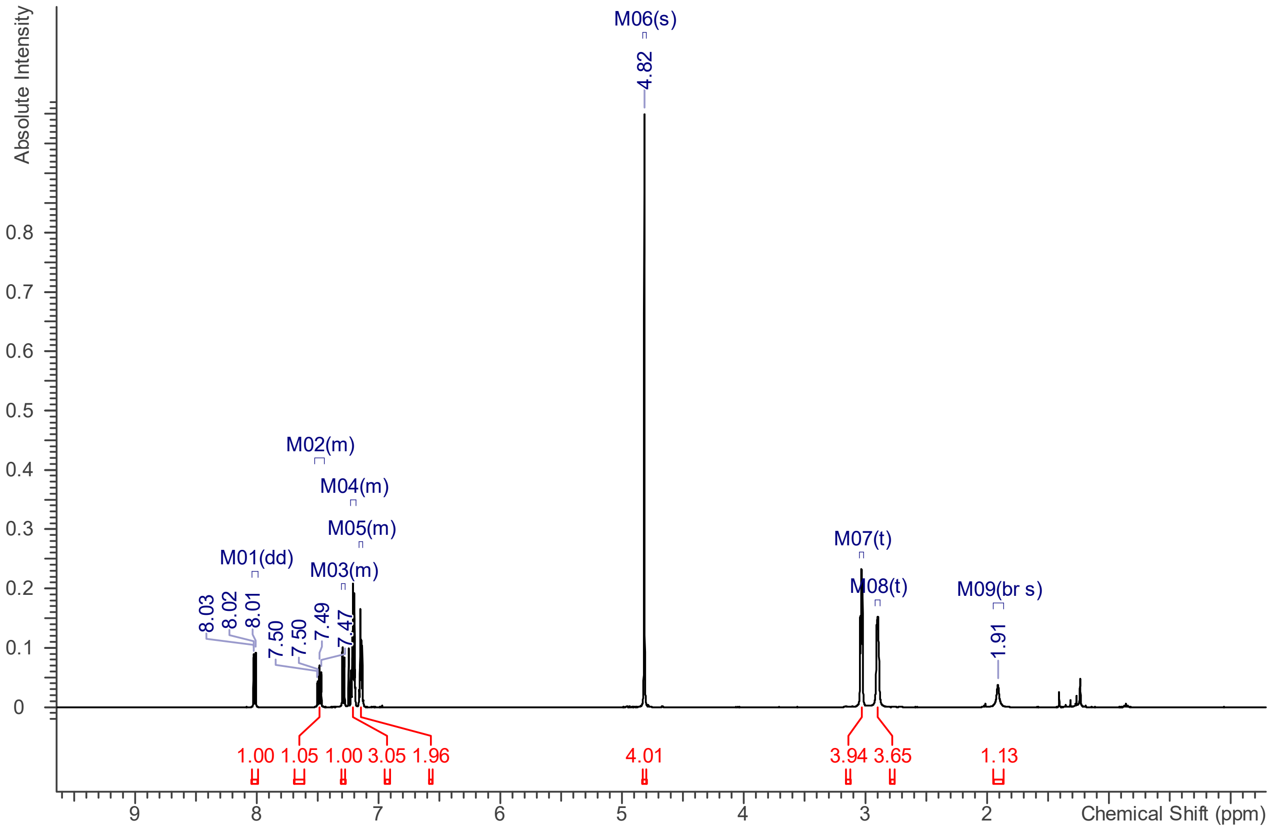

^{13}C-NMR (126 MHz, $CDCl_3$)

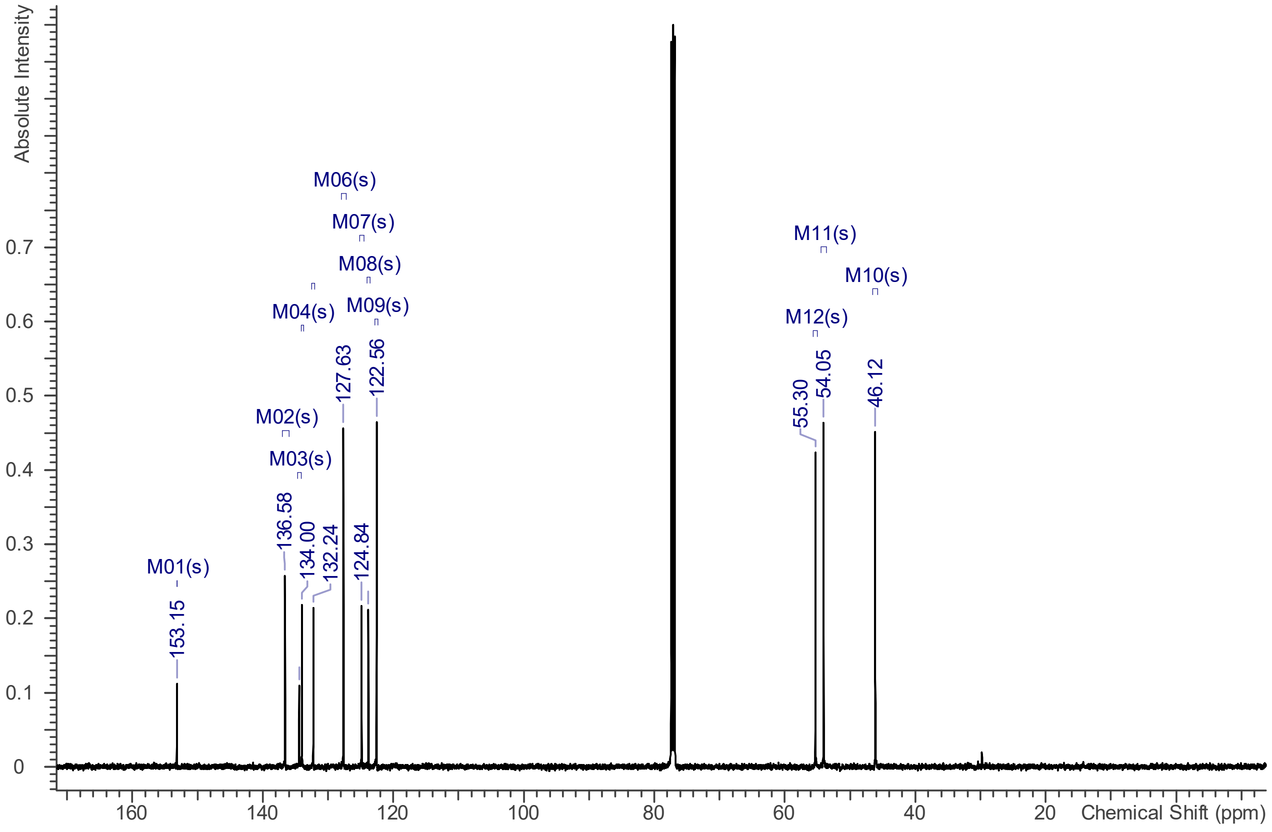

3.10. 4-Chloro-2-{[2-(piperazin-1-yl)phenyl]sulfonyl}isoindoline **3j**

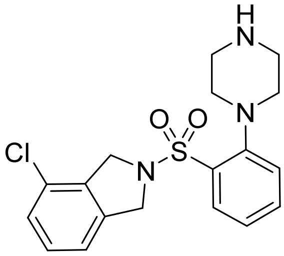

UPLC/MS

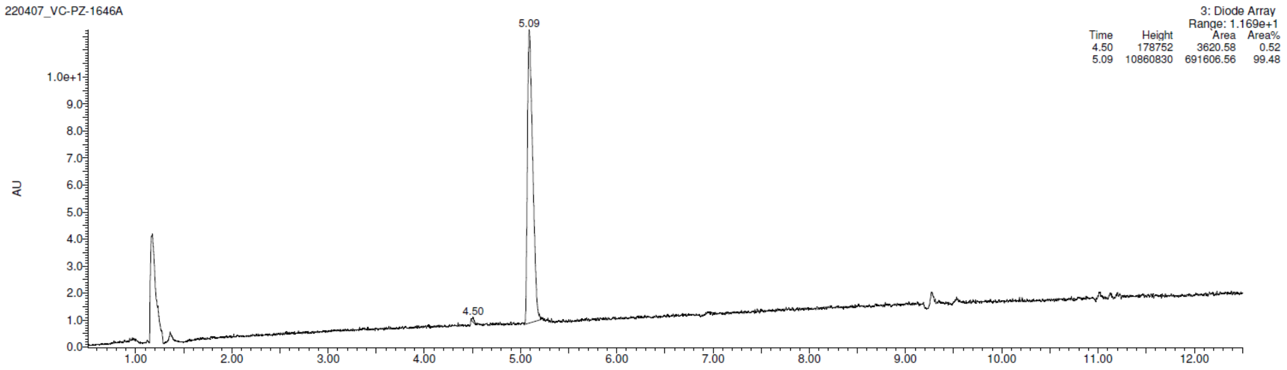

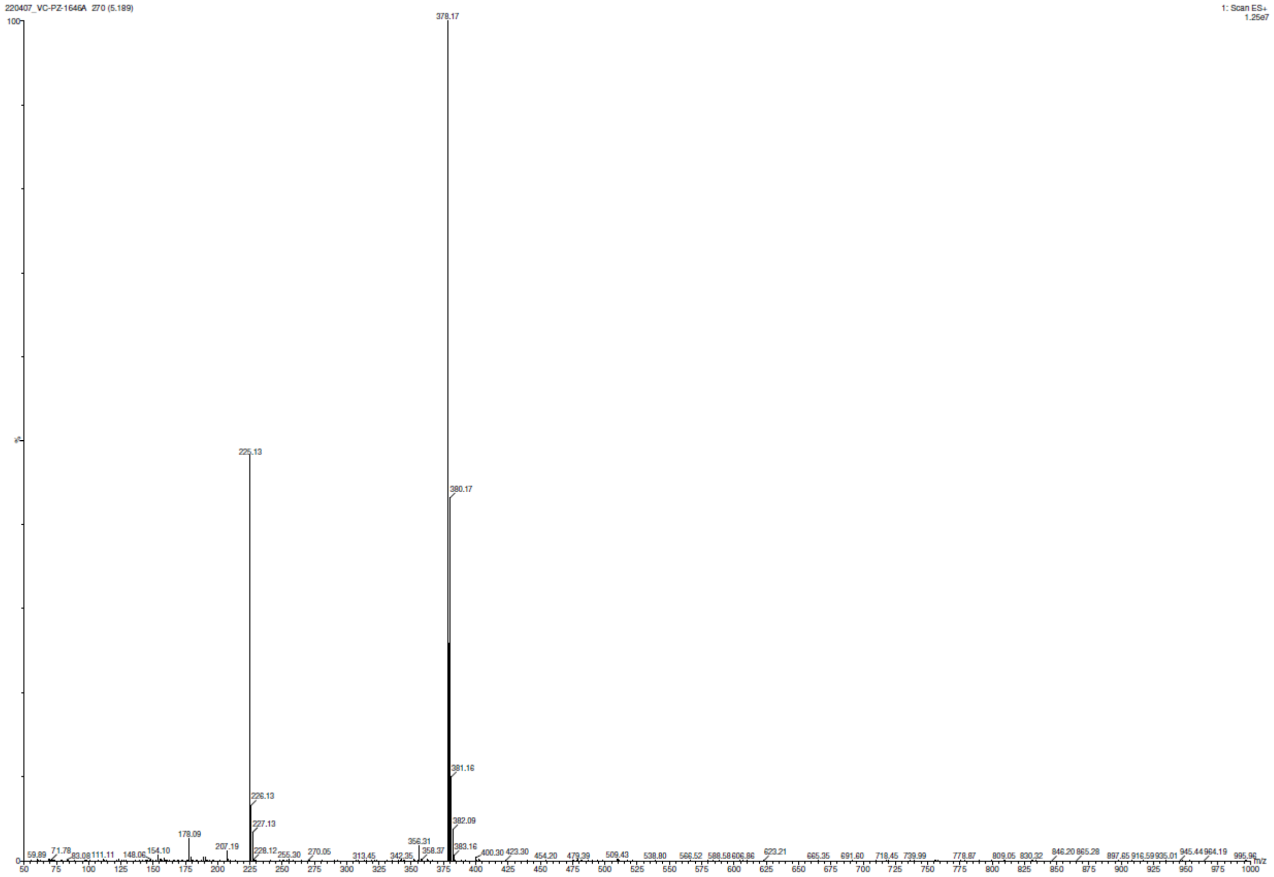

^{1}H-NMR (500 MHz, $CDCl_3$)

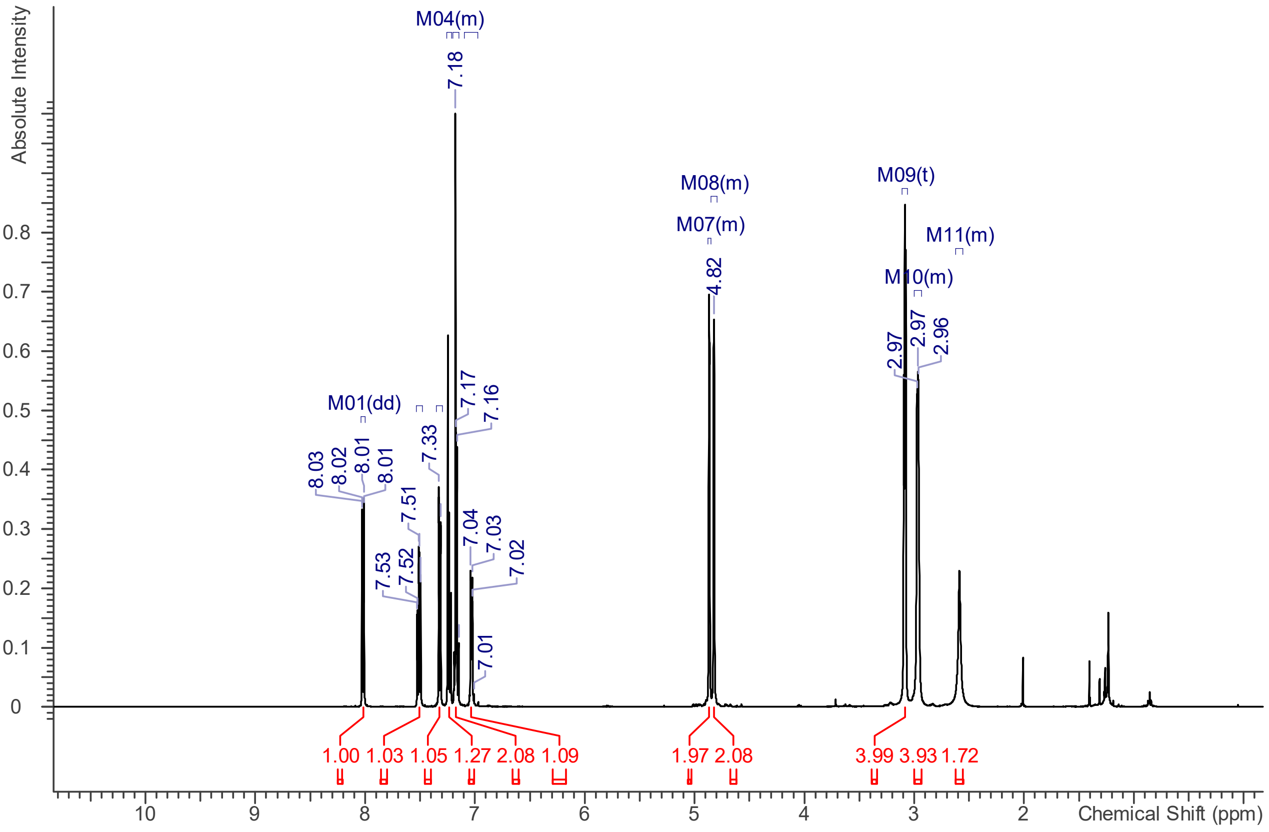

^{13}C-NMR (126 MHz, $CDCl_3$)

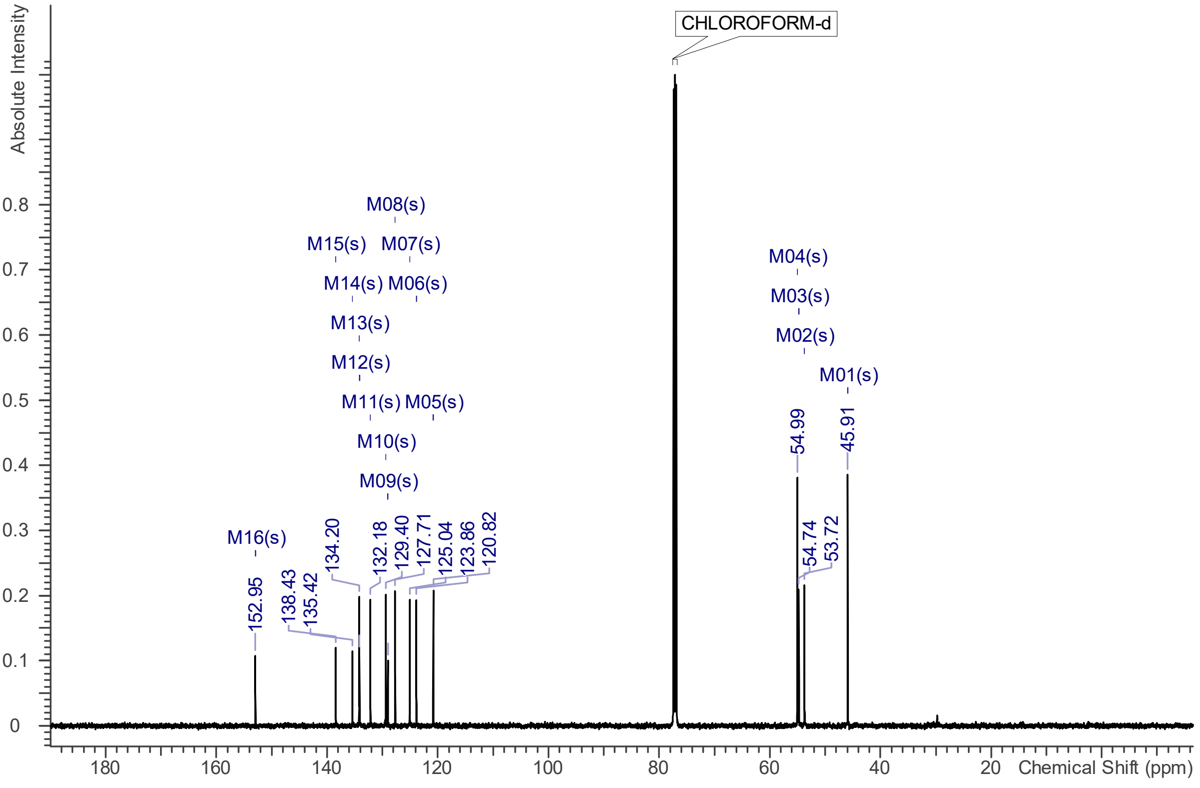

3.11. 4-Bromo-2-{[2-(piperazin-1-yl)phenyl]sulfonyl}isoindoline **3k**

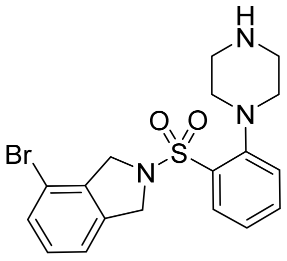

UPLC/MS

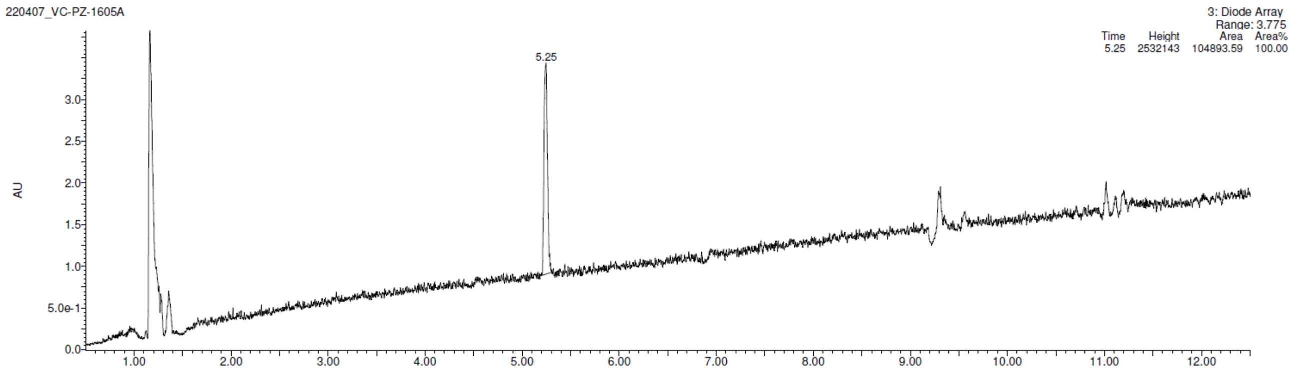

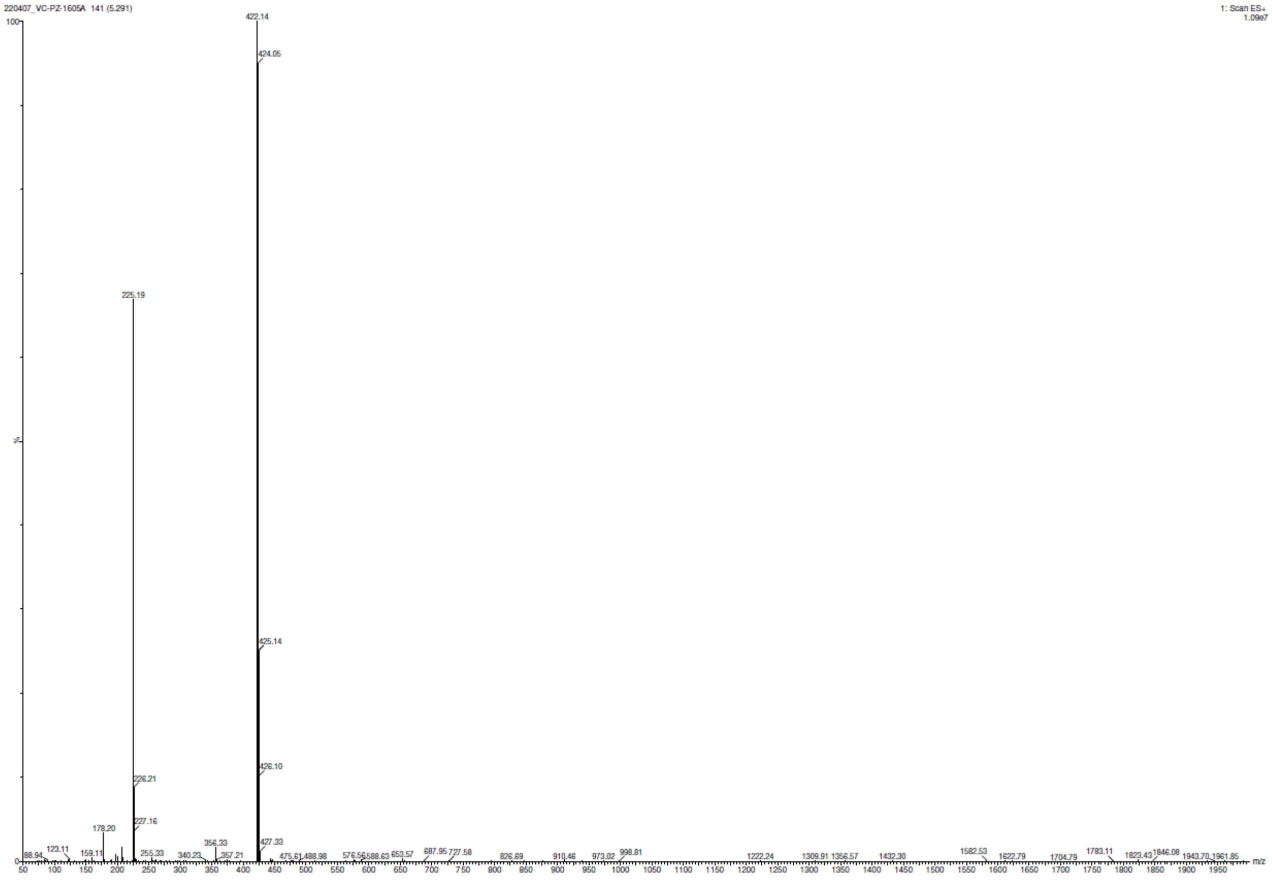

^{1}H-NMR (500 MHz, DMSO-d_6)

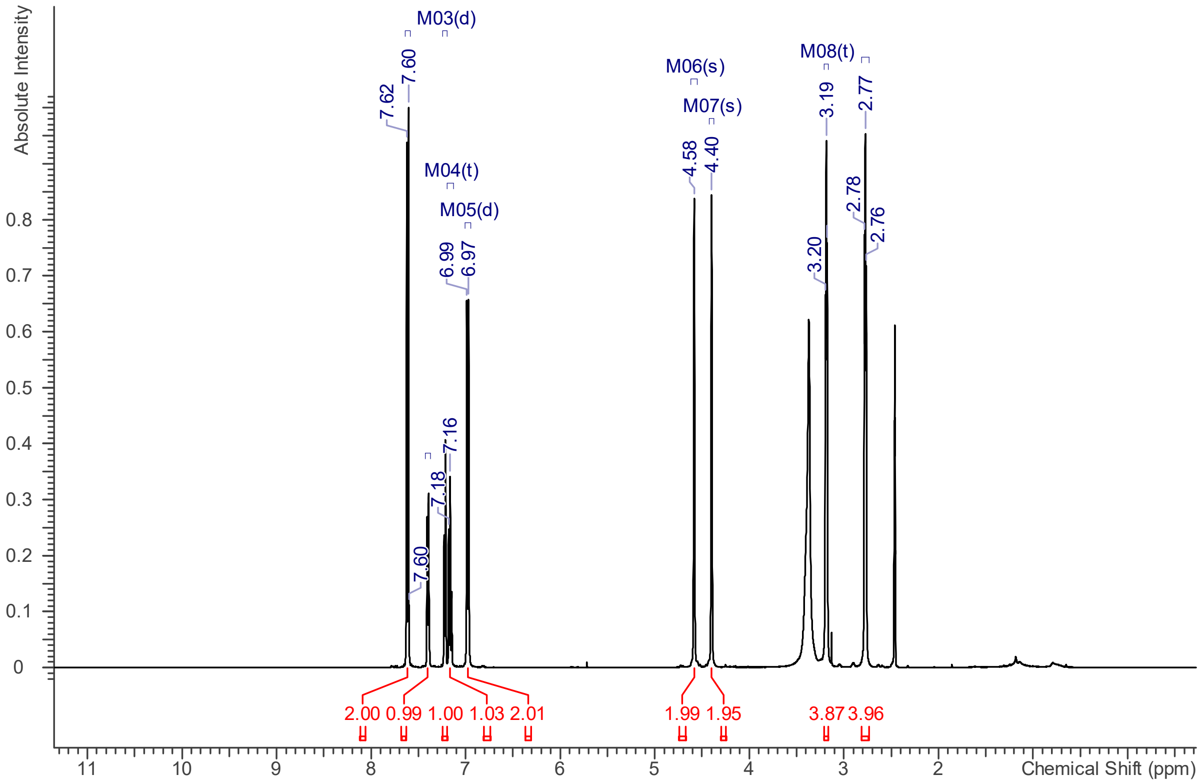

^{13}C-NMR (126 MHz, DMSO-d_6)

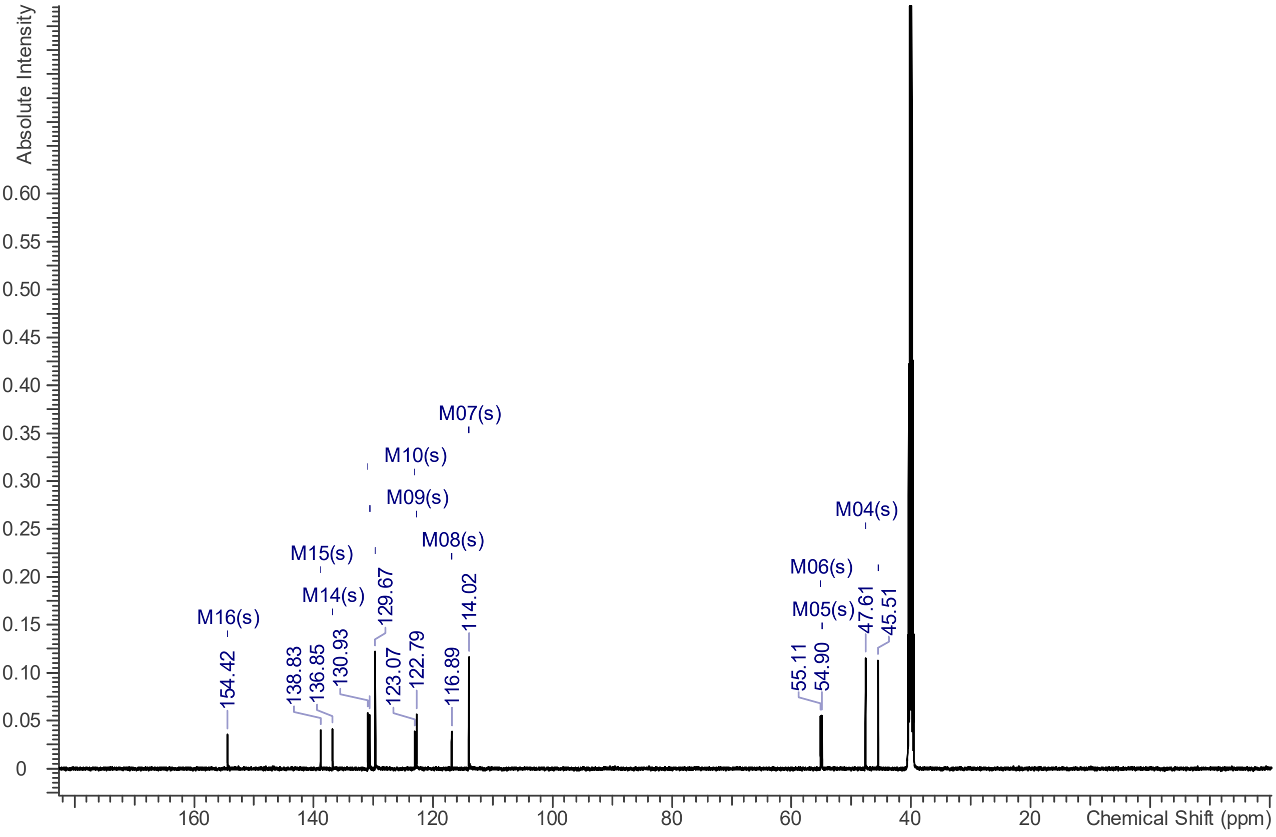

3.12. 5-Bromo-2-{[2-(piperazin-1-yl)phenyl]sulfonyl}isoindoline **3l**

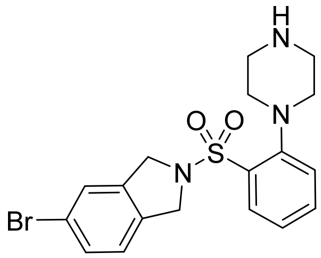

UPLC/MS

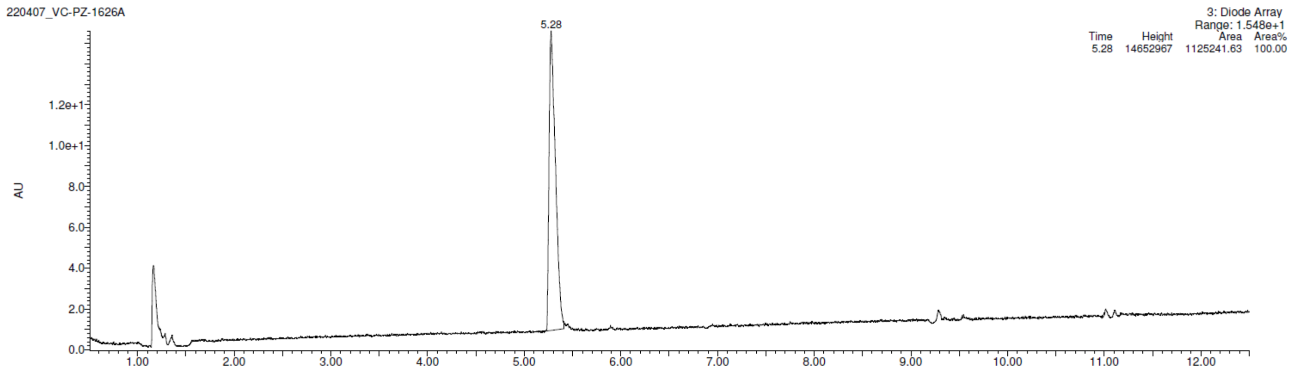

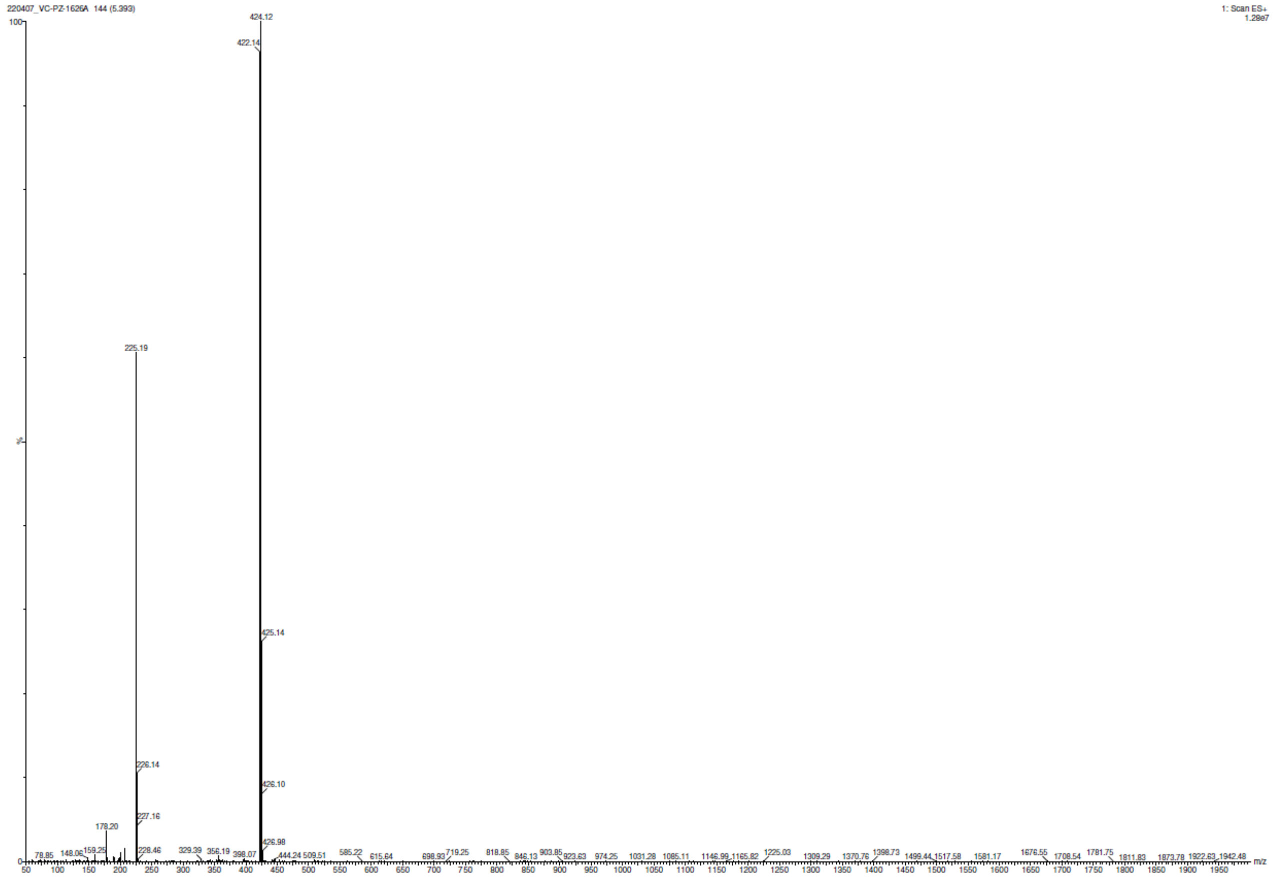

^{1}H-NMR (500 MHz, DMSO-d_6)

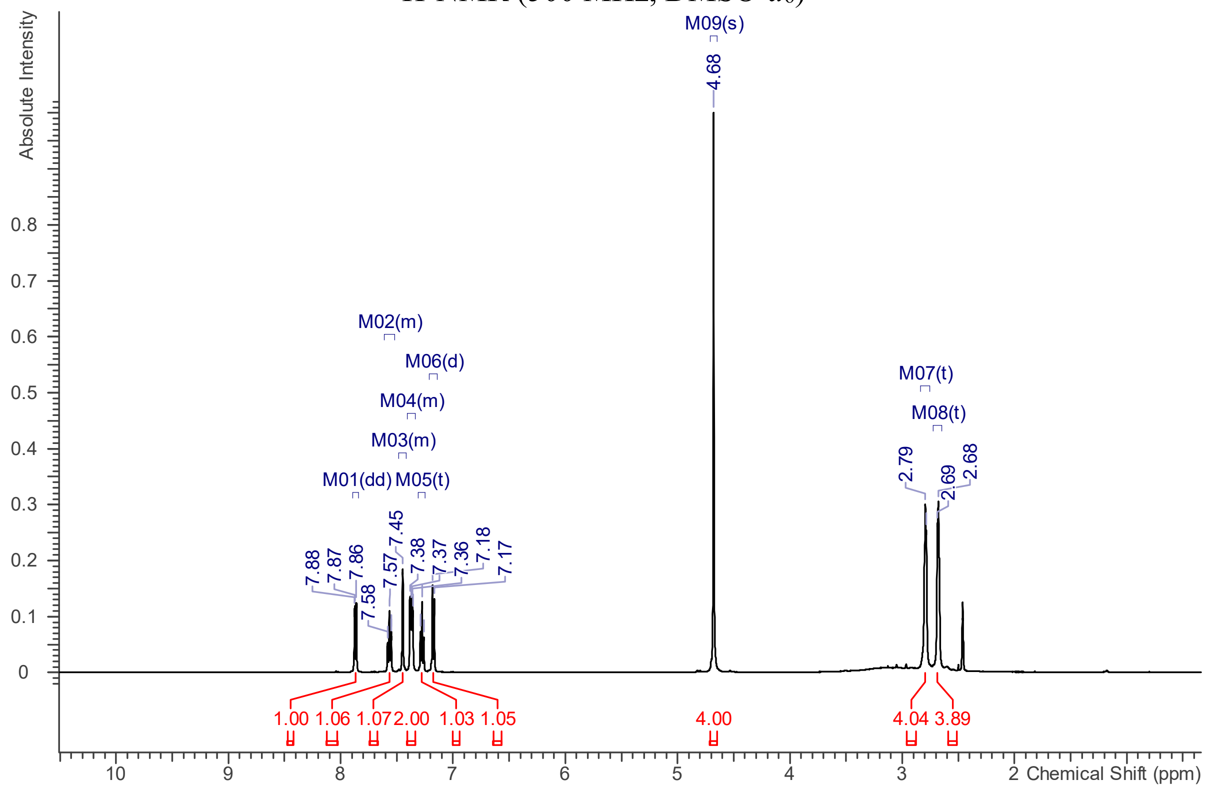

^{13}C-NMR (126 MHz, DMSO-d_6)

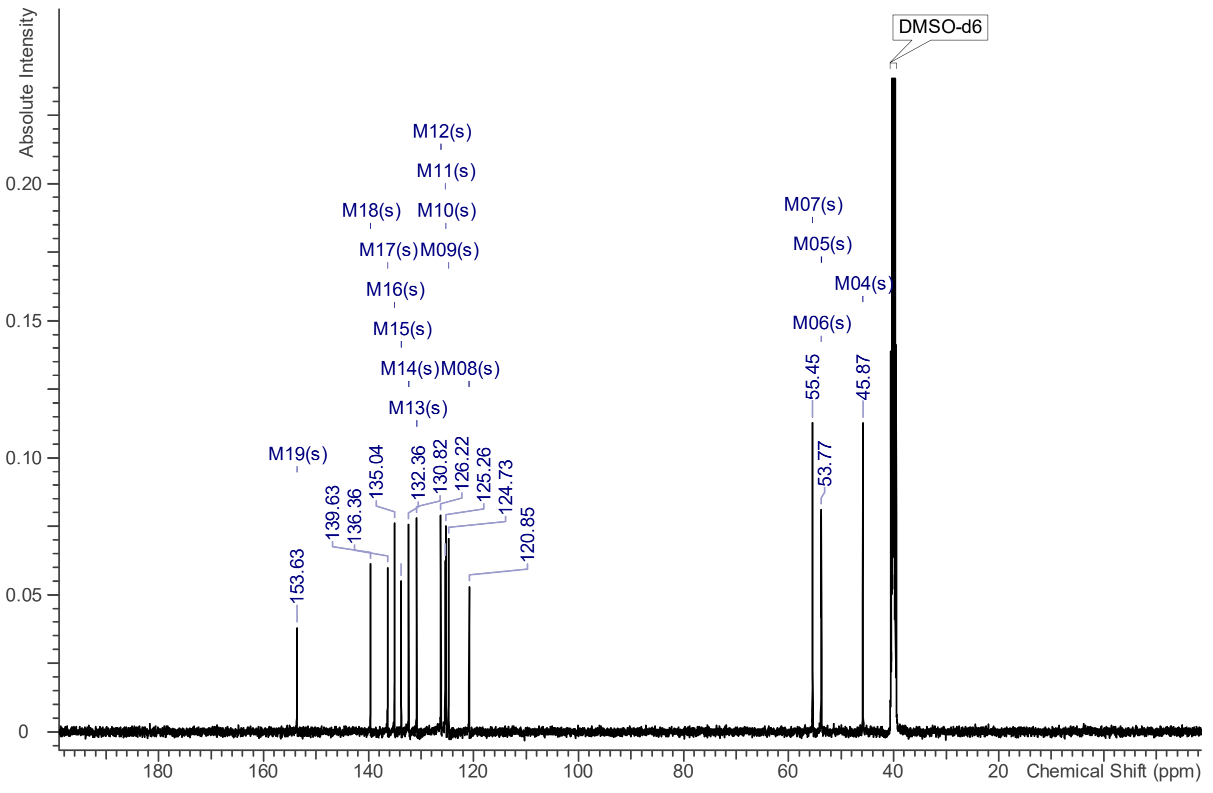

Table S1–SI. Optimization of milling conditions for sulfonylation of isoindolines **1a**–**d**

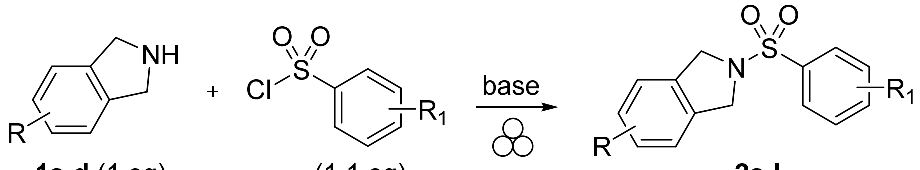

1a-d (1 eq) (1.1 eq)

entry	R	R_1 [eq]	Base [eq]	time [min]	% conversion[a]	% yield[b]	product
**1**[c]	H	4-F	K_2CO_3 (2)	5	97	95	**2a**
**2**[c]	H	4-F	$NaHCO_3$ (2)	5	69	ND	**2a**
**3**[c]	H	4-F	KOH (2)	5	77	ND	**2a**
**4**[c]	H	4-F	NaOH (2)	5	94	92	**2a**
**5**[c]	H	4-F	NaOH (3)	5	100	98	**2a**
**6**[d]	H	4-F	NaOH (3)	5	100	98	**2a**
**7**[d]	4-Cl	4-F	NaOH (3)	7	85	81	**2b**
**8**[d]	4-Br	4-F	NaOH (3)	7	83	81	**2c**
**9**[d]	5-Br	4-F	NaOH (3)	7	89	86	**2d**
**10**[d]	H	3-F	NaOH (3)	5	98	96	**2e**
**11**[d]	4-Cl	3-F	NaOH (3)	7	79	75	**2f**
**12**[d]	4-Br	3-F	NaOH (3)	7	82	79	**2g**
**13**[d]	5-Br	3-F	NaOH (3)	7	87	85	**2h**
**14**[d]	H	2-F	NaOH (3)	5	95	93	**2i**
**15**[d]	4-Cl	2-F	NaOH (3)	7	82	79	**2j**
**16**[d]	4-Br	2-F	NaOH (3)	7	86	82	**2k**
**17**[d]	5-Br	2-F	NaOH (3)	7	91	88	**2l**

Reaction conditions: vbm 30 Hz, ϕ_{ball} = 1.5 cm, [a]Conversions were determined by UPLC, [b]Yields were calculated after filtration and washing with H_2O, [c]10 mL SS jar, total mass of reagents = 85 mg, milling load = 10 mg/mL, [d]35 mL SS jar, total mass of reagents = 350 mg, milling load = 10 mg/mL; ND = not determined.

Table S2–SI. Optimization of milling conditions for aromatic substitution of intermediates **2a–l**

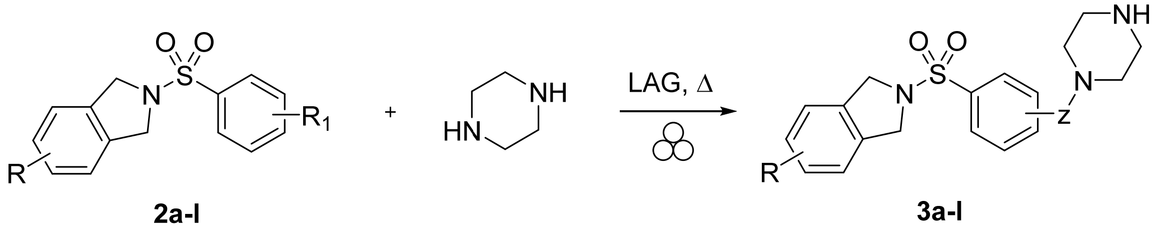

entry	R	R1	LAG [η][a]	T [°C]	time [min]	% conversion[b]	% yield[c]	product
1[d]	H	4-F	-	25	90	0	ND	**3a**
2[d]	H	4-F	-	50	30	24	ND	**3a**
3[d]	H	4-F	-	50	90	55	ND	**3a**
4[e]	H	4-F	MeCN	50	90	72	ND	**3a**
5[f]	H	4-F	MeCN	50	90	90	88	**3a**
6[f]	4-Cl	4-F	MeCN	50	90	90	83	**3f**
7[f]	4-Br	4-F	MeCN	50	90	89	88	**3i**
8[f]	5-Br	4-F	MeCN	50	90	90	86	**3l**
9[f]	H	3-F	MeCN	50	90	59	ND	**3c**
10[f]	H	3-F	DMSO	80	90	78	75	**3c**
11[f]	4-Cl	3-F	DMSO	80	90	80	77	**3e**
12[f]	4-Br	3-F	DMSO	80	90	79	76	**3h**
13[f]	5-Br	3-F	DMSO	80	90	80	78	**3k**
14[f]	H	2-F	MeCN	50	90	88	84	**3a**
15[f]	4-Cl	2-F	MeCN	50	90	86	81	**3d**
16[f]	4-Br	2-F	MeCN	50	90	85	80	**3g**
17[f]	5-Br	2-F	MeCN	50	90	86	82	**3j**

Reaction conditions: vbm 30 Hz, ϕ_{ball} = 1.5 cm, 10 mL SS jar, total mass of reagents = 85 mg, milling load 10 mg/mL, [a]Liquid Assisted Grinding, η = 0.4 µL/mg; [b]Conversions were determined by UPLC, [c]Yield for isolated compound; [d]piperazine anhydrous (1 eq); [e]piperazine anhydrous (2 eq); [f]piperazine anhydrous (3 eq); ND = not determined.

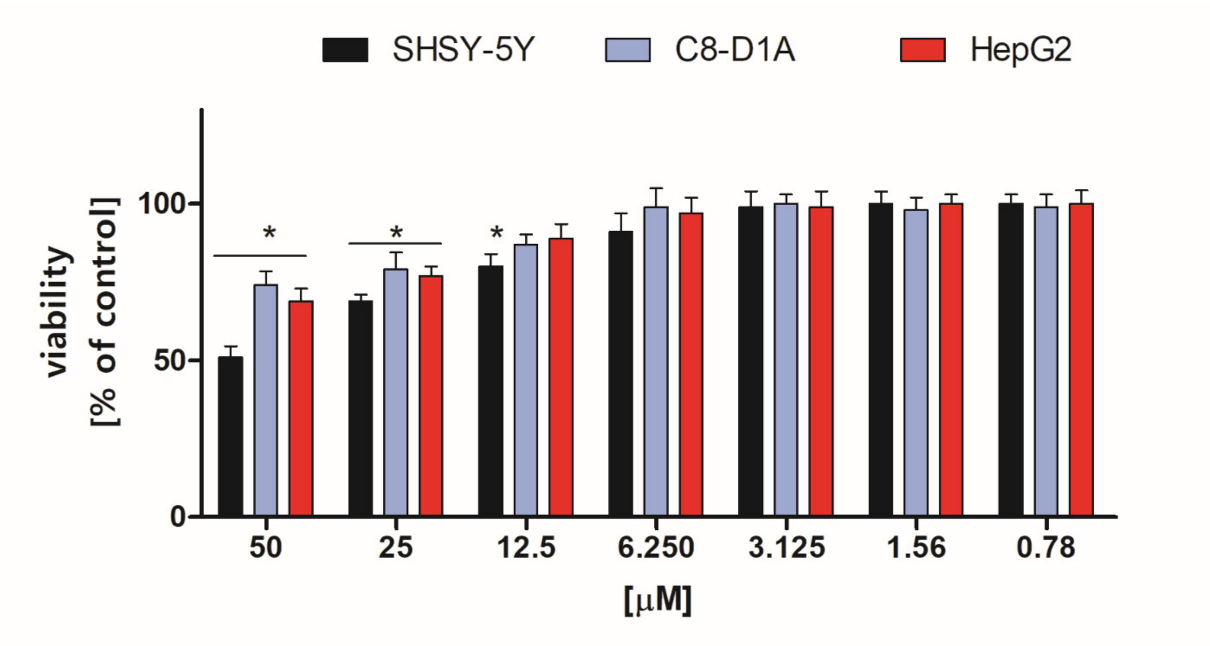

Figure S1–SI. The effect of compound **3g** on cellular viability in (**A**) SH-SY5Y (human neuroblastoma), (**B**) C8-1DA (mouse astrocytes), and (**C**) HepG2 (human hepatocellular carcinoma) cell lines. Cells were incubated in the presence of **3g** in concentration range 0.78–50 μM for 48 hours, then MTT assay was performed to assesses cellular viability. Graph represent cells viability calculated as percent of control (mean ± SD) of three independent experiments. Statistical analysis by one-way ANOVA showed significant differences between the groups ($p < 0.05$) and was followed by the Dunnett's multiple comparisons test, * $p \leq 0.05$.

1. Procedure for radioligand binding assay

All experiments were carried out according to the previously published procedures [1–3]. HEK293 cells stably expressing human 5-HT_{1A}, 5-HT_6, 5-HT_{7b} and D_{2L} receptors (prepared with the use of Lipofectamine 2000) or CHO-K1 cells with plasmid containing the sequence coding for the human serotonin 5-HT_{2A} receptor (Perkin Elmer) were maintained at 37 °C in a humidified atmosphere containing 5% CO_2 and grown in Dulbecco's Modified Eagle's Medium containing 10% dialyzed fetal bovine serum and 500 µg/ml G418 sulfate. For membrane preparation, cells were cultured in 150 cm^2 flasks, grown to 90% confluence, washed twice with pre-warmed to 37 °C phosphate buffered saline (PBS) and centrifuged (200 × g) in PBS containing 0.1 mM EDTA and 1 mM dithiothreitol. Prior to membrane preparation, pellets were stored at –80 °C. Cell pellets were thawed and homogenized in 10 volumes of assay buffer using an Ultra Turrax tissue homogenizer and centrifuged twice at 35,000 × g for 15 min at 4 °C, with incubation for 15 min at 37 °C between the centrifugations. The composition of the assay buffers was experimentally selected to achieve the maximum signal window.

5-HT_{1A}R: 50 mM Tris HCl, 0.1 mM EDTA, 4 mM $MgCl_2$, 10 µM pargyline and 0.1% ascorbate; **5-HT_{2A}R**: 50 mM Tris HCl, 0.1 mM EDTA, 4 mM $MgCl_2$ and 0.1% ascorbate; **5-HT_6R**: 50 mM Tris HCl, 0.5 mM EDTA and 4 mM $MgCl_2$; **5-HT_{7b}R**: 50 mM Tris HCl, 4 mM $MgCl_2$, 10 µM pargyline and 0.1% ascorbate; **D_{2L}R**: 50 mM Tris HCl, 1 mM EDTA, 4 mM $MgCl_2$, 120 mM NaCl, 5 mM KCl, 1.5 mM $CaCl_2$ and 0.1% ascorbate).

All assays were carried out in a total volume of 200 µL in 96-well plates for 1 h at 37 °C, except 5-HT_{1A}R and 5-HT_{2A}R which were incubated at room temperature and 27 °C, respectively. The process of equilibration was terminated by rapid filtration through Unifilter plates with a 96-well cell harvester and radioactivity retained on the filters was quantified on a Microbeta plate reader (PerkinElmer, USA). For displacement studies the assay samples contained as radioligands (PerkinElmer, USA): 2.5 nM [^{3}H]-8-OH-DPAT (135.2 Ci/ mmol) for 5-HT_{1A}R; 1 nM [^{3}H]-ketanserin (53.4 Ci/mmol) for 5-HT_{2A}R; 2 nM [^{3}H]-LSD (83.6 Ci/mmol) for 5-HT_6R; 0.8 nM [^{3}H]-5-CT (39.2 Ci/mmol) for 5-HT_7R or 2.5 nM [^{3}H]-raclopride (76.0 Ci/mmol) for D_{2L}R. Non-specific binding was defined in the presence of 10 µM of 5-HT in 5-HT_{1A}R and 5-HT_7R binding experiments, 20 µM of mianserin for 5-HT_{2A}R, 10 µM of methiothepine for 5-HT_6R or 10 µM of haloperidol for D_{2L}R bidning assays. Each compound was tested in triplicate at 7 concentrations (10^{-10}–10^{-4} M). The inhibition constants (K_i) were calculated from the Cheng-Prusoff equation [4].

2. Procedure for determination of functional activity at Gs signaling

*2.1. Impact of compound **3e**, **3f** and **3g** on cAMP production in 1321N1 cells*

The properties of evaluated compounds to inhibit cAMP production induced by 5-CT (1000 nM), a 5-HT$_6$R agonist, was assessed according to previously published procedures [5,6]. The level of cAMP was measured using frozen recombinant 1321N1 cells expressing the human serotonin 5-HT$_6$R (PerkinElmer). Total cAMP was measured using the LANCE cAMP detection kit (PerkinElmer), according to the manufacturer's recommendations. For quantification of cAMP levels, 2000 cells/well (5 µL) were incubated with mixture of compound **3e**, **3f** and **3g** (5 µL) for 30 min at room temperature in 384-well white opaque microtiter plate. After incubation, the reaction was stopped and cells were lysed by the addition of 10 µL of working solution (5 µL Eu-cAMP and 5 µL ULight-anti-cAMP) for 1 h at room temperature. Time-resolved fluorescence resonance energy transfer (TR-FRET) was detected by an Infinite M1000 Pro (Tecan) using instrument settings from LANCE cAMP detection kit manual. Compound was tested in triplicate at 8 concentrations (10^{-11}–10^{-4} M). K_b values were calculated from Cheng–Prusoff equation specific for the analysis of functional inhibition curves: $K_b = IC_{50}/(1+A/EC_{50})$ where A represents agonist concentration, IC_{50} the concentration of antagonist producing a 50% reduction in the response to agonist, and EC_{50} the agonist concentration which causes a half of the maximal response [4].

1.1.1. Impact of evaluated compounds on cAMP production elicited by constitutively active 5-HT$_6$R in NG108-15 cells

NG108-15 cells were grown in Dulbecco's modified Eagle's medium (DMEM) supplemented with 10% dialyzed fetal calf serum, 2% hypoxanthine/aminopterin/thymidine (Life technologies), and antibiotics. cAMP measurement was performed in cells transiently expressing 5-HT$_6$R using the bioluminescence resonance energy transfer (BRET) sensor for cAMP, CAMYEL (cAMP sensor using YFP-Epac-RLuc) [7]. NG108-15 cells were cotransfected in suspension with 5-HT$_6$R (0.5 µg DNA/million cells) and CAMYEL constructs (1 µg DNA/million cells), using Lipofectamine 2000, according to the manufacturer's protocol, and plated in white 96-well plates (Greiner), at a density of 50,000 cells per well in DMEM containing 2% dialyzed fetal calf serum. After 24 h of transfection, cells were washed with PBS containing calcium and magnesium. To test the functional properties of compound **3e**, **3f** and **3g** as well as the reference SB-258585, cells were treated with vehicle or with the tested compound at a concentration ranging from 0.1 nM to 10 µM. Coelanterazine H (Molecular Probes) was added at a final concentration of 5 µM, and incubated at room temperature for 5 min. BRET was measured using a Mithras LB 940 plate reader (Berthold Technologies). Expression of 5-

HT_6R in NG108-15 cells induced a strong decrease in CAMYEL BRET signal, compared with cells transfected with an empty vector instead of the plasmid encoding the 5-HT_6R. This decrease in CAMYEL BRET signal was thus used as an index of 5-HT_6R constitutive activity at Gs signaling.

3. Procedure for evaluation of the effect of tested compounds on Cdk5-dependent neurite growth

In line with previously reported methods [5,8], NG108-15 cells were transfected with plasmids encoding either cytosolic GFP or a GFP-tagged 5-HT_6R in suspension using Lipofectamine 2000 (Life technologies) and plated on glass coverslips. Six hours after transfection, cells were treated with either DMSO (control), **3e**, **3f** and **3g** or SB-258585 for 48 h. Cells were fixed in 4% paraformaldehyde (PFA) supplemented with 4% sucrose for 10 min. PFA fluorescence was quenched by incubating the cells in PBS containing 0.1 M glycine, prior to mounting in Prolong Gold antifade reagent (Thermo Fisher Scientific). Cells were imaged using an AxioImagerZ1 microscope equipped with epifluorescence (Zeiss), using a 20 × objective for cultured cells, and neurite length was assessed using the Neuron J plugin of the ImageJ software (NIH).

References

1. Kurczab, R.; Canale, V.; Satała, G.; Zajdel, P.; Bojarski, A.J. Amino Acid Hot Spots of Halogen Bonding: A Combined Theoretical and Experimental Case Study of the 5-HT(7) Receptor. *J. Med. Chem.* **2018**, *61*, 8717–8733. https://doi.org/10.1021/acs.jmedchem.8b00828.
2. Partyka, A.; Kurczab, R.; Canale, V.; Satała, G.; Marciniec, K.; Pasierb, A.; Jastrzębska-Więsek, M.; Pawłowski, M.; Wesołowska, A.; Bojarski, A.J.; et al. The Impact of The Halogen Bonding on D(2) and 5-HT(1A)/5-HT(7) Receptor Activity of Azinesulfonamides of 4-[(2-ethyl)piperidinyl-1-yl]phenylpiperazines With Antipsychotic and Antidepressant Properties. *Bioorg. Med. Chem.* **2017**, *25*, 3638–3648. https://doi.org/10.1016/j.bmc.2017.04.046
3. Zajdel, P.; Kos, T.; Marciniec, K.; Satała, G.; Canale, V.; Kamiński, K.; Hołuj, M.; Lenda, T.; Koralewski, R.; Bednarski, M.; et al. Novel Multi-Target Azinesulfonamides of Cyclic Amine Derivatives as Potential Antipsychotics with Pro-Social and Pro-Cognitive Effects. *Eur. J. Med. Chem.* **2018**, *145*, 790–804. https://doi.org/10.1016/j.ejmech.2018.01.002.
4. Cheng, Y.; Prusoff, W.H. Relationship Between the Inhibition Constant (K1) and the Concentration of Inhibitor Which Causes 50 Per Cent Inhibition (I50) of an Enzymatic Reaction. *Biochem. Pharmacol.* **1973**, *22*, 3099–3108. https://doi.org/10.1016/0006-2952(73)90196-2.
5. Vanda, D.; Canale, V.; Chaumont-Dubel, S.; Kurczab, R.; Satała, G.; Koczurkiewicz-Adamczyk, P.; Krawczyk, M.; Pietruś, W.; Blicharz, K.; Pękala, E.; et al. Imidazopyridine-Based 5-HT(6) Receptor Neutral Antagonists: Impact of N(1)-Benzyl and N(1)-Phenylsulfonyl Fragments on Different Receptor Conformational States. *J. Med. Chem.* **2021**, *64*, 1180–1196. https://doi.org/10.1021/acs.jmedchem.0c02009.
6. Drop, M.; Jacquot, F.; Canale, V.; Chaumont-Dubel, S.; Walczak, M.; Satała, G.; Nosalska, K.; Mahoro, G.U.; Słoczyńska, K.; Piska, K.; et al. Neuropathic Pain-Alleviating Activity of Novel 5-HT(6) Receptor Inverse Agonists derived from 2-aryl-1*H*-pyrrole-3-carboxamide. *Bioorg. Chem.* **2021**, *115*, 105218. https://doi.org/10.1016/j.bioorg.2021.105218.
7. Jiang, L.I.; Collins, J.; Davis, R.; Lin, K.-M.; DeCamp, D.; Roach, T.; Hsueh, R.; Rebres, R.A.; Ross, E.M.; Taussig, R.; et al. Use of a cAMP BRET Sensor to Characterize a Novel Regulation of cAMP by the Sphingosine 1-Phosphate/G_{13} Pathway. *J. Biol. Chem.* **2007**, *282*, 10576–10584. https://doi.org/10.1074/jbc.M609695200.
8. Grychowska, K.; Chaumont-Dubel, S.; Kurczab, R.; Koczurkiewicz, P.; Deville, C.; Krawczyk, M.; Pietruś, W.; Satała, G.; Buda, S.; Piska, K.; et al. Dual 5-HT(6) and D(3) Receptor Antagonists in a Group of 1*H*-Pyrrolo[3,2-*c*]quinolines with Neuroprotective and Procognitive Activity. *ACS Chem. Neurosci.* **2019**, *10*, 3183–3196. https://doi.org/10.1021/acschemneuro.8b00618.
